# XPS Peak-Fitting of 2H MoS_2_, 1T MoS_2_, and MoS_2‑X_ Nanosheets in MoS_2_ Powders and Battery Electrodes After Ar^+^ Ion Depth-Profiling

**DOI:** 10.1021/acsanm.5c04608

**Published:** 2026-01-06

**Authors:** Alexandar D. Marinov, Adam J. Clancy, Christopher A. Howard, Patrick L. Cullen

**Affiliations:** † Electrochemical Innovations Laboratory (EIL), Department of Chemical Engineering, 4919 University College London, London WC1E 6BT, U.K.; ‡ CIC EnergiGUNE, Basque Research and Technology Alliance (BRTA), Alava Technology Park, Albert Einstein 48, Vitoria-Gasteiz 01510, Spain; § Department of Chemistry, University College London, London WC1E 6BT, U.K.; ∥ Department of Physics & Astronomy, University College London, London WC1E 6BT, U.K.; ⊥ School of Engineering and Materials Science, Queen Mary University of London, London E1 4NS, U.K.

**Keywords:** SEI, LIB, surface modification, argon ion bombardment

## Abstract

The two common characterization techniques that can distinguish between the metastable 1T and stable 2H phase of molybdenum disulfide (MoS_2_) are Raman spectroscopy and X-ray photoelectron spectroscopy (XPS). Argon ion etching within XPS offers the possibility to explore sample composition as a function of depth. However, for 2H MoS_2_ samples this results in sample alteration via the creation of sulfur vacancies and leads to local areas of MoS_2‑*x*_ and 1T/2H MoS_2_. XPS MoS_2‑*x*_ generation (228.1 eV) and laboratory nanoscale 1T MoS_2_ synthesis (228.4 eV) are easily mistaken in XPS, as both signals exhibit downshifted Mo 3d binding energies relative to 2H MoS_2_ (229.3 eV). Thus, we applied a four split orbit peak XPS model that enables distinction between MoS_2‑*x*_ alteration and successful 1T phase synthesis. In argon etching induced MoS_2‑*x*_ the pristine S 2p peaks remain dominant (162.0 eV) and the S/Mo atomic ratio decreases from 2.5 to 1.1 after electrode ion bombardment. When nanoscale 1T MoS_2_ forms during lithiation in a lithium-ion battery, the dominant S 2p peaks are found at lower binding energies (161.5 eV) and the S/Mo atomic ratio is elevated (>2.0). Depth profiling of ex situ 1T/2H MoS_2_ electrodes showcases lithiation beyond the electrode surface, clearly distinguished from solely MoS_2‑*x*_ alteration through the S 2p component percentage, which exceeds the 1T MoS_2_ limiting threshold (1T > 13% of S 2p) for ion bombarding of as-cast electrodes.

## Graphical abstract

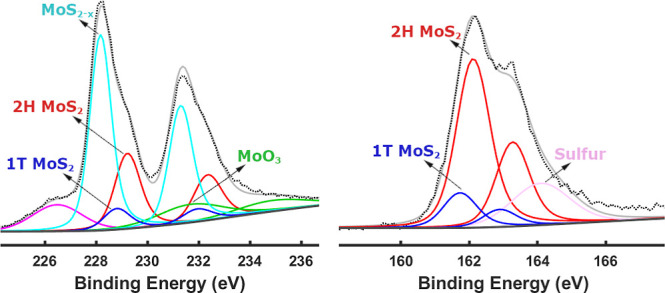

## Introduction

MoS_2_ is a transition metal dichalcogenide abundantly found in the earth’s crust as the mineral molybdenite, with a layered structure.^1^ MoS_2_ possesses three distinct polymorphs (1T, 2H, and 3R), with molybdenite mainly being composed of the 2H phase, while the 1T phase is only produced synthetically^2–4^ on the nanoscale size.^5–8^ The three polymorphs are distinguished by the molecular arrangement of adjacent layers of MoS_2_ (Figure 1) and exhibit very different physical properties.^9,10^ 1T MoS_2_ is metastable and metallic, whereas the naturally abundant 2H polymorph is semiconducting.^10^ The properties of 1T MoS_2_ nanosheets make them of interest across a broad range of energy applications,^11^ including reversible batteries,^12,13^ supercapacitors,^14,15^ and hydrogen evolution reaction catalysis.^16–18^

**Figure 1:**
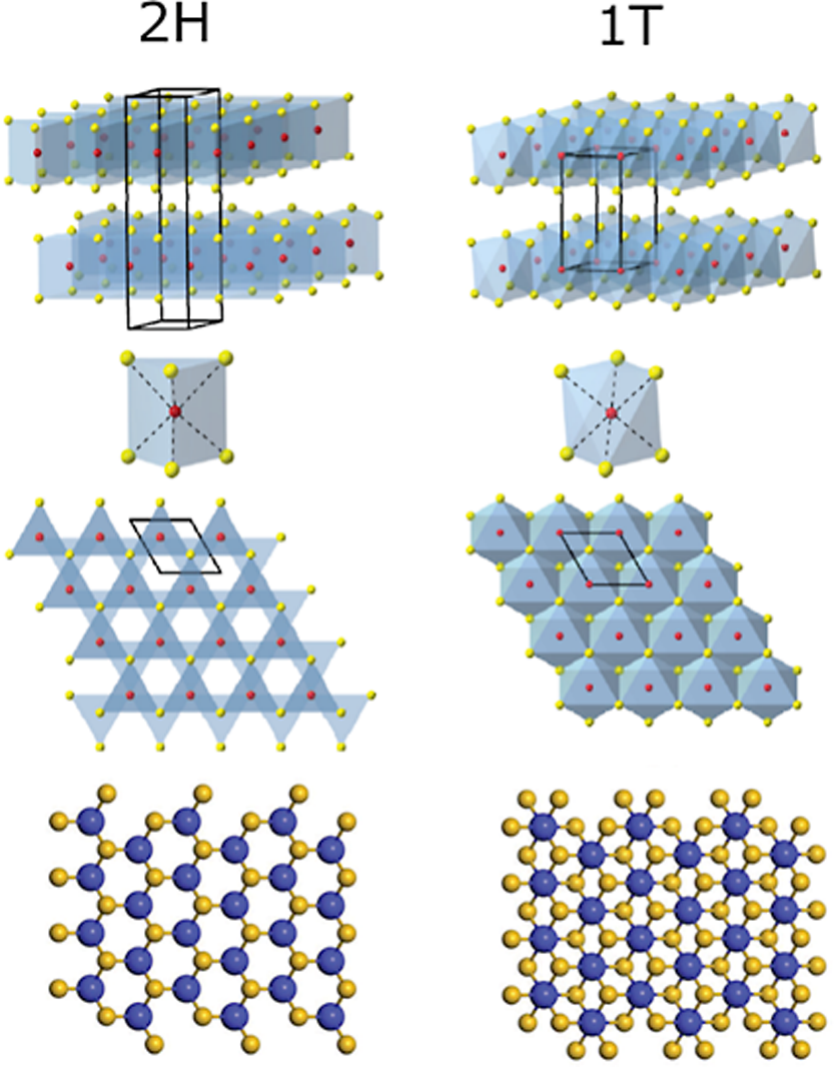
Schematic of the difference in atomic arrangement of the 2H and 1T MoS_2_ phases. 2H MoS_2_ has a two-layer hexagonal unit cell, whereas 1T MoS_2_ has a one-layer trigonal prismatic unit cell. In 2H MoS_2_ adjacent nanosheets (S–Mo–S) are misaligned, while in 1T MoS_2_ adjacent nanosheets align. For a single nanosheet of 2H MoS_2_ the top layer of sulfur overlaps with the lower layer, thus concealing it from view and resulting in the observation of a hexagonal pattern when viewed from the *c*-axis plane. On the other hand, in a 1T MoS_2_ nanosheet the top and bottom layer of sulfur misalign, resulting in Mo atoms appearing to be surrounded by six sulfur atoms when viewed from the *c*-axis plane.^11^ Figure adapted with permission from source.^4,26^

With the rapid increase of papers focusing on the energy device performance of MoS_2_ (37,347 papers from 2010 to 2022),^11^ understanding the material production method or modification leading to enhanced application performance becomes of great importance. Therefore, we previously suggested a base characterization fingerprint for MoS_2_, including scanning electron microscopy (SEM), X-ray diffraction (XRD), X-ray photoelectron spectroscopy (XPS), and Raman spectroscopy due to their wide availability and prevalent literature data for comparison.^11^ However, out of the above characterization techniques only XPS and Raman spectroscopy can distinguish between the 2H and 1T MoS_2_ polymorphs. Unfortunately, as laser penetration depth in MoS_2_ is limited (∼35 nm),^19^ Raman spectroscopy of MoS_2_ electrodes is surface sensitive. On the other hand, XPS can make use of ion bombardment to analyze sample composition changes with depth-profiling (Figure S1).

In lithium-ion batteries (LIBs), MoS_2_ research revolves around the manufacturing and electrochemical testing of thin (10 μm–50 μm) or thick (50 μm–250 μm) 2H^20,21^ or 1T/2H^22^ phase nanoscale material-based electrodes. During electrochemical operation, the electrolyte and electrode come into contact at the surface-electrolyte interphase (SEI) where both materials decompose to form a complex heterogeneous SEI layer, that protects the active materials in the electrode bulk from contact with the reactive electrolyte and thus further degradation.^23–25^ Due to the distinct chemical processes occurring at the electrode surface and deeper within the electrode bulk,^27^ XPS depth-profiling is uniquely suited to study the difference between the SEI layer and the bulk features of battery electrodes.

Within XPS 2H MoS_2_ is identified by the Mo^4+^ 3d_5/2_ (229.2 eV), Mo^4+^ 3d_3/2_, S^2–^ 2s, S^2–^ 2p_3/2_ (162.0 eV), and S^2–^ 2p_1/2_ peaks.^11^ When 1T MoS_2_ is chemically synthesized, the XPS signal involves a binding energy downshift of the Mo 3d peaks (∼0.9 eV),^11^ which is reported widely throughout the energy application literature.^15,28,29^ However, since the 1990s it has been demonstrated that argon ion (Ar^+^) etching of thin films (∼2 nm) of MoS_2_ results in a binding energy downshift of the 2H MoS_2_ XPS peaks toward values normally attributed to 1T phase formation.^30^ We shall refer to this phenomenon as sample alteration. Consequent studies have also reported the deviation of Mo 3d XPS peaks in single crystal or polycrystalline 2H phase MoS_2_ under bombardment with Ar^+^,^31,32^ He^+^,^31,32^ chalcogen ions,^34^ Xe^+^,^31,32^ and even X-rays.^36^ S 2p binding energy downshifts after bombardment are less commonly reported.^30,35,36^

The downshift in the Mo 3d and S 2p peaks following Ar^+^ bombardment is accompanied by a reduction in the S/Mo atomic ratio of the material, from 2.2 in the pristine powder to 1.4 in the altered material.^30,31,33,35^ Hence, the above ion bombardment studies agree that local sulfur vacancies are created within the MoS_2_ network following bombardment,^31,33,37,38^ resulting in the formation of an overall reduced composition MoS_2‑*x*_. However, in application-oriented research (e.g. field effect transistors^39^ and sodium-ion batteries^40^) and material production studies,^41^ argon bombardment of 2H MoS_2_ is considered to produce 1T phase via the induction of small amounts of localized S-vacancies.^39–41^ Since the XPS signatures of 1T phase MoS_2_ and argon bombardment induced MoS_2‑*x*_ significantly overlap, a methodology to clearly distinguish between the two materials is necessary to avoid confusion during sample analysis.

Previous studies have fit Ar^+^ bombarded MoS_2_ with two split peak models considering the presence of 2H MoS_2_ and MoS_2‑*x*_ ^31,32,36^ or 1T MoS_2_.^40^ In all cases only one additional set of peaks is considered for the downshifted Mo 3d peaks (∼228.2 eV).^30–32^ However, to the best of our knowledge the possibility of 1T MoS_2_ and MoS_2‑*x*_ cooccurring with depth-profiling has not been considered. Furthermore, synthetically produced 1T/2H MoS_2_ like that generated by lithiation of MoS_2_ electrodes in LIBs^21^ has not been explored with depth-profiling. In such a situation the lack of a clear method to distinguish between synthetic 1T MoS_2_ and ion bombardment induced MoS_2‑*x*_ restricts analysis to the electrode surface, as previously electrode bulk lithiation could not be confirmed due to sample alteration.^21^

Therefore, herein we conducted argon depth-profiling on pristine 2H MoS_2_ powders and as-cast 2H MoS_2_ battery electrodes to establish the ion bombardment baseline for our system and then apply the same methodology to depth-profiling data from lithiated 1T/2H MoS_2_ ex situ LIB electrodes.^21^ We apply a four split orbit peak model to altered samples that considers the presence of 2H MoS_2_ (POS-A), 1T MoS_2_ (POS-B), MoS_2‑*x*_ (POS-C), and MoO_3_ (POS-D). Depth-profiling as-cast 2H MoS_2_ electrodes identifies a significant portion of MoS_2‑*x*_ (228.1 eV; 49.9% Mo 3d), a smaller fraction of 1T MoS_2_ (228.7 eV; 8.7% Mo 3d), and the reduction of the S/Mo atomic ratio from 2.5 to 1.1 after ion bombardment. In lithiated 1T/2H ex situ MoS_2_ LIB samples the surface already contains downshifted 1T Mo 3d (228.4 eV) and S 2p (161.5 eV) peaks, enabling distinction from MoS_2‑*x*_ alteration (228.1 eV and 162.0 eV). When depth-profiling ex situ MoS_2_ LIB samples the S 2p region becomes critical to infer the electrodes’ phase underneath the SEI surface layer. The composition of sulfur belonging to either 2H MoS_2_/MoS_2‑*x*_ or 1T MoS_2_ allows for phase differentiation, as ion bombardment of as-cast electrodes only produces 1T MoS_2_ up to a S 2p threshold of 13%. Therefore, we were able to confirm the lithiation of the electrode bulk beyond the surface layer in MoS_2_ LIB coin cells.

## Experimental Methods

### Materials

Commercial (top-down precursor) MoS_2_ powder (∼6 μm flake) was purchased from Sigma-Aldrich (CAS: 1317-33-5). Carbon black additive (Super P, 99+%) was purchased from Alfa Aesar (CAS: 1333-86-4). PVDF (polivinylidene fluoride) with the commercial name Solef was purchased from Solvay. Anhydrous 2-methyl-pyrrolidone (NMP) was purchased from Sigma-Aldrich (CAS: 872-50-4). Copper foil was purchased from Cambridge Energy Solutions, with an average thickness of 11 μm. 1 M LiPF_6_ in EC/DMC (3:7 vol.) LIB electrolyte was purchased from PuriEL under the commercial name of SoulBrain MI.

### MoS_2_ Electrode Manufacture

As-cast MoS_2_ electrodes were fabricated with a coating composition of 80:10:10 wt % of MoS_2_, Super P, and PVDF, respectively, as detailed previously.^21^ In a typical process (Figure S2), a 5 wt % PVDF/NMP solution was made first by dispersing 3.0 g of PVDF in 57 mL of NMP (≈58.7 g) by magnetic stirring for 24 h at 400 rpm. Then 100 mg of MoS_2_ powder, 12 mg of Super P powder, and 280 mg of PVDF (5 wt %)/NMP mixture (≈14 mg PVDF) were mixed with a planetary mixer (THINKY ARE-250). A two-step mixing process was applied including 0.5 min at 500 rpm to degas the mixture and 15 min at 1500 rpm to homogenize the slurry. The slurry was then coated onto copper foil (11 μm) using a doctor blade and an automatic film coater (Elcometer 4340). The as-cast electrode was dried on a hot plate at 60 °C until the coating was visibly dry. 15 mm diameter electrode discs were cut and dried further under vacuum in a Buchi tube at 60 °C for 24 h and then taken into a glovebox (O_2_, H_2_O < 0.5 ppm) for coin cell assembly.

### Ex Situ LIB Lithiated Electrodes

MoS_2_ electrodes (∼36μm ± 4.6 μm thick, active material ∼3.43 mg/cm^2^ ± 0.13 mg/cm^2^, & 15 mm diameter) were assembled in Hohsen CR2032 lithium metal half-cells as reported previously.^21^ Cells include a 19 mm diameter and 25 μm thick Celgard separator, 100 μL of 1 M LiPF_6_ in EC/DMC (3:7 vol.) electrolyte, a lithium metal counter electrode (MTI Corporation) with a larger diameter than the working electrode, and a 1 mm thick spacer. Coin cells were assembled and disassembled in a glovebox (O_2_, H_2_O < 0.5 ppm). Cycling was conducted on a BioLogic BCS cycler at a current density of 200 mA/g at room temperature, within a voltage range of 3.00–0.80 V or 3.00–0.01 V. The ex situ electrodes were recovered via coin cell disassembly inside a glovebox within 20 min of completing their final electrochemical step. All cells were opened within the same 1 h time window. Each ex situ electrode was washed separately in fresh anhydrous dimethoxyethane (DME) to remove any remnants of LiPF_6_ salt. Ex situ electrodes were stored in the glovebox to avoid exposure to air or moisture.

### X-ray Diffraction & Raman Spectroscopy

X-ray diffraction patterns were collected in reflex geometry on a Bruker D2 Phaser benchtop XRD kit, with a Cu source tube (∼1.541 Å) operated at 30 kV and 10 mA. Samples were placed on a Si holder. An XRD blade gap of 3.00 mm was used for powders and 1.00 mm for electrodes. All samples were scanned from 1 – 80°, with a 2.00 mm incident slit, a 0.01° step increment, and 0.5 s/step exposure.

Raman spectroscopy was carried out in a Renishaw In-Via microscope with a 785 nm laser (300 mW), a × 50 short distance working objective, and a 1200 L mm^–1^ diffraction grating. Raman measurements were conducted at 1% power, with 3 accumulations per scan, and 10s exposure per accumulation. Raman spectroscopy data was normalized relative to the maximum intensity of each data set. Figures were split at 500 cm^–1^ for readability, so that the MoS_2_ E^1^_2g_ and A_1g_ modes do not dominate the less intense peaks at the lower and higher end of the spectrum.

### XPS & Argon Etching

XPS was carried out on a K-Alpha ThermoFisher Scientific spectrometer (Table S1) with a monochromatic Al *K*_α_ source (1486.68 eV) operated at 12 kV and 6 mA to give a power of 72 W. The X-ray beam had a 400 μm by 400 μm size and the takeoff angle of the electrons was 30°. All scans were conducted with charge compensation via an Ar flood gun (100 μA current; 1 eV energy) to balance the sample surface. The spectrometer ultrahigh vacuum environment was maintained at approximately 2.5e^–7^ mbar during XPS analysis. Survey spectra were conducted with 5 cumulative scans, a pass energy of 200 eV, and step increments of 1.0 eV. Individual elements were sampled with 10 cumulative scans, a pass energy of 50 eV, and step increments of 0.1 eV.

Depth-profiling/etching was carried out via argon ion (Ar^+^) bombardment within the XPS spectrometer with an EX06 ion source (10 μA; 3 keV), a raster width of 2 μm, a 30° gun angle, and a sputter rate of 0.6756 nm/s (Ta_2_O_5_). Sputtering was conducted for 2 – 7 levels per sample with either 10s, 60s, or 100s of exposure per level. In all figures the etch depth is labeled according to the total cumulative exposure time.

Powder and electrode samples were vacuum-dried at 60 °C for 24 h prior to XPS measurements, and either transferred directly into the spectrometer antechamber or stored under an inert glovebox environment (O_2_ & H_2_O < 0.5 ppm) to minimize air exposure. Air-sensitive ex situ lithiated samples were transferred with a custom air-free stage directly from the glovebox into the XPS spectrometer antechamber without exposure to air or moisture. All samples were attached to the XPS holder with carbon tape.

A total of three powder samples, two as-cast electrodes, and nine ex situ electrode segments were studied. For the ex situ electrodes, in each segment three surface scans were first carried out and then one instance of depth-profiling was conducted.

### XPS Peak Fitting

XPS data was fit in CasaXPS. The fitted data was exported as a.csv file and plotted with a custom Matlab script. A U2 Tougaard background was applied to all individual elemental regions.

The Mo 3d region was fit with up to 9 peaks: four split peak Mo 3d pairs (2H MoS_2_, 1T MoS_2_, MoS_2‑*x*_, and/or MoO_3_) and the overlapping S 2s. The binding energy position ranges of the Mo 3d_5/2_ environments are given in Table 1 and the spectra were fit with either “loose” (0.2–3.0 eV) or “sharp” (0.2–2.0 eV) full-width at half-maximum (FWHM) restrictions^31^ (see Supporting Information Note I). Mo 3d_3/2_ components were constrained relative to their respective Mo 3d_5/2_ peak by separation (+3.1 eV), area (66.7%), and FWHM (110%).

**Table 1:** XPS Peak Assignment and Binding Energy Range Fitting Conditions

Name	Material	Assignment	Range (eV)	FWHM Restriction (eV)
**Mo 3d region:**				
POS-A	2H MoS_2_	Mo^4+^ 3d_5/2_	228.9–229.9	0.2–2.0
POS-B	1T MoS_2_	Mo^4+^ 3d_5/2_	228.3–229.0	0.2–2.0
POS-C	MoS_2‑*x*_	Mo^4+^ 3d_5/2_	227.9–228.3	0.2–3.0
POS-D	MoO_3_	Mo^6+^ 3d_5/2_	230.5–235.0	0.2–3.0
S 2s	MoS_2_	S^0/2–^ 2s	223.0–239.2	0.2–3.0
**S 2p region:**				
POS-A	2H MoS_2_/MoS_2‑*x*_	S^2–^ 2p_3/2_	161.8–163.5	0.38–3.0
POS-B	1T MoS_2_	S^2–^ 2p_3/2_	160.5–162.0	= POS-A
S_*x*_	S_8_/S_*x*_	S^0^ 2p_3/2_	163.5–165.0	0.38–2.0
SO_*x*_	SO_*x*_	S^(*x*/2)+^ 2p_3/2_	165.0–170.0	0.20–5.0

In the S 2p region, all MoS_2_ peaks shared a common FWHM (with a loose limit of 0.38–3.0 eV) and the MoS_2_ S 2p_1/2_ peaks were constrained relative to their respective S 2p_3/2_ peak by separation (+1.16 eV), area (50%), and FWHM (100%). The S 2p_3/2_ POS-B peak was further restricted to match the FWHM of the S 2p_3/2_ POS-A peak. Where necessary, multiple SO_*x*_ peaks were included.

All Mo 3d_5/2_ (1, 1, 300), Mo 3d_3/2_ (1, 2, 300), S 2p_3/2_ (1, 1, 300), and S 2p_1/2_ (1, 1, 300) peaks used an asymmetric tail Lorentzian line shape TLA, whereas all other XPS peaks only used an asymmetric Lorentzian line shape LA­(1.53, 243). Throughout all Mo 3d and S 2p figures we have inserted vertical lines at 229.3, 232.4, 162.2, and 163.36 eV for visual reference, which match the as-cast MoS_2_ electrode positions.

## Results & Discussion

### Pristine & As-Cast Sample Surfaces

We elected to study bulk commercial 2H MoS_2_ powder, with an average particle size of ∼6 μm, due to its wide availability and common usage in MoS_2_ lithium-ion mechanism research^21,42,43^ (Table S2). We refer to the commercial MoS_2_ powder as “top-down precursor MoS_2_ powder” following the nomenclature we defined previously^11^ or simply as “MoS_2_ powder”.

The pristine powder was slurry-cast (Figure S2) using 2-methyl-pyrrolidone (NMP) onto a copper current collector (11 μm) to form the as-cast MoS_2_ battery electrode (∼36 μm thick ± 4.6 μm & ∼3.43 mg/cm^2^ ± 0.13 mg/cm^2^), following the standard literature 80:10:10 wt % formulation (Table S2) of MoS_2_/Super P/Polivinylidene fluoride (PVDF). We elected to use a PVDF and NMP formulation due to the large body of MoS_2_ literature already applying this formulation, despite the industry and academic wide shift toward greener formulations such as carboxymethyl cellulose (CMC) and water^44^ or even dry processing without a solvent.^45^ To establish the characterization baseline of the pristine 2H MoS_2_ powder and as-cast battery electrode (Figure S3), detailed characterization of the pristine samples with XPS (Figure 2 and S4), SEM (Figure S5), XRD (Figure S6), and Raman spectroscopy (Figure S7) was carried out and detailed in Supporting Information Note II.

**Figure 2:**
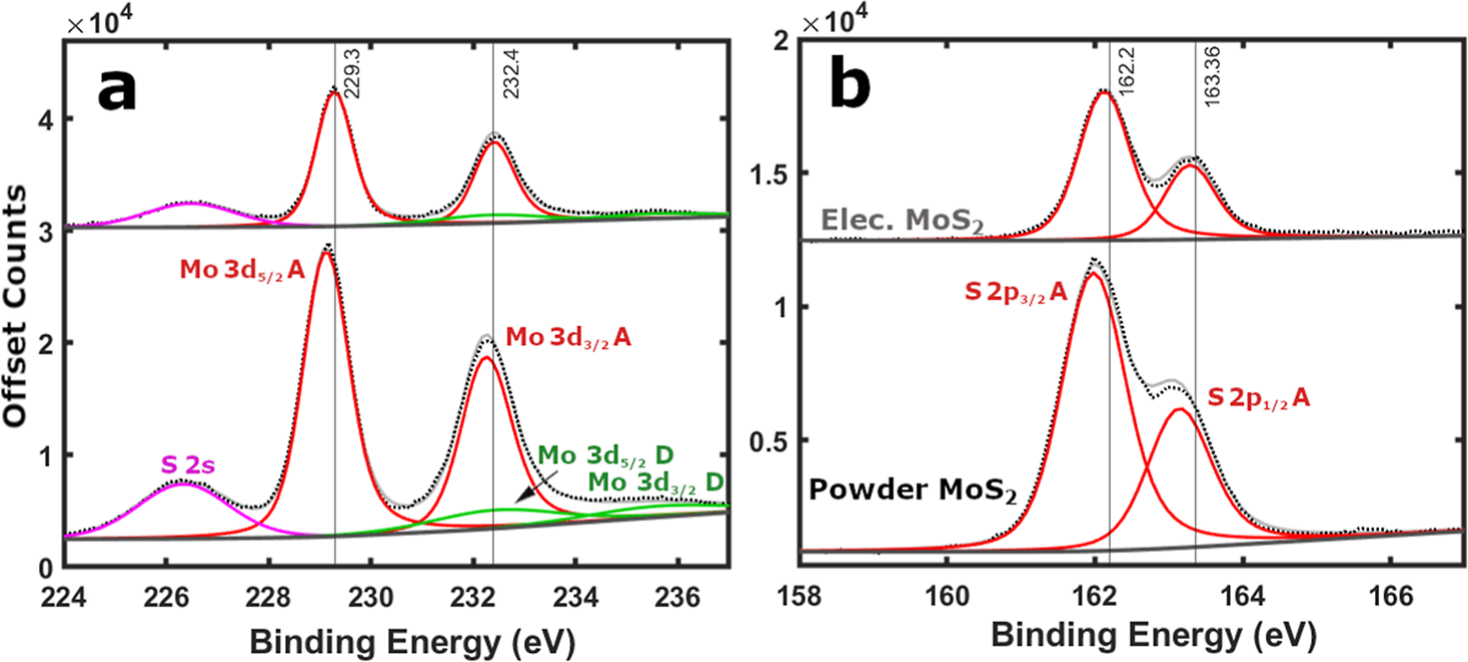
XPS of pristine top-down precursor MoS_2_ powder (bottom) and as-cast battery electrode (top). Black dotted lines represent the raw XPS data, light gray solid lines depict the fitting envelope, and dark gray solid lines display the U2 Tougaard background. Solid colored lines signify peak fitting, such as the Mo 3d split orbit peaks POS-A in red and POS-D in green. (a) Mo 3d and (b) S 2p scan regions. Data reprinted with permission from source.^21^

Due to the multitude of XPS peaks and peak positions discussed within this study, we herein introduce the following nomenclature. The 2H phase top-down precursor MoS_2_ peaks (Mo 3d and S 2p) will be referred to as position POS-A, the 1T phase MoS_2_ (Mo 3d and S 2p) peaks will be denoted as position POS-B, the MoS_2‑*x*_ (Mo 3d) peaks will be designated as position POS-C, and the MoO_3_ (Mo 3d) peaks will be assigned POS-D. Peak positions will be labeled by solely the major split orbital peak (e.g. Mo^4+^ 3d_5/2_ POS-A) with the reduced form (e.g. Mo 3d POS-A), as separation between spin orbit peak pairs is fixed (see Experimental Methods). We apply a four split orbit peak model considering the presence of POS-A, POS-B, POS-C, and POS-D Mo 3d, and POS-A and POS-B S 2p peaks after sample ion bombardment.

Pristine MoS_2_ powder XPS (Figure 2) is defined by clear sharp Mo 3d POS-A (229.1 eV) and S 2p POS-A peaks (161.9 eV), with a peak separation ΔBE ∼67.2 eV. A minor MoO_3_ POS-D contribution can also be found in pristine samples (Figures 2 and S8), and its inclusion is critical for accurate analysis (see Supporting Information Note III). The Mo and S POS-A peaks identify the top-down precursor MoS_2_ powder as 2H phase, dominate the XPS survey spectrum (Figure S9), and contribute to a combined 64.2 at. % of the sample (Figure S10a). Whereas the combined adventitious carbon and oxygen form a smaller fraction of the powder composition (35.8 at. %). XPS of an as-cast MoS_2_ battery electrode (Figure 2) displays POS-A (229.3 eV and 162.2 eV) surface peaks upshifted slightly relative to the pristine powder (229.1 and 161.9 eV), with a conserved peak separation^30,31,36^ (ΔBE ∼67.2 eV).^21^ These features clearly identify the electrode as 2H phase MoS_2_ consistently across multiple locations (Figure S4).

However, the electrode’s survey spectrum (Figure S9) is dominated by the presence of fluorine (F 1s and F KLL) and carbon (C 1s and C KLL) from the PVDF binder and the amorphous Super P conductive additive.^46^ The introduction of these materials leads to carbon (55.9 at. %) and fluorine (20.6 at. %) comprising most of the surface composition (Figure S10b), with MoS_2_ (17.1 at. %) representing a minor fraction of the surface despite the electrode being manufactured as 80:10:10 wt % (MoS_2_/Super P/PVDF). For a well-mixed slurry, the anticipated MoS_2_ atomic percentage is 35 at. % and the experienced deviation is attributed to the lower-density carbon conductive additive and PVDF binder floating to the surface of the electrode during production.^46^

### Model Selection: Top-Down Precursor MoS_2_ Powder Etching (10 s)

Gentle etching of top-down precursor 2H MoS_2_ powder via 10s increments of argon ion bombardments results in a gradual change of the Mo 3d and S 2p regions^30,32^ (Figure 3) that is difficult to observe in the survey spectrum (Figure S11a) as detailed in Supporting Information Note IV. Even for the shortest duration of ion bombardment measured (10s in Figure 3), lower binding energy shoulders begin forming for peaks Mo 3d_5/2_ and Mo 3d_3/2_ (Figure 3a), while at a glance the S 2p region appears to only broaden due to ion bombardment induced disordering^32^ (Figure 3b). The formation of lower binding energy Mo 3d peaks is typically claimed as a partial transition into a mixed 1T/2H phase MoS_2_ ^39–41^ or the creation of MoS_2‑*x*_.^30,31,33–36^ However, synthetic 1T phase MoS_2_ also exhibits clear peak downshifting in the S 2p region,^28,29,47,48^ whereas depth-profiling induced MoS_2‑*x*_ formation results in minimal S 2p alteration.^31,32^

**Figure 3:**
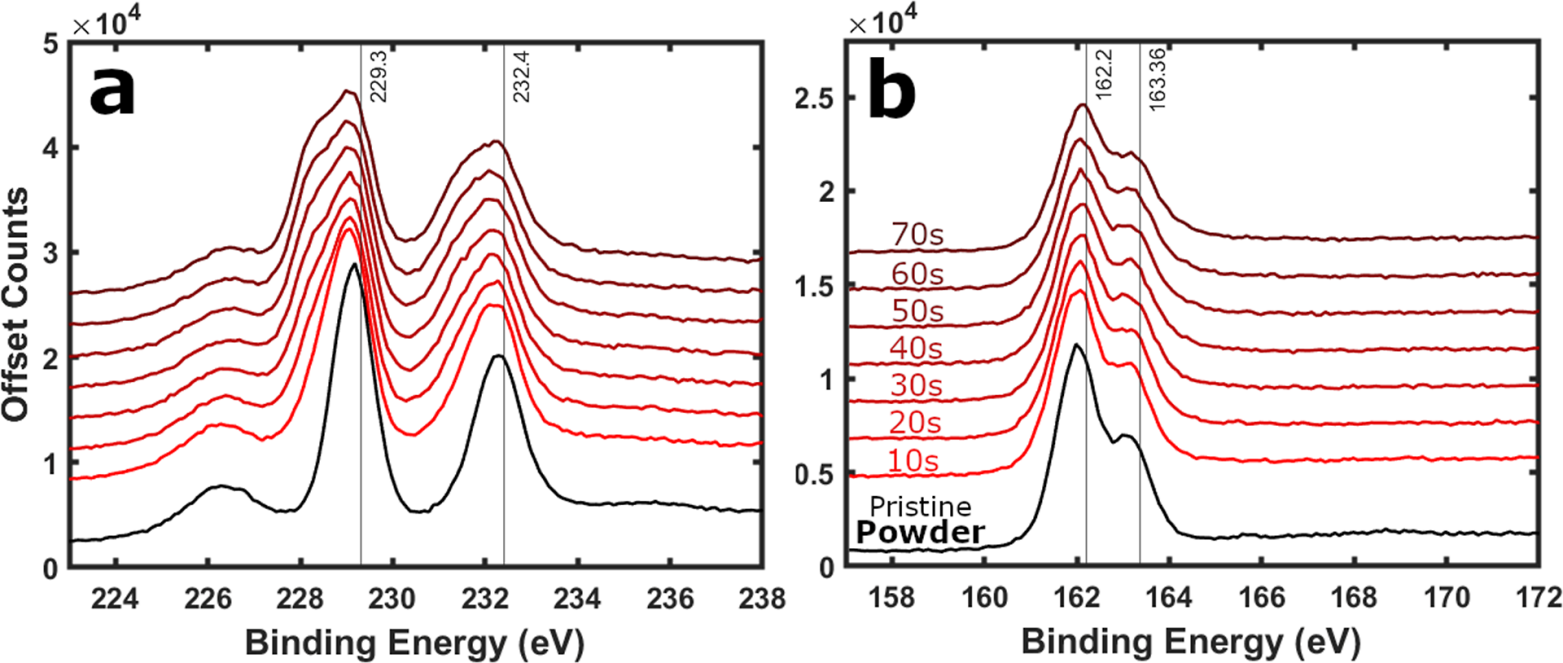
Offset XPS raw data from top-down precursor MoS_2_ powder depth-profiled by Ar^+^ ion 10s depth levels. (a) Mo 3d and (b) S 2p regions.

Therefore, we first opted to fit the Mo 3d and S 2p regions with Mo 3d POS-A, Mo 3d POS-C, and S 2p POS-A peaks (Figure S12) as is established for etching induced MoS_2‑*x*_ formation.^30,32^ The double split peak MoS_2‑*x*_ model yields an overall S/Mo atomic ratio that reduces from 2.20 at the surface to 1.73 after 70s of ion bombardment (Figure S13), as expected from the literature.^30,32^ However, this fitting procedure considers significant broadening of the S 2p POS-A peaks, which have a full-width at half-maximum (FWHM) of 1.01 at the surface and 1.22 after sputtering (Figure S12b). Given the retention of the Mo 3d POS-A shapes after etching it is unusual that only the S 2p peaks broaden. Thus, it is possible that the perceived broadening of the S 2p region (Figure 3b) masks the presence of a second set of S 2p peaks.

Hence, we then attempted to fit the depth-profiled data with Mo 3d POS-A, Mo 3d POS-B, S 2p POS-A, and S 2p POS-B peaks appropriate for 1T MoS_2_ formation^22,40^ (Figure S14). With this approach the Mo 3d region remains identical (Figure S14a), but the introduction of the minor POS-B S 2p peaks to the S 2p region maintain the POS-A S 2p peaks’ FWHM similar to the original surface peaks (Figure S14b). However, the 1T MoS_2_ fitting model expresses an increasing S­(POS-A)/Mo­(POS-A) atomic area peak ratio with a value of 2.7 after 70s (Figure S15) suggesting generation of excess sulfur in the 2H MoS_2_ phase. The formation of excess sulfur strongly contradicts with the literature wide consensus that argon ion bombardment leads to preferential removal of sulfur from MoS_2_.^30,36^

Attempting to justify the high S/Mo atomic ratio, any sulfur liberated from an extremely sulfur deficient 1T MoS_2‑x_ phase could form elemental S_*x*_ (∼164 eV),^31,49^ which we have not observed in significant amounts (Figure S14b). On the other hand, if sulfur rich MoS_3_ is generated Mo 3d peaks would appear at higher binding energies than for 2H MoS_2_, which we have also failed to observe (Figure S14a). Thus, we reject the possibility of any sulfur rich composition being present. Additionally, due to the lack of major 1T (POS-B) peaks in the S 2p region^11,21^ (Figure S15b) to pair with the large Mo 3d POS-B shoulders (Figure S14a), as we have observed previously in lithium-ion battery generated 1T Li_*x*_MoS_2_,^21^ we cannot conclude consistently between the two XPS regions that 1T MoS_2_ is formed in such large amounts.

Therefore, we finally consider the fitting of the depth-profiled powder data with a combined presence of 1T MoS_2_ and MoS_2‑*x*_, with split orbit^36,37,50^ sets of Mo 3d POS-A, Mo 3d POS-B, Mo 3d POS-C, Mo 3d POS-D, S 2p POS-A, and S 2p POS-B peaks (Figure 4), labeled as the four split orbit peak model. In this case, Mo 3d POS-B, Mo 3d POS-C, and S 2p POS-B peaks are uncovered with etching (Figures 4 and S16–S18). Nevertheless, throughout the depth-profiling process the Mo and S POS-A peaks remain dominant (Figure 3). Utilizing the four split orbit peak model addresses both concerns raised previously for the MoS_2‑*x*_ and 1T MoS_2_ double split orbit peak models. With the combined model, both the S 2p POS-A remains similar after etching (Figure S16b) and the S­(POS-A)/Mo­(POS-A + POS-C) atomic ratio decreases with etching (Figure 5), as is anticipated for preferential sulfur removal from a sample.

**Figure 4:**
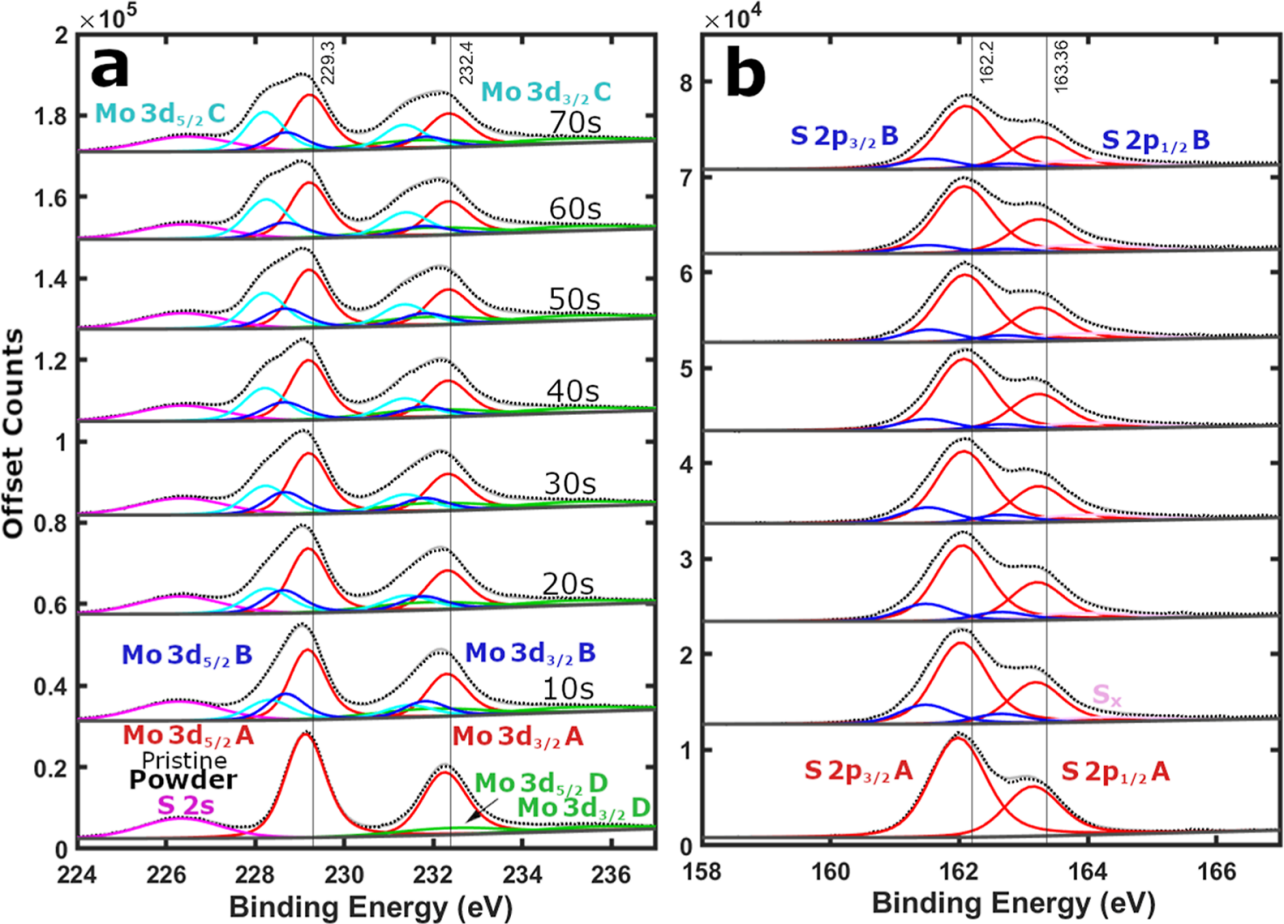
XPS of top-down precursor MoS_2_ powder etched by Ar^+^ ion 10s depth levels. Black dotted lines represent the raw XPS data, light gray solid lines depict the fitting envelope, and dark gray solid lines display the fitting background. Solid colored lines signify peak fitting, such as the Mo 3d split orbit peaks (POS-A red, POS-B dark blue, POS-C cyan, and POS-D green), the S 2p split orbit peaks (POS-A red and POS-B dark blue), the single S 2s peak (magenta), and the elemental sulfur S_*x*_ peak (pink). (a) Mo 3d and (b) S 2p scan regions.

**Figure 5:**
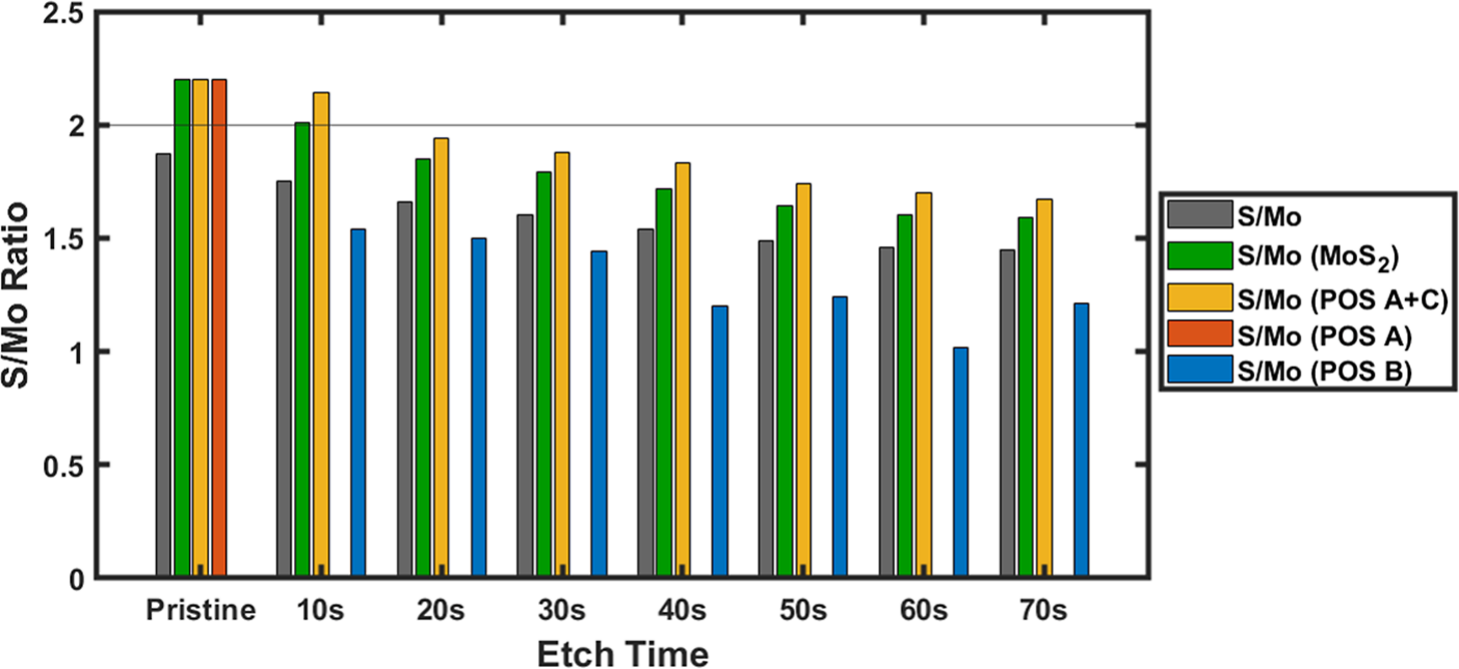
S/Mo XPS atomic ratios from fitting top-down precursor MoS_2_ powder (Figure 4) depth-profiled by Ar^+^ ion (10s depth levels) with a four split orbit peak model. S/Mo (gray) denotes the ratio of all Mo and S present in the sample including Mo 3d MoO_3_, MoS_2_ (green) represents all Mo and S species collectively across all MoS_2_ phases, and POS-A (red), POS-B (blue-without S 2s), and POS A + C (yellow) represent Mo and S species detected in the 2H MoS_2_, 1T MoS_2_, and MoS_2‑*x*_ phases, respectively.

The Mo POS-A and POS-C peaks can be grouped in this manner for the calculation of the S/Mo atomic ratio, as their corresponding sulfur pair (POS-A) has not experienced any change in atomic environment.^32^ Sulfur atoms in 2H MoS_2_ and MoS_2‑*x*_ are surrounded by molybdenum atoms and as sulfur is preferentially removed the molybdenum coordination around the sulfur atoms still present in the material lattice remains unchanged. Hence, the binding energy of sulfur (POS-A) in both 2H MoS_2_ and MoS_2‑*x*_ remains identical despite ion bombardment. On the other hand, as sulfur is removed molybdenum atoms in MoS_2‑*x*_ experience a stark change in their surroundings relative to 2H MoS_2_. Therefore, with depth-profiling the molybdenum MoS_2‑*x*_ POS-C binding energy is significantly downshifted relative to 2H MoS_2_. Further justification of the S­(POS-A)/Mo­(POS-A + POS-C) atomic ratio after depth profiling can be found in Supporting Information Note V (Figure S18). Henceforth, we elected to apply the four split orbit peak model to all MoS_2_ samples that involve depth-profiling throughout this study.

### Top-Down Precursor MoS_2_ Powder Etching

Analyzing the depth-profiled MoS_2_ powder sample in detail, it is established that after only 10s of etching the contribution of 1T MoS_2_ in the sample is at its highest (Figures 4 and S19a) while the presence of MoS_2‑*x*_ is lowest. Confirming that low dose argon etching can be used to synthesize 1T MoS_2_,^40,41^ albeit with low purity (18.7% Mo 3d). For longer time lengths of ion bombardment the Mo 3d composition of MoS_2‑*x*_ grows while the 1T MoS_2_ percentage decreases and the MoO_3_ fraction remains constant (Figure S19a). As the sulfur deficient MoS_2‑*x*_ S 2p percentage increases, a small amorphous elemental sulfur S_*x*_ (163.9 eV)^31,49^ fraction grows (Figures 4b and S19b). However, there is a mismatch between the two rates, as a notable amount of released sulfur is lost to the dynamic vacuum of the XPS. Despite a significant initial decrease, 2H MoS_2_ remains dominant for the whole depth-profiling duration (Figure S19).

Interestingly, the binding energy positions of the O 1s and C 1s peaks (see Supporting Information Note VI for fitting details) in the MoS_2_ powder are not affected by depth-profiling. Since their presence is largely due to surface adsorption, etching does not cause a shift in their positions but leads to a drastic decrease in peak intensity as the adsorbents are removed from the surface (Figures S20 and S21). Therefore, with etching the percentage of Mo and S in the powder increases as adventitious carbon and adsorbed oxygen are removed from the surface (Figures S20 and S21); however, as etching proceeds the MoS_2_ composition fraction increase slows down, tending toward 79.0 at. % MoS_2_ and 21.0 at. % carbon and oxygen after 70s.

After 70s of bombardment the POS-A peaks (229.2 eV and 162.1 eV in Figure 4) are fractionally upshifted compared to the pristine surface (229.1 eV and 161.9 eV). Nonetheless, the separation between the POS-A peaks remains unchanged (ΔBE ∼67.1 eV). At the same time, the Mo 3d POS-B, Mo 3d POS-C, and S 2p POS-B peaks, are located at 228.7 eV, 228.2 eV, and 161.5 eV respectively (70s in Figure 4). The Mo 3d POS-B and POS-C peaks are separated by 0.5 and 1.0 eV, respectively, relative to the conserved Mo 3d POS-A peak (Figure 4a). Thus, hinting at a potential explanation for the discrepancy we previously uncovered in literature 1T MoS_2_ XPS reporting.^11^

Although, sulfur sputtering^39–41^ is believed to enable the gliding of the molecular plane which causes the 2H to 1T MoS_2_ phase transition,^41^ the S/Mo atomic ratio of the POS-B peaks in the powder sample is in the range of 2.0–2.4 when the S 2s peak is included in the ratio calculation. A POS-B S/Mo ratio above 2.0 suggests that the excess sulfur induces phase transition. However, if the S 2s peak is excluded from the POS-B S/Mo atomic ratio calculation, due to the size similarity of the Mo POS-B and the S 2s peaks (Figure 4a), the POS-B S/Mo atomic ratio is noticeably lower (1.0–1.5 in Figure 5–blue). Thus, confirming the impact of sulfur deficiency on argon ion bombardment 1T MoS_2_ formation.

To confirm the previous powder depth-profiling observations (Figure 4), investigate the effects of higher dose ion bombardment, and check the stability of the altered 1T MoS_2_/MoS_2‑x_ under vacuum conditions, a second batch of top-down precursor 2H MoS_2_ powder was etched under a 10-fold (100s) increase in etch time per layer. After completing 200s of depth-profiling, a final scan was conducted after approximately 1 h of rest (Figures 6 and S22) to verify if the change in the Mo 3d and S 2p regions is temporary and/or reversible.

**Figure 6:**
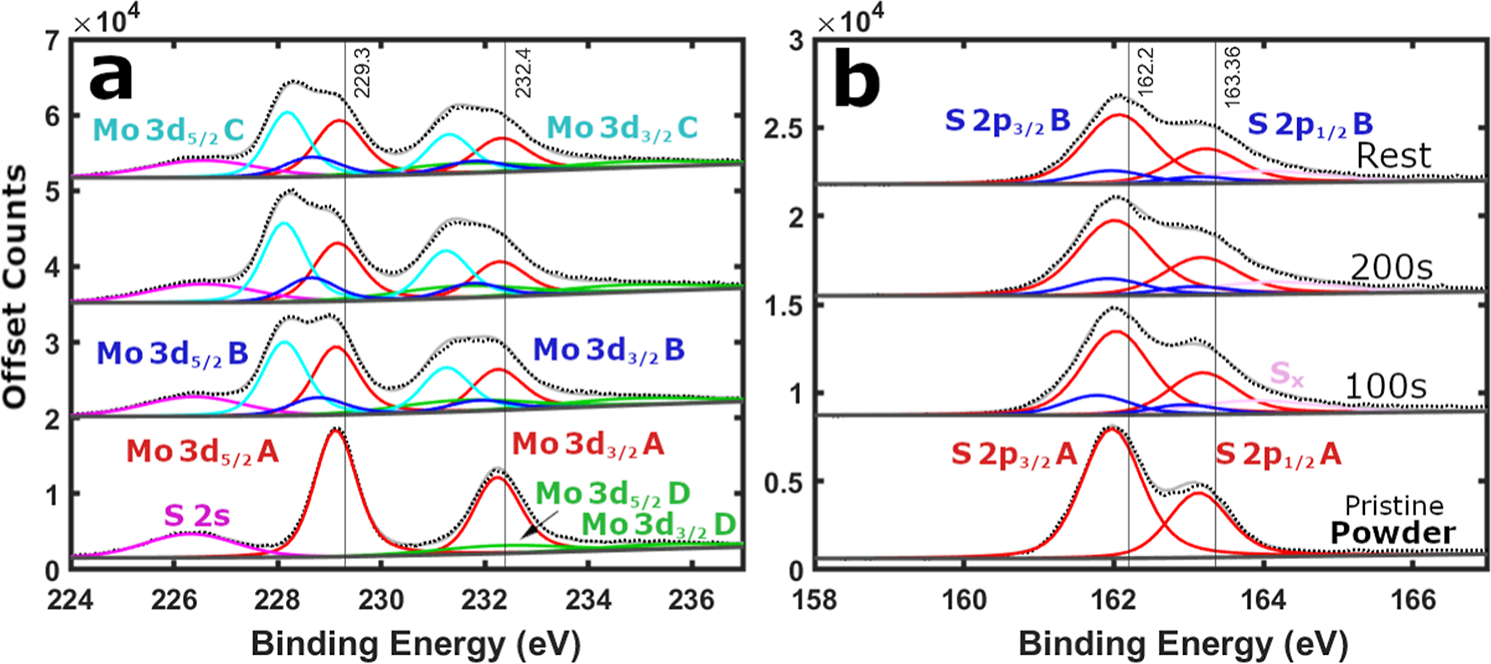
XPS of top-down precursor MoS_2_ powder etched by Ar^+^ ion 100s depth levels, followed by a 1 h rest before a confirmation scan. Black dotted lines represent the raw XPS data, light gray solid lines depict the fitting envelope, and dark gray solid lines display the fitting background. Solid colored lines signify peak fitting. (a) Mo 3d and (b) S 2p scan regions.

The second sample displays clear 2H phase MoS_2_ POS-A (229.1 eV and 161.9 eV) peaks on the surface and forms noticeable Mo 3d POS-C (228.1 eV) and minor POS-B (228.6 eV and 161.9 eV) peaks with etching (Figure 6a). After 100s the Mo 3d POS-C peaks are more prominent than the conserved POS-A peaks (229.1 eV), while the 1T MoS_2_ contribution remains minimal (<14% Mo 3d in Figure S23a). Again, the S 2p region experiences the formation of a broad minor S_*x*_ peak (164.1 eV) and the overall S/Mo atomic ratio (Figure S24-green) decreases from 2.4 at the surface to 1.6 after 200s. Similarly, the POS-B S/Mo ratio is 1.6 after 200s. There are no noticeable differences in the Mo 3d and S 2p peaks after 1 h of rest in the XPS vacuum environment, highlighting that the induced changes are permanent under the spectrometer high-vacuum conditions (Figures 6 and S22).

Therefore, the combination of a decrease in the S/Mo atomic ratio of MoS_2_ species following etching (Figure S24), the formation of a minor broad elemental sulfur peak^49^ (S_*x*_ in Figures 4b and 6b), and the presence of Mo 3d and S 2p peaks with positions POS-A, POS-B, and POS-C (Figures 4 and 6) sets an XPS fingerprint for altered MoS_2_ powders. Whereby, 2H MoS_2_ is partially conserved (40.9% Mo 3d), a sulfur reduced MoS_2‑*x*_ composition forms (28.1% Mo 3d), and a minor amount of 1T MoS_2_ (13.9% Mo 3d in Figure S19a after 70s) is produced. Similarly, for the S 2p percentage calculation 2H MoS_2_/MoS_2‑*x*_ remains dominant (79.1% S 2p) and 1T MoS_2_ comprises a small amount of the sample (11.6% S 2p after 70s in Figure S19b). Therefore, considering that the 1T MoS_2_ phase percentage decreases with etching in MoS_2_ powders (Figure S19), a 1T MoS_2_ threshold value can be established (Mo 3d < 19% & S 2p < 19%). For argon etched MoS_2_ powder samples a Mo 3d or S 2p phase percentage beyond the threshold signifies that the pristine sample contains 1T MoS_2_ before depth-profiling that originates from material synthesis.

### Battery MoS_2_ Electrode Etching

To establish any differences between the MoS_2_ powder and battery electrode forms, we depth-profiled NMP slurry-cast MoS_2_ electrodes with argon ions for 100s per etch level and applied the four split orbit peak model. As expected, etching results in the alteration of the Mo 3d and S 2p regions (Figures 7 and S25–S26), with the generation of lower binding energy Mo 3d shoulders and S 2p broadening. However, within 100s of etching the Mo 3d POS-C peaks (228.1 eV) become dominant, surpassing the conserved POS-A peaks (229.2 eV and 162.0 eV), while the POS-B (228.7 eV and 161.9 eV) peaks remain marginal (Figure 7a). Meanwhile, the amorphous sulfur peak S_*x*_ (164.0 eV) is more prominent than in the powder form and the S/Mo atomic ratio decreases more sharply from 2.5 at the electrode surface to 1.1 after 200s (Figure S27 – green). Therefore, preferential sulfur removal is significantly stronger in electrode samples than in powders (S/Mo ∼ 1.6).

**Figure 7:**
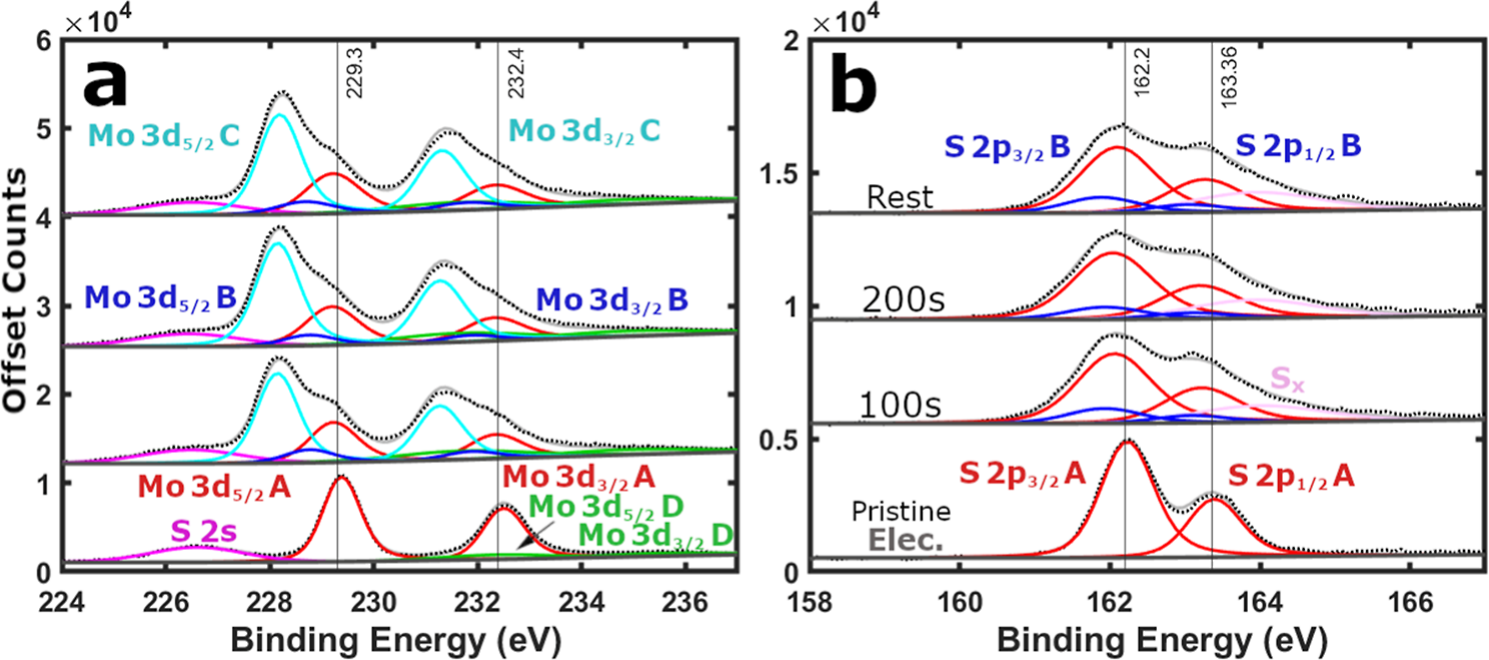
XPS of top-down precursor MoS_2_ battery electrode (∼36 μm) etched by Ar^+^ 100s depth levels, followed by a 1h rest before a confirmation scan. Black dotted lines represent the raw XPS data, light gray solid lines depict the fitting envelope, and dark gray solid lines display the fitting background. Solid colored lines signify peak fitting. (a) Mo 3d and (b) S 2p scan regions.

Correspondingly, the composition of the electrode is significantly more altered than in the powder (Figure S28). After 100s of depth-profiling, from the Mo 3d percentage calculation (Figure S28a) less 2H MoS_2_ is conserved (25.9% Mo 3d), more MoS_2‑*x*_ is formed (49.9% Mo 3d), and less 1T phase MoS_2_ is produced (8.7% Mo 3d). Similarly, the S 2p percentage calculation (Figure S28b) showcases mostly 2H MoS_2_/MoS_2‑*x*_ (71.3% S 2p) and significantly less 1T MoS_2_ (13.4% S 2p). These trends hold true even after an hour of rest in the XPS vacuum environment (Figure 6). However, for the battery electrode, the F 1s, O 1s, and C 1s regions (FOC) should also be considered (see Supporting Information Note VI), as the as-cast electrode surface is mostly made up of carbon (58.3 at. %) and fluorine (18.0 at. %) rather than MoS_2_ (16.4 at. % in Figure S10b), and their presence persists with etching (Figure S29). As the FOC species show no presence in the Mo 3d and S 2p analysis (Figure 6) they can be treated as unrelated.

The F 1s (687.3 eV) and O 1s (531.7 eV) features can be fitted with single symmetric peaks (Figure S30a,b), representing an organic fluoride bond (C–F) in the PVDF binder and surface adsorbed oxygen, respectively. With depth-profiling, the O 1s peak is eliminated (Figure S30b) alongside the O KLL Auger peak (976 eV) (Figure S11b), validating the removal of adsorbed oxygen by ion bombardment (Figure S29). The C 1s region is more complex due to the existence of multiple carbon peaks (Figure S30c). However, etching leads to the reduction of most adventitious carbon peaks leaving behind only the C–C/C–H (284.4 eV) and C–O/C–F (285.6 eV) contributions.

Therefore, the XPS fingerprint for depth-profiled MoS_2_ electrodes includes a sharper decrease in the S/Mo ratio (∼1.1), a stronger formation of elemental sulfur S_*x*_, and the presence of Mo 3d and S 2p POS-A, POS-B, and POS-C peaks. Whereby, the 1T MoS_2_ bombardment induced threshold (Mo 3d < 10% & S 2p < 13% in Figure S28) is lower than for the powder samples (<19%).

### Analysis of Ex Situ MoS_2_ LIB Electrodes

Having established the changes induced by argon bombardment on as-cast electrodes, the four split orbit peak model can be applied to the MoS_2_ XPS depth-profiling data from our recent study on lithiated ex situ LIB electrodes^21^ (see Supporting Information Note VII). MoS_2_ electrodes undergoing initial lithiation (discharge-**D1**) and delithiation (charge-**C1**) in coin cells experience tricolored ring patterns (Figure 8) associated with an uneven lithiation gradient^21,51^ (Figure S31). We introduce the following nomenclature^21^ for the colored rings (e.g. AX2 in Figure 8), where the first letter refers to the depth of discharge (AX-D1 0.80 V, BX-D1 0.01 V, or WX-C1 3.00 V), and the number denotes the ring position (1-outermost ring, 2-middle ring, and 3-center).

**Figure 8:**
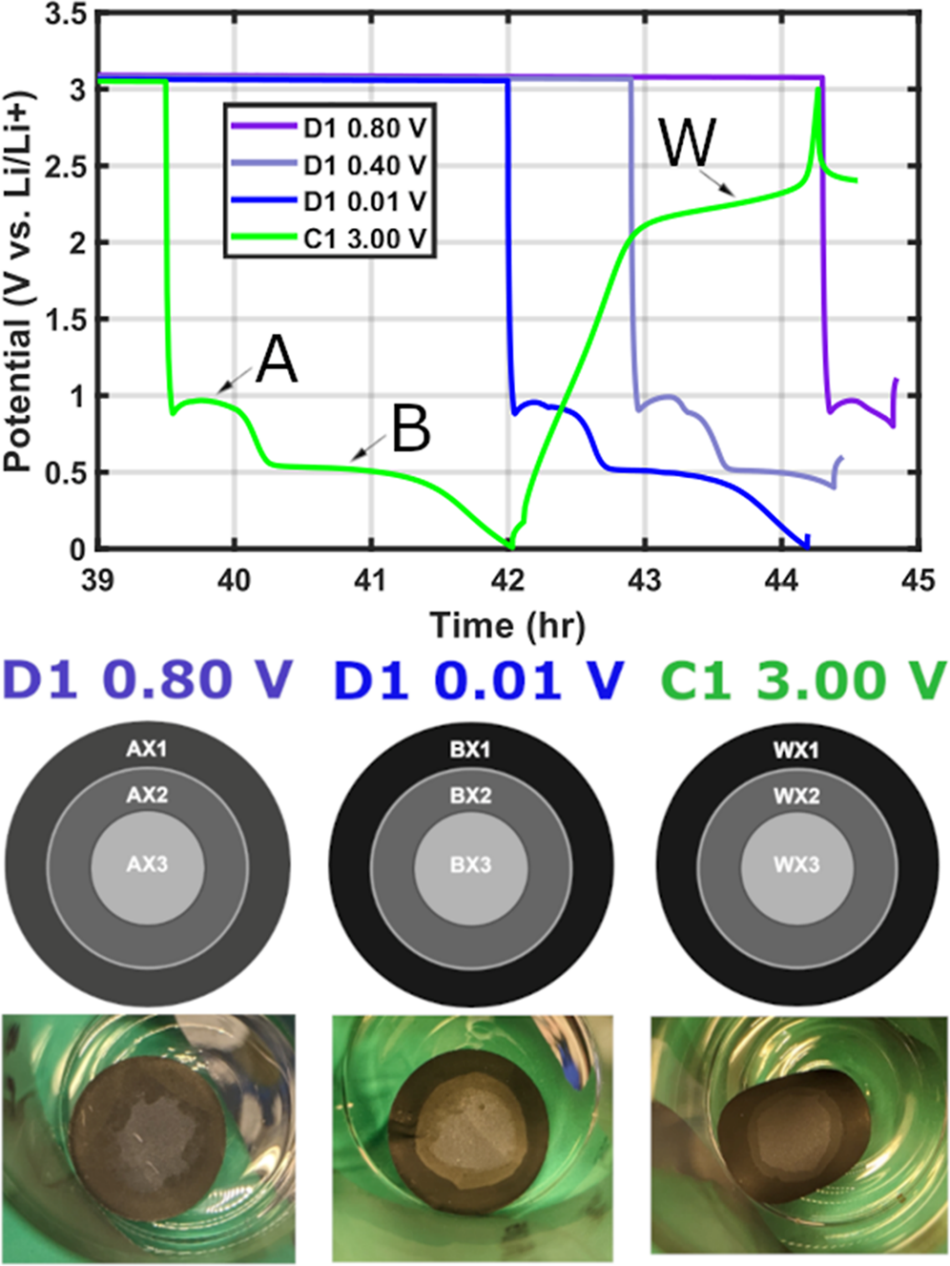
Ex situ LIB MoS_2_ electrode nomenclature used throughout the study based on the plateaus reached during the first lithiation/delithiation cycle. Figure adapted with permission from source.^21^

Prior, without a methodology to differentiate between depth-profiling induced alteration and MoS_2_ lithiation (Figures S32 and S33), our XPS analysis of the ex situ electrode rings was restricted to the electrode surface (Figure S34). Like the as-cast electrode (Figure S32a,b) XPS depth profiling of the lithiated ex situ rings results in the significant downshift of the Mo 3d peaks and broadening of the S 2p region (Figures S32 and S33). Previously, by combining information across SEM, XRD, Raman (Figure S35), and surface XPS^21^ (Figures S34 and S36) the ex situ electrode centers (AX3, BX3, & WX3) were found to be electrochemically inactive during discharge and remain as 2H MoS_2_, the middle rings (AX2, BX2, & WX2) were partially active and formed heterogeneous 1*T*/2H MoS_2_ (228.4 eV and 161.5 eV; 229.7 eV and 162.2 eV in Figures S37–S39), and the outermost rings (AX1, BX1, & WX1) were most active and exhibited 1T MoS_2_ in regions AX1 and WX1. Each ex situ electrode ring was measured three times for consistency (Figures S37–S39) and despite minor local variations (Figures 9a,b and S40) the stark overall differences between rings are clearly identifiable (Figure S36).

**Figure 9:**
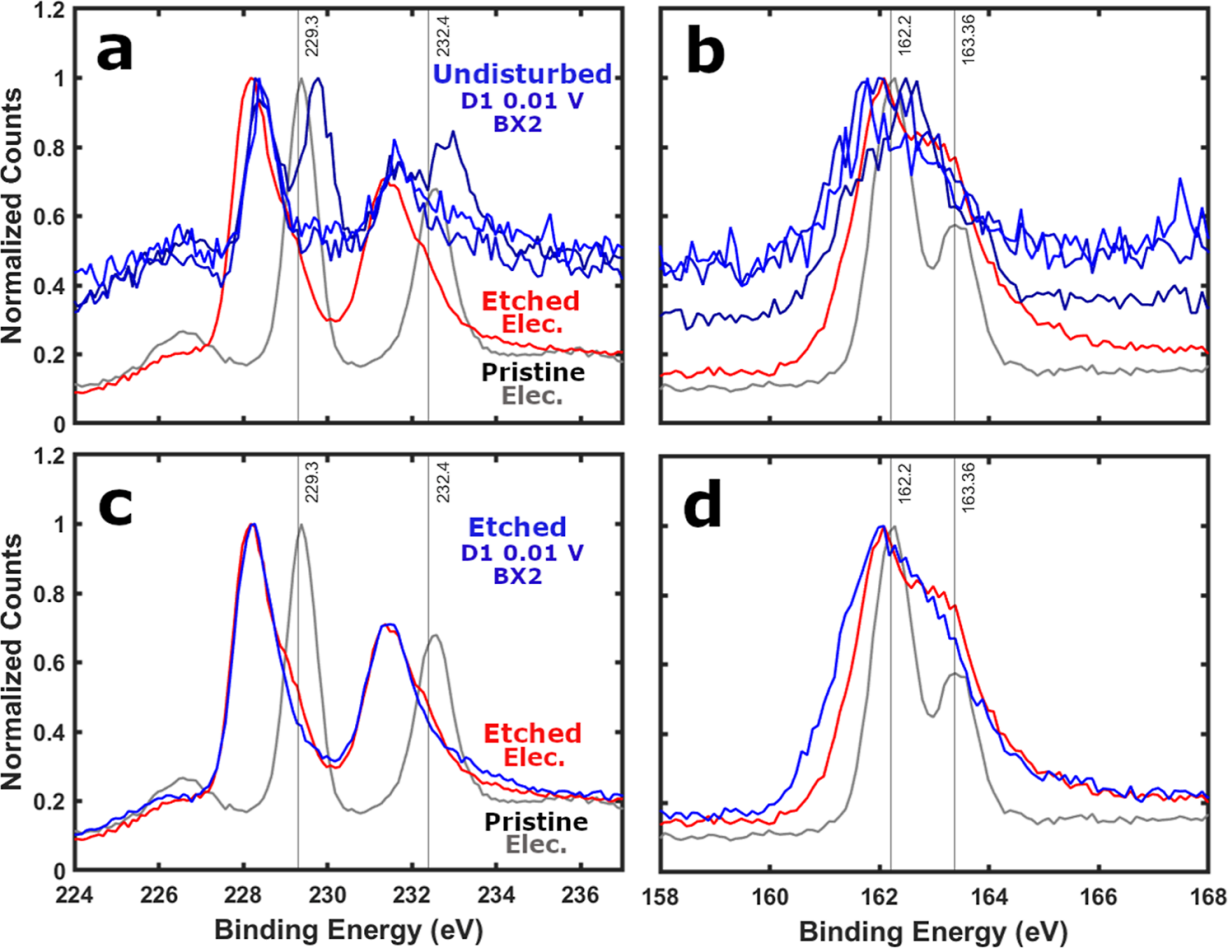
XPS normalized raw data from ion bombarded (200 s) as-cast electrode and (a,b) undisturbed ex situ air-free surface of BX2 or (c,d) ion bombarded (200s) ex situ air-free BX2 electrode segment.

Comparing the raw XPS data from the ion bombardment altered electrode (Figure 7) and the undisturbed surface of the lithiated ex situ LIB MoS_2_ electrodes (AX2, BX2, & WX2), it is evident how subtle the differences in the Mo 3d and S 2p peaks can be between 1T MoS_2_ and MoS_2‑*x*_ (Figures 9a,b and S40). Without additional manufacturing or synthesis information and further material characterization^21^ to aid the fitting process the task becomes extremely difficult. Utilizing the four split orbit peak model for BX2 (Figure S41), the differences between 1T MoS_2_ and MoS_2‑*x*_ can be observed most clearly in the S 2p region (Figure S41b–d).

Despite the low atomic percentages of molybdenum (<1.05 at. %) and sulfur on the ex situ surfaces (Figure S42), the electrode composition being governed by SEI species (fluorine, carbon, oxygen, and lithium in Figure S42) even with depth (Figure S43), and the presence of copper contamination (see Supporting Information Note VIII; Figures S44–S46), depth-profiling reveals the presence of multiphase MoS_2_ states of noticeable intensity below all ex situ surfaces (Figure S47–S49). However, simply fitting the etched ex situ rings with Mo 3d POS-A, POS-B, POS-C, and POS-D states (Figure S47), does not clarify whether the lithiated 1T phase MoS_2_ like that present on the surface (Figures S34 and S37–S39) exists throughout the ex situ electrodes (Figure 9c,d and S50) or the samples are just altered by ion bombardment like in the as-cast electrode (Figure 7).

Therefore, a further indicator is required to differentiate whether 1T MoS_2_ is inherently present in the samples or is solely created by argon bombardment. Utilizing the data gathered bydepth-profiling the as-cast electrode (Figure 7), the lithiated ex situ samples can be re-evaluated against a reference sample with clear depth-profiling induced 1T MoS_2_ threshold values (S/Mo ∼ 1.1, Mo 3d < 10%, & S 2p < 13%). Unfortunately, neither the use of S/Mo atomic ratios (Figures S51 and S52) nor the molybdenum phase percentages (Figures S53 and S54) allow for accurate identification of the phase present prior to ion bombardment, due to large variability in the S/Mo ex situ atomic ratios with etching and the inability of the Mo 1T % (Table S3) to withstand internal threshold validation (see Supporting Information Note IX for details). However, the sulfur phase percentage analysis allows for phase induction of the undisturbed material (Figure 10), as in the S 2p region only the POS-B peaks downshift following ion bombardment (Figure 7b).

**Figure 10:**
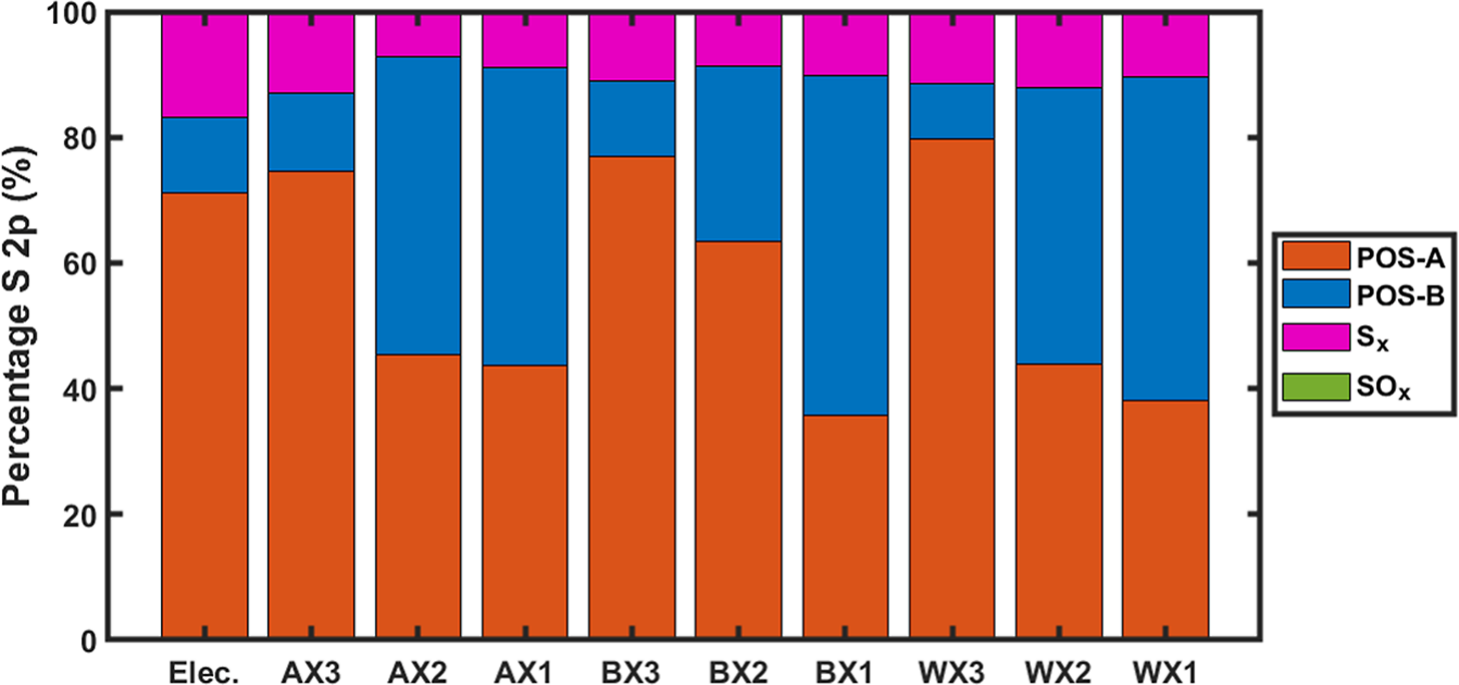
S 2p percentage after etch. Depth-profiling of 200s for the as-cast electrode and 180s for all ex situ LIB MoS_2_ electrode segments.

Relying on the sulfur phase percentage (Figure 10), the inactive central ex situ rings have the lowest 1T percentage after etching (∼11.1% S 2p), closely matching the as-cast electrode after depth-profiling (11.8% S 2p), confirming the central rings’ (AX3, BX3, & WX3) electrochemical inactivity and elucidating the source of their 1T phase MoS_2_ as solely generated by ion bombardment. On the other hand, the middle (AX2, BX2, & WX2) and outer (AX1, BX1, & WX1) rings unambiguously have significantly higher 1T MoS_2_ S 2p percentages following etching, 300%–400% higher than their respective inactive central rings (Table S4).

Therefore, the S 2p region 1T percentage analysis verifies that MoS_2_ electrode lithiation occurs in depth within the electrochemically active middle (AX2, BX2, & WX2) and outer rings (AX1, BX1, & WX1), expanding our previous surface lithiation study (SEM, XRD, Raman, and XPS). Nevertheless, the degree of 1T MoS_2_ lithiation (Li_*x*_MoS_2_) detected in depth (Table S4) is lower than on the electrode surface (Table S5) that is directly exposed to the reactive electrolyte, in all areas except for the outermost AX1, BX1, and WX1 regions. This is especially the case in BX1 (0.01 V), where the surface was covered by a gel-like layer^21^ whose partial removal by depth-profiling allows for XPS detection of significant amounts of 1T MoS_2_ underneath the surface.

## Conclusion

With MoS_2_ energy application research focusing on modifying 2H MoS_2_ or growing 1T MoS_2_ nanosheets via bottom-up approaches and understanding their behavior in energy devices, it is increasingly crucial to deepen key MoS_2_ characterization technique knowledge. Thus, enabling the distinction between MoS_2_ phases, byproducts, and characterization specific alteration like argon ion bombardment induced MoS_2‑*x*_. Especially in lithium-ion battery electrodes, where localized composition XPS studies are exceptionally important as the materials formed during lithiation are amorphous and covered by a thick electrolyte degradation SEI layer.

We have thus combined the 1T MoS_2_ formation and MoS_2‑*x*_ alteration phenomena into a four split orbit peak XPS model (POS-A, POS-B, POS-C, and POS-D) to evaluate depth-profiling of pristine MoS_2_ powder and as-cast battery electrodes. Argon ion etching of pristine samples results in preferential sulfur removal thus forming MoS_2‑*x*_ (228.1 eV) and smaller amounts of 1T phase MoS_2_ (228.7 eV). Sample alteration occurs more drastically in as-cast battery electrodes (S/Mo ∼ 1.1) than in powder samples (S/Mo ∼ 1.6); however, the percentage of 1T MoS_2_ formed by depth-profilingrelatively low thus providing a clear threshold value for ion induced alteration (<13% S 2p) and 1T sample synthesis (>13% S 2p).

Therefore, we were able to evaluate lithiation of ex situ MoS_2_ battery electrodes beyond the surface layer, clearly detecting amorphous 1T MoS_2_ content (>40% S 2p) in the bulk beyond the as-cast electrode threshold (13% S 2p), thus establishing that lithiation occurs in depth in MoS_2_ electrodes and that 1T MoS_2_ exists in the bulk even after a deep discharge (0.01 V).

We thus recommend the following fitting process and calculations for XPS depth-profiling of MoS_2_ samples:

- Fitting a four split orbit peak model (POS-A, POS-B, POS-C, and POS-D) after Ar^+^ ion-bombardment;
- Evaluating the S/Mo atomic ratios for the POS-A, POS-B, and POS­(A + C) peaks;
- Calculating the Mo 3d and S 2p percentages of each phase, especially 1T MoS_2_ and contrasting them against the ion induced threshold value (<13% S 2p for as-cast electrodes) or an internal reference.

## Supplementary Material



## Notes

Process schematics, literature data tables, pristine material characterization (SEM, XRD, and Raman), XPS survey and surface point scans, XPS fitting model development, S/Mo ratios, and ex situ electrodes XPS (PDF)
