# Supplementary material for: XPS Peak-Fitting of 2H MoS2, 1T MoS2, and MoS2‑X Nanosheets in MoS2 Powders and Battery Electrodes After Ar+ Ion Depth-Profiling
Source: ACS Appl Nano Mater. 2026 Jan 6;9(2):1183–94. doi: 10.1021/acsanm.5c04608 (PMC12813980; doi:10.1021/acsanm.5c04608)
Supplement: Supplementary file 1 [file an5c04608_si_001.pdf]

Supporting Information

Title: XPS Peak-Fitting of 2H MoS_2, 1T MoS_2, and MoS_{2-x} Nanosheets in MoS_2 Powders and Battery Electrodes after Ar^+ Ion Depth-Profiling

Authors: Alexandar D. Marinov[(1,2)], Adam J. Clancy[(3)], Christopher A. Howard[(4)], Patrick L. Cullen[(5)] *

1. Electrochemical Innovations Laboratory (EIL), Department of Chemical Engineering, University College London, WC1E 6BT, London, UK
2. CIC energiGUNE, Basque Research and Technology Alliance (BRTA); Alava Technology Park, Albert Einstein 48, 01510 Vitoria-Gasteiz, Spain
3. Department of Chemistry, University College London, WC1E 6BT, London, UK
4. Department of Physics & Astronomy, University College London, WC1E 6BT, London, UK
5. School of Engineering and Materials Science, Queen Mary University, E1 4NS, London, UK

* Corresponding author(s)
Email: p.cullen@qmul.ac.uk

Note I: MoS_2 XPS Peak Fitting FWHM selection

For Mo 3d, previous studies have reported a maximum FWHM broadening up to 1.6 eV[1]. Due to the large number of scans (x55) and corresponding ~ 440 Mo 3d peaks to fit within this study, we opted to use a computational minimization function approach in CasaXPS to ensure consistency across all sample fits. Due to the mathematical nature of this endeavor, we expanded the FWHM limit from 1.6 eV to allow for the identification of any further peak broadening. Thus, we set up FWHM limits of 2.0 eV for POS-A and POS-B (sharp), and 3.0 eV for POS-C (loose).

Despite the wide range considered for POS-B and POS-C, their Mo $3d_{5/2}$ peaks never go beyond a FWHM of 1.47 eV and 1.17 eV, respectively. Similarly, the POS-A surface scans for all samples also never exceed the 1.6 eV limit, but with etching some *ex situ* electrode locations experience FWHM values between 1.6 eV – 2.0 eV for POS-A as more severe broadening occurs.

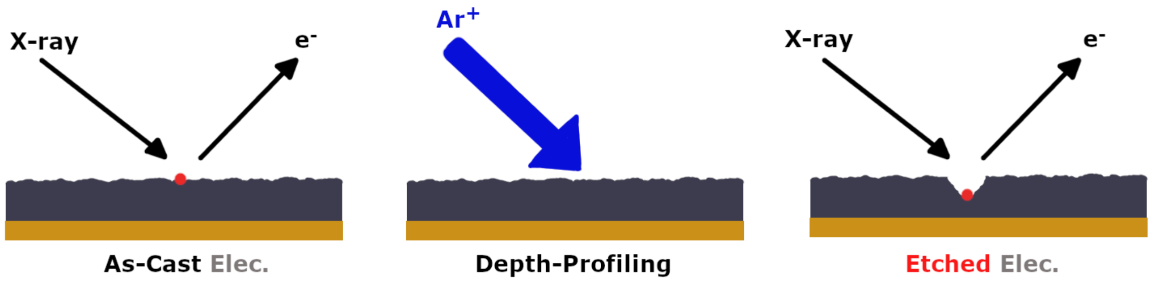

Figure S1. Schematic of the Ar+ ion bombardment depth-profiling (etching) process carried out on MoS_2 electrodes within the XPS spectrometer. The red dot denotes the location where the XPS technique is probing before and after depth profiling.

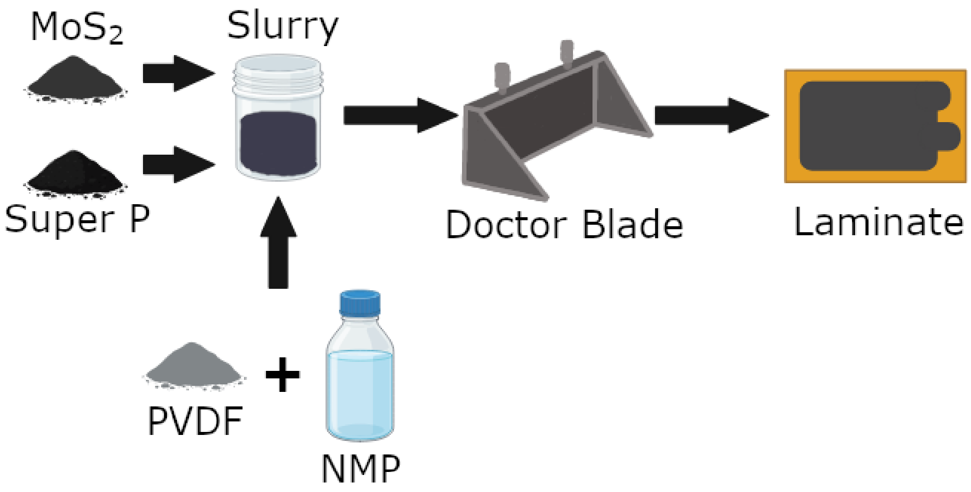

Figure S2. Schematic of a laboratory battery electrode fabrication process. MoS_2 powder, Super P conductive additive powder, and a PVDF/NMP binder mixture were used to formulate the slurry. The slurry was deposited onto a copper current collector using a doctor blade and an automatic coater machine. The formed laminate was dried on a hot plate at 60°C.

Table S1. XPS spectrometer instrument & sampling conditions.

Setting/Conditions	Value
Instrument	K-Alpha ThermoFisher Scientific
Source	Al K_α
Monochromator	Yes
X-ray Wavelength	1486.68 eV
Operating Voltage	12 kV
Operating Current	6 mA
Operating Power	72 W
Beam Size	400 µm × 400 µm
Take-off angle	30°
Flood Gun Source	Ar
Flood Gun Current	100 µA
Flood Gun Energy	1 eV
XPS Chamber Vacuum	2.5E-7 mbar
Survey Spectra No. Scans	5
Survey Spectra Pass Energy	200 eV
Survey Spectra Step Increments	1.0 eV
Elemental Spectra No. Scans	10
Elemental Spectra Pass Energy	50 eV
Elemental Spectra Step Increments	0.1 eV
Depth Profiling Ion Source	EX06 (Ar^+)
Depth Profiling Current	10 µA
Depth Profiling Energy	3 keV
Depth Profiling Raster Width	2 µm
Depth Profiling Ion Source Angle	30°
Depth Profiling Sputter Rate	0.6756 nm/s (Ta_2O_5)

Table S2. MoS_2 and MoS_2/C composite literature electrode materials and composition. NS stands for nanosheets, PVDF is polivinylidene fluoride, CMC is carboxymethyl cellulose, C is carbon, CNT is carbon nanotube, and PANI is polyaniline.

Active Material	Average Particle Size (μm)	Composition (Active:Conductive :Binder) (w:w:w)	Binder: Conductive Additive - Substrate	Ref.
Commercial MoS_2	-	80:10:10	PVDF:carbon black - copper	2
Commercial MoS_2	-	80:10:10	PVDF:carbon black - copper	3
Commercial MoS_2	-	80:10:10	Polytetrafluoroethylene: carbon black - copper	4
Commercial MoS_2	1.5	80:10:10	CMC:Super P -	5
Commercial MoS_2	6.2	80:10:10	polyamide-imide:ketjen black - copper	6
MoS_2 NS	0.6 – 0.9	80:10:10	PVDF:acetylene black - copper	7
MoS_2 NS	6	80:10:10	CMC:carbon black - copper	8
MoS_2/C	-	80:10:10	PVDF:Super P - copper	9
MoS_2/C/CNT-8	0.2	80:10:10	PVDF:Super P - copper	10
MoS_2/CNT	-	80:10:10	CMC:Super P - copper	11
MoS_2/Graphene/ CNT	0.5 – 1	80:10:10	PVDF:acetylene black - copper	12
MoS_2/CNT	-	80:10:10	PVDF:Super P - copper	13
C/MoS_2/CNT	0.1	80:10:10	CMC:acetylene black - copper	14
MoS_2/Graphene	-	80:10:10	PVDF:acetylene black - copper	15
MoS_2/PANI	1 – 10	80:10:10	PVDF:carbon black - copper	16
MoS_2/PANI	0.3 – 0.7	80:10:10	PVDF:carbon black - copper	17
MoS_2 Spheres	0.3	80:10:10	PVDF:carbon black - copper	18
MoS_2 Nanobelts	20	80:10:10	sodium alginate:carbon black - copper	19
1T MoS_2/Cu	3 – 4	80:10:10	PVDF:acetylene black - copper	20
1T MoS_2/C	0.1 – 1	80:10:10	CMC:Super P - copper	21
1T MoS_2/Graphene	-	80:10:10	CMC:acetylene black - copper	22

Note II: Pristine Powder and As-Cast Electrode Sample Characterization

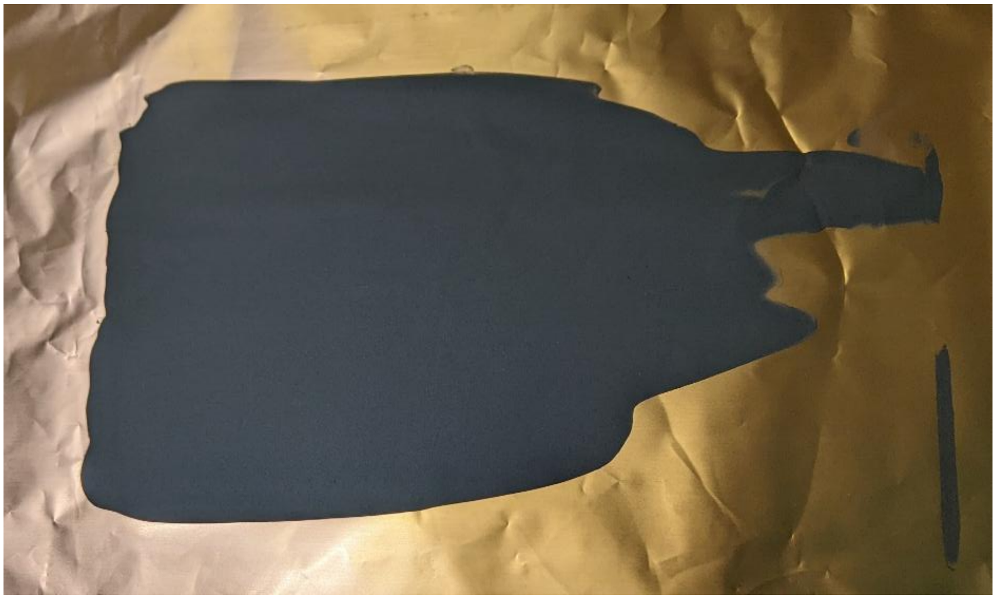

Figure S3. Top-down precursor MoS_2 battery electrode with a ~ 36 µm coating thickness on a ~ 11 µm copper foil current collector. Figure adapted with permission from source[23].

The MoS_2 electrode coatings used within the study possess a thickness in the range of **12** µm – **89** µm, with the main sample being ~ 36 µm thick and having a blue/grey color (Figure S3). All electrodes made are composed of 80 wt.% MoS_2, 10 wt.% PVDF binder, and 10 wt.% carbon Super P and slurry-cast using a doctor blade. This is a common electrode configuration (Table S2) for electrochemical studies involving commercial MoS_2 in Li-ion batteries.

All XPS samples are vacuum dried for 24+ hours at 60°C before analysis with a XPS spectrometer, to diminish the effect of surface contaminants. As-cast electrode samples are first characterized with SEM (Figure S5), XRD (Figure S6), and Raman spectroscopy (Figure S7), and then scanned with XPS under argon ion bombardment.

SEM images of the as-cast ~ 36 µm thick electrode show a surface morphology dominated by large MoS_2 flakes lying laterally on their basal plane (Figure S5). Very few flakes are vertically aligned, indicating a general difficulty in stabilizing flakes in this direction. There is a significant variation in the flake lateral size, with the smallest flakes measuring as little as 1 µm across (Figure S5d – green arrows) and the largest up to 10 µm (Figure S5d – purple arrows).

The surface of the flakes appears textured and dark, with well-defined edges, grooves, flake lines, and a clear separation between neighboring flakes. Surrounding the MoS_2 flake network, exists the much smaller supporting fluffy carbon conductive additive (Figure S5d – red square). It can be observed spread throughout the electrode surface.

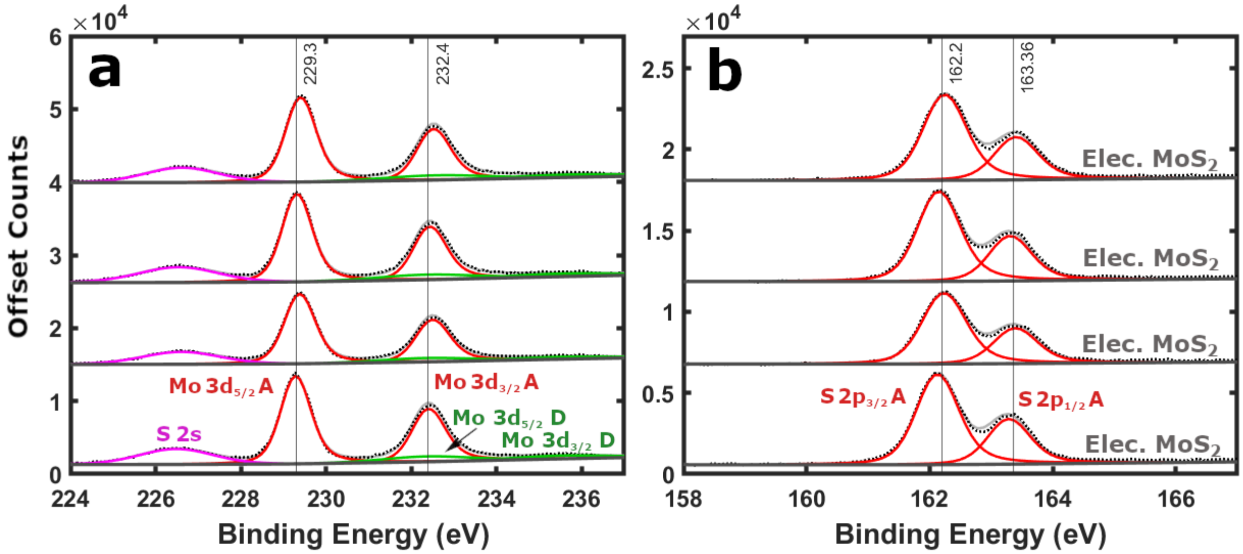

Figure S4. XPS of as-cast battery electrodes. Black dotted lines represent the raw XPS data, light grey solid lines depict the fitting envelope, and dark grey solid lines display the fitting background. Solid colored lines signify peak fits, such as the Mo 3d split orbit peaks (POS-A red), the S 2p split orbit peaks (POS-A red), and the single S 2s peak (pink). (a) Mo 3d and (b) S 2p scan regions. Data adapted with permission from source[23].

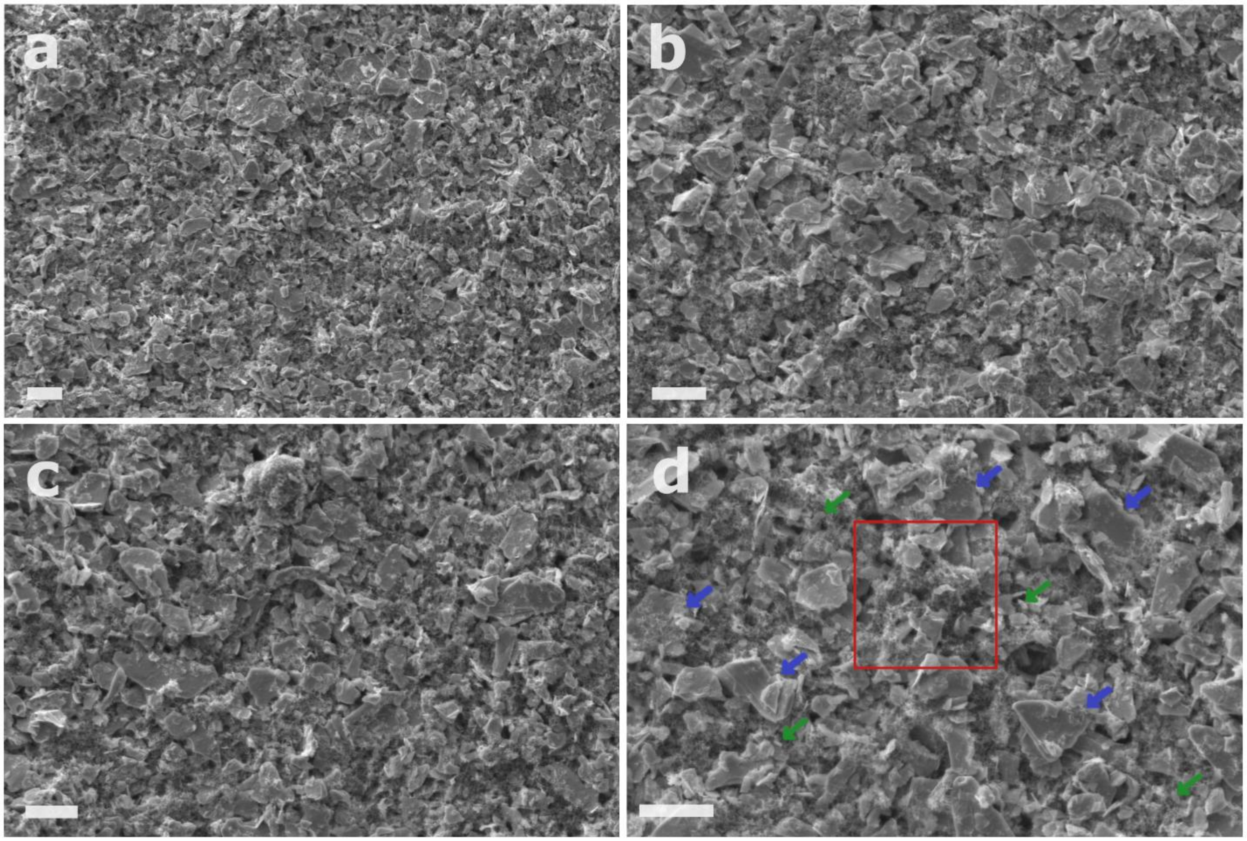

Figure S5. SEM of as-cast top-down precursor MoS_2 battery electrode with coating thickness of approximately ~ 36 µm. All scale bars are 10 µm. Green arrows denote flakes ~ 1 µm, purple arrows identify flakes ~ 10 µm, and the red rectangle indicates a region where the conductive carbon Super P network can be seen. Figure adapted with permission from source[23].

XRD diffraction patterns of as-cast top-down precursor MoS_2 electrodes indicate a clear 2H bulk MoS_2 structure irrespective of the electrode thickness (Figure S6a), due to the presence of ICSD-84180 2H MoS_2 indices 002, 004, 006, 008, 100, 101, 102, 103, and 105 at 14.4°, 29.1°, 44.2°, 60.2°, 32.7°, 33.5°, 36.0°, 39.6°, and 49.9°. In all cases, the MoS_2 002 index dominates the diffraction patterns, alongside the higher order 00*l* family indices 004, 006, and 008. Therefore, indicating a preferential deposition orientation[24] of MoS_2 in slurry-cast electrodes with the MoS_2 flakes lying mostly flat on their basal plane as observed with SEM (Figure S5).

Depending on the thickness of the electrode, the intensity of the copper Cu 111 (43.5°), 200 (50.6°), and 220 (74.1°) indices vary. As the thickness of the MoS_2 coating increases, the MoS_2 peaks increase and the copper peaks decrease (Figure S6a), whereby at a thickness of 87 µm the copper indices vanish altogether from the diffraction pattern.

The fluctuation of the MoS_2 peak positions, best observed for the 002 index (Figure S6a), is caused by difference in the height of the sample and the lack of height calibration for the Bruker D2 phaser diffractometer. To validate this claim, the copper current collector indices can be used as a reference (Figure S6b). Since the copper positions move in conjunction with the MoS_2 006 index it is verified that the sample height differences cause the peak alignment deviation.

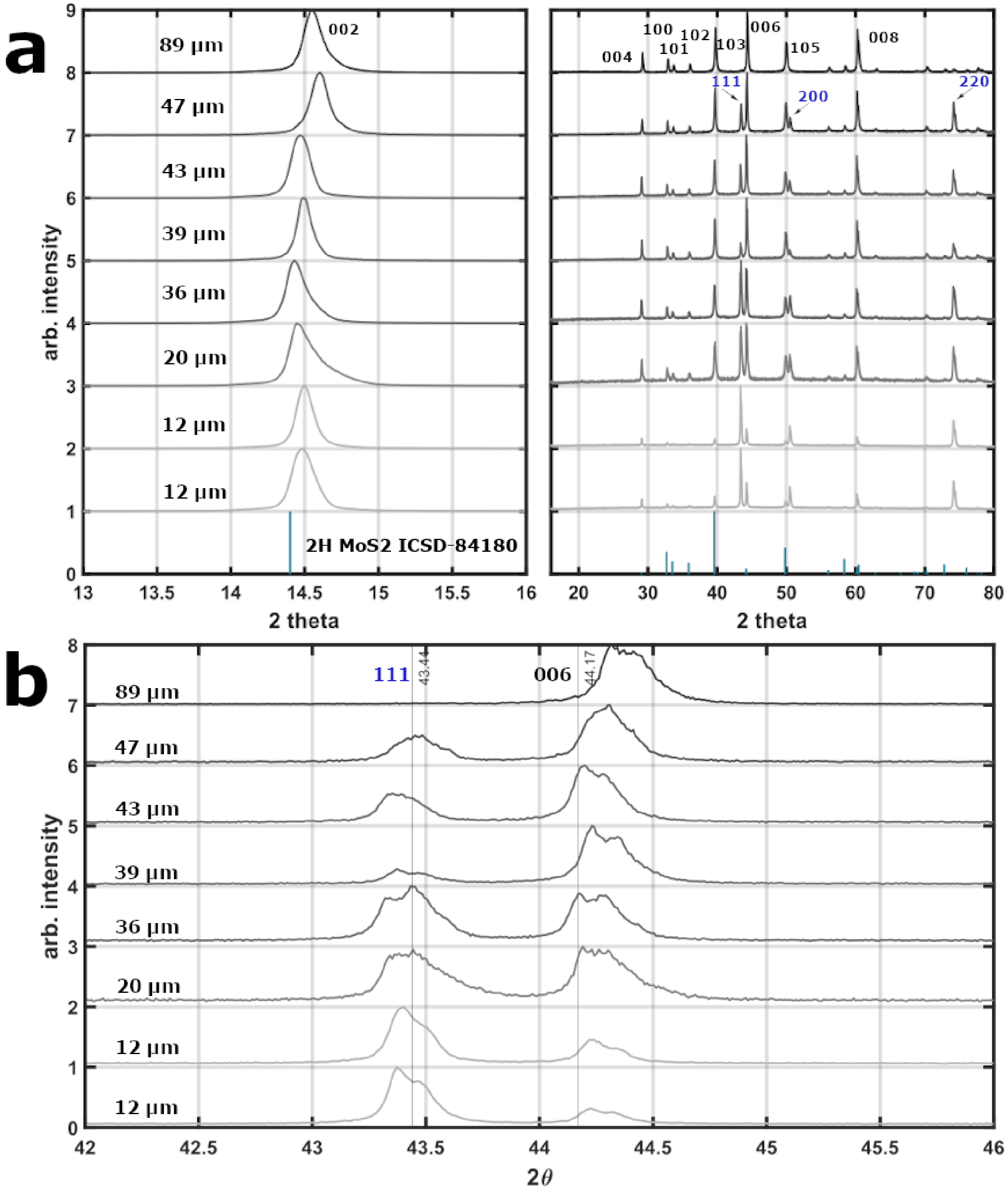

Figure S6. As-cast top-down precursor MoS_2 battery electrodes of varying thickness analyzed with XRD using a Cu-source. The black XRD indices denote 2H MoS_2 whereas the blue indices represent copper from the electrode current collector.

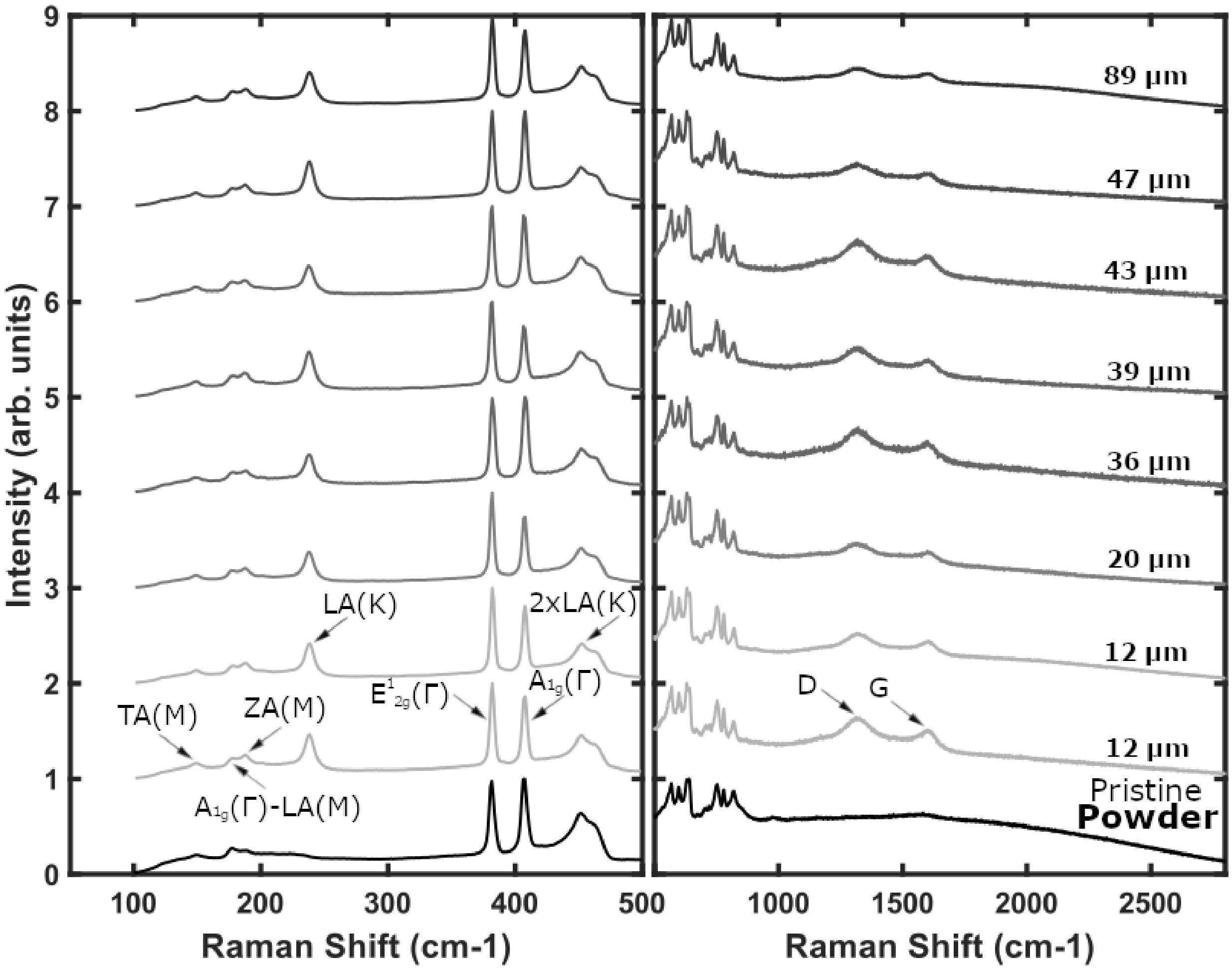

Figure S7. As-cast top-down precursor MoS_2 battery electrodes of varying thickness analyzed with Raman spectroscopy employing a 785 nm laser.

Considering Raman spectroscopy, the spectra display a clear 2H phase MoS_2 signal for the powder and all thicknesses of as-cast MoS_2 battery electrodes (Figure S7). The dominant Raman peaks are the $E^1_{2g}(\Gamma)$ (381 cm^{-1}) and $A_{1g}(\Gamma)$ (407 cm^{-1}) modes, with lower shift bulk MoS_2 peaks TA(M) (148 cm^{-1}), $A_{1g}(\Gamma)$-LA(M) (177 cm^{-1}), ZA(M) (187 cm^{-1}), and LA(K) (238 cm^{-1}) also visible in all electrodes[25].

The presence of the lower range modes is commonly misinterpreted as identification of the 1T MoS_2 phase J_1, J_2, J_3, and Z_1 modes. However, in 2H phase bulk top-down precursor MoS_2 irradiation with a 785 nm laser wavelength can induce their presence[25], due to the laser energy being in proximity to the bandgap of the material. This does not occur for laser wavelengths below 633 nm. The only distinction between the 2H MoS_2 powder and the as-cast electrodes is the presence of the MoS_2 LA(K) (238 cm^{-1}) mode and the carbon G and D peaks from the carbon Super P in the electrodes (Figure S7).

Note III: MoO_3 XPS Fitting

In all cases throughout the study, the minor presence of MoO_3 has been considered with the modelling of the split-peak orbital peaks POS-D. Since, if MoO_3 is partially ignored by leaving out the Mo $3d_{5/2}$ POS-D peak (Figure S8 Fit I & Fit II) as is common practice in the literature, the theoretical area correlation between the Mo $3d_{5/2}$ and $3d_{3/2}$ POS A peaks of 66.7 % cannot be observed.

Instead, the least error minimization fitting indicates a 71% correlation (Figure S8 Fit I). Including the theoretical 66.7% restraint in Fit II results in an increased STD, as part of the higher binding energy peak cannot be accounted for. To maintain the theoretical limits of the model, an additional peak needs to make up for the extra area in the spectrum. Furthermore, each Mo specie ought to possess two peaks for the Mo 3d region due to orbital splitting.

Thus, the small Mo $3d_{5/2}$ POS-D peak is introduced (Figure S8). Mathematical minimization suggests a 61% correlation for the POS-A peaks (Fit III), but this deviates from the theoretical limits. However, restraining the POS-A peak relationship to 66.7% does not cause a great increase in the STD fit value (Figure S8), and is therefore implemented across all fits in the study.

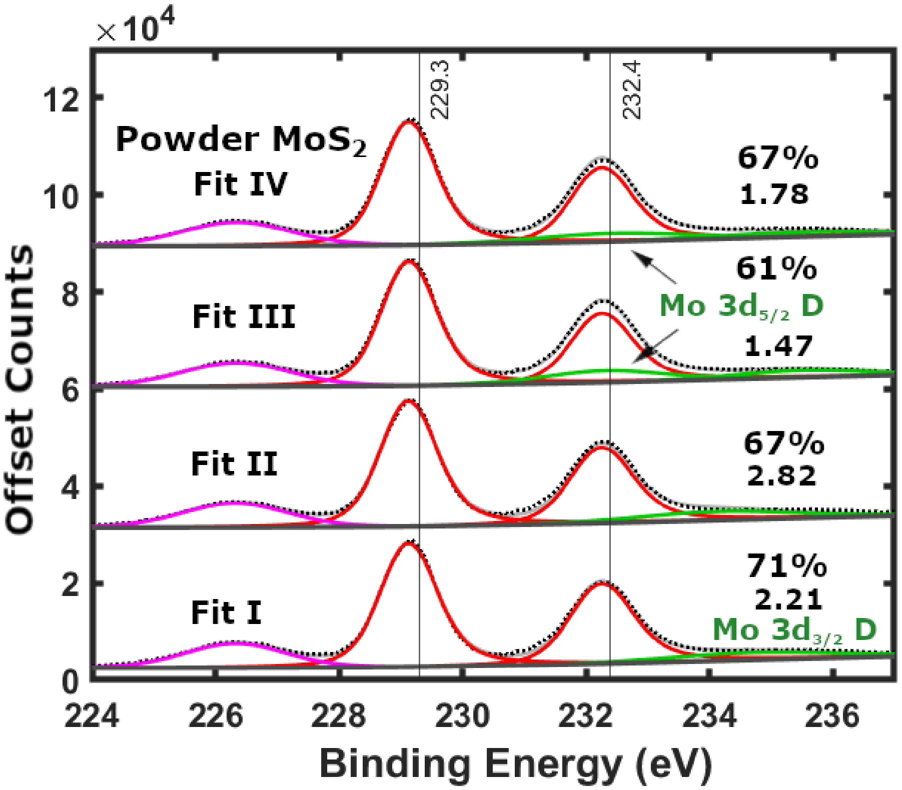

Figure S8. XPS fitting models for pristine top-down precursor MoS_2 powder. Black dotted lines represent the raw XPS data, light grey solid lines depict the fitting envelope, and dark grey solid lines display the fitting background. Solid colored lines signify peak fits. Area percentage (%) correlation of Mo $3d_{3/2}$ to Mo $3d_{5/2}$ POS-A peaks and the model fit STD are shown for each case. Fit I and Fit II do not have a Mo $3_{5/2}$ POS-D peak. Additionally, Fit II and Fit IV are constrained by an area correlation of 66.7% for peak Mo $3d_{3/2}$ POS-A relative to Mo $3d_{5/2}$ POS-A.

Pristine & as-cast XPS Survey Spectra

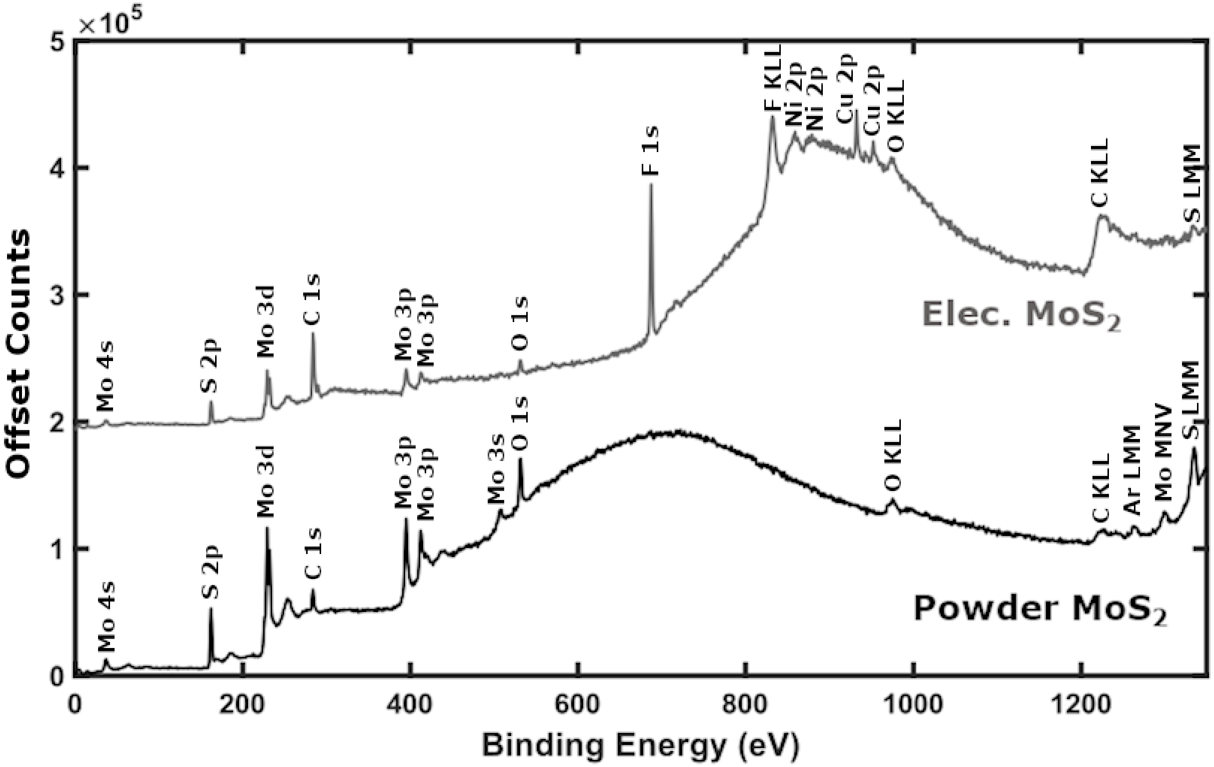

Figure S9. XPS survey spectra of pristine top-down precursor MoS_2 powder and as-cast battery electrode (~ 36 μm thick).

The powder survey scan (Figure S9) is marked by Mo 4s (64 eV), the S 2p doublet (162 eV), the Mo 3d doublet (229 eV), adventitious C 1s (284 eV), the higher orbital Mo 3p doublet (395 eV and 413 eV), Mo 3s (507 eV), O 1s (531 eV), the Auger O KLL (976 eV), the broad Auger C KLL (~ 1226 eV), the Auger Ar LMM (1262 eV), the Auger Mo MNV (1299 eV), and the Auger S LMM (1335 eV) peaks.

The as-cast electrode surface contains almost all the XPS peaks present in the powder used to make it, however; there are peak changes and new additions to account for. The electrode survey spectrum is dominated by the F 1s (687 eV) and Auger F KLL peaks (832 eV) from the PVDF binder despite it making up only 10 wt. % of the coating (Figure S9). The increased carbon C 1s peak is due to the conductive Super P additive. Whereas the MoS_2 peaks (Mo 3d, Mo 4s, and S 2p) are significantly reduced in intensity. Only the Auger C KLL peak is noticeably pronounced in the electrode, with a minute presence from S LMM.

The coating also contains contaminants such as the Cu 2p couplet (932 eV and 952 eV) from the electrode current collector and the Ni 2p couplet (861 eV and 877 eV) from an unknown source (Figure S9). The presence of copper is not caused by detection of the current collector through the material coating (~ 36 μm), as the surface oriented XPS technique only has a penetration depth of approximately 10 nm[26]. Therefore, sample handling is more likely to have resulted in copper contamination.

Sample Composition

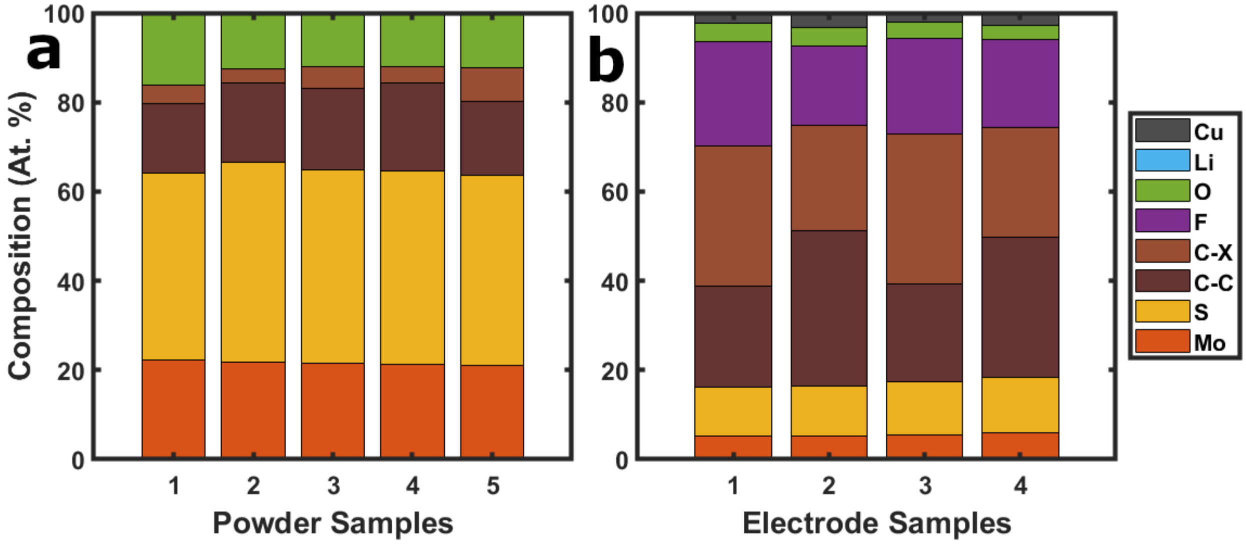

Figure S10. XPS elemental composition of pristine top-down precursor MoS_2 (a) powders and (b) as-cast battery electrodes (~ 36 µm thick).

Note IV: XPS Survey Depth-Profiling

Etching the surface of MoS_2 powder with Ar^+ ions, results in no distinct change in the survey spectrum (Figure S11a). The Mo 3d peaks remain dominant in intensity, followed by the Mo 3p, and S 2p peaks. With etching, the O 1s and C 1s peaks reduce as surface contaminants are removed from the sample surface. The only noticeable change induced in the survey spectrum is the appearance of the Ar LMM, Mo MNV, and S LMM Auger peaks.

On the other hand, the effect of etching an as-cast MoS_2 electrode (Figure S11b) involves the increased intensity of the Mo 3d, Mo 3p, and Mo 3s regions, the significant reduction of the surface dominant F 1s and F KLL peaks from PVDF, the removal of the O 1s and O KLL surface adsorbed oxygen peaks, the reduction of the contaminant Cu 2p couplet, and no alteration of the C 1s and S 2p regions. C 1s peak is unaffected due to the presence of carbon-based conductive additive (Super P) throughout the material coating.

In both powder and electrode samples (Figure S11), etching is accompanied by the rise of the Auger peaks C KLL (1224 eV), Ar LMM (1263 eV), Mo MNV (1299 eV), and S LMM (1335 eV). The Ar Auger peak is a consequence of the ion bombardment where some Ar^+ ions adhere to the sample surface.

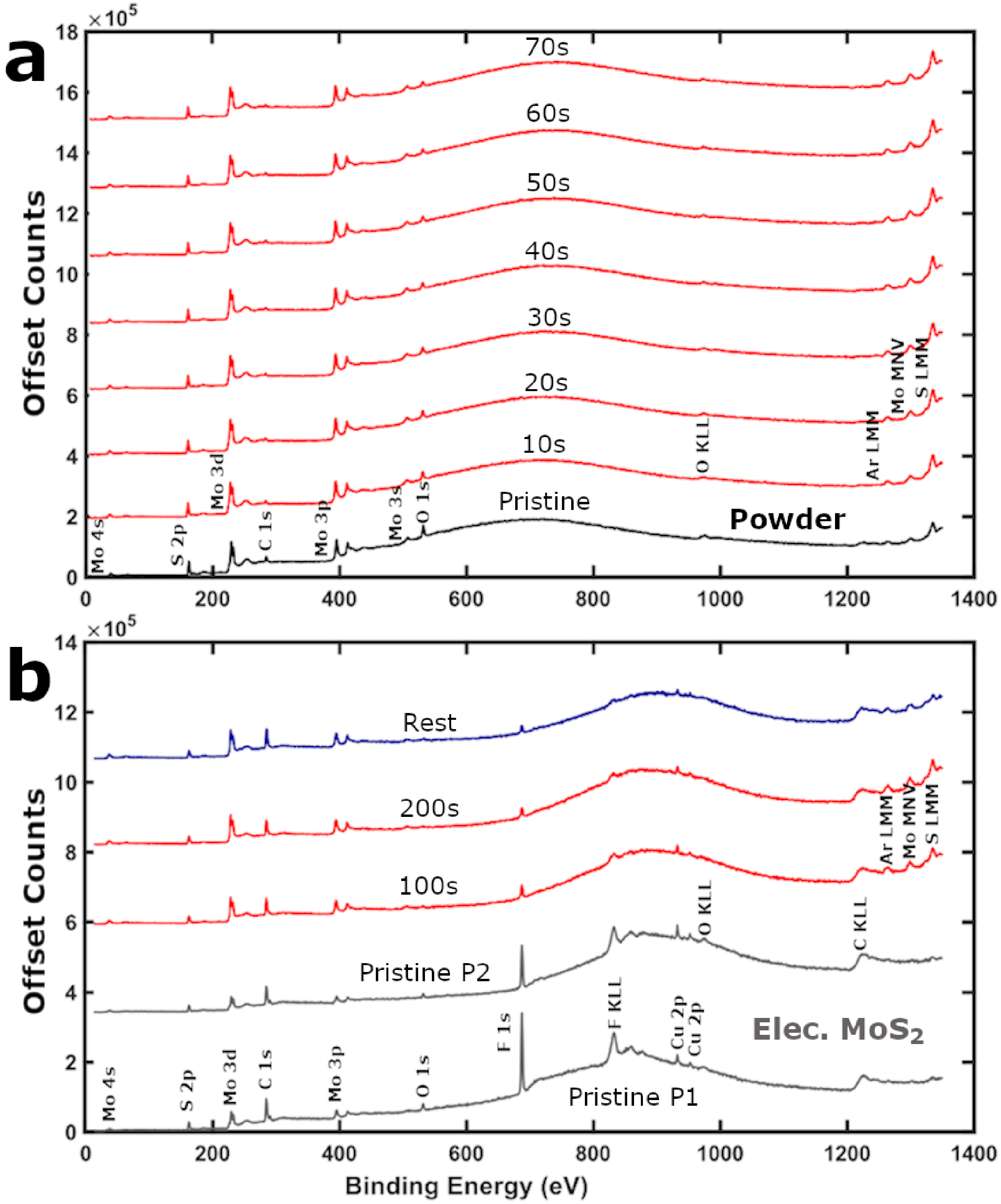

Figure S11. XPS Ar^+ ion depth-profiling of top-down precursor MoS_2 (a) powder and (b) as-cast battery electrode (~ 36 µm). Black/grey scans denote pristine/as-cast scan points, red indicates etched scan points, and blue denotes scans after 1 hour of rest in the XPS vacuum chamber following etching.

Powder MoS_2 Depth-Profiling (10s): Model Selection

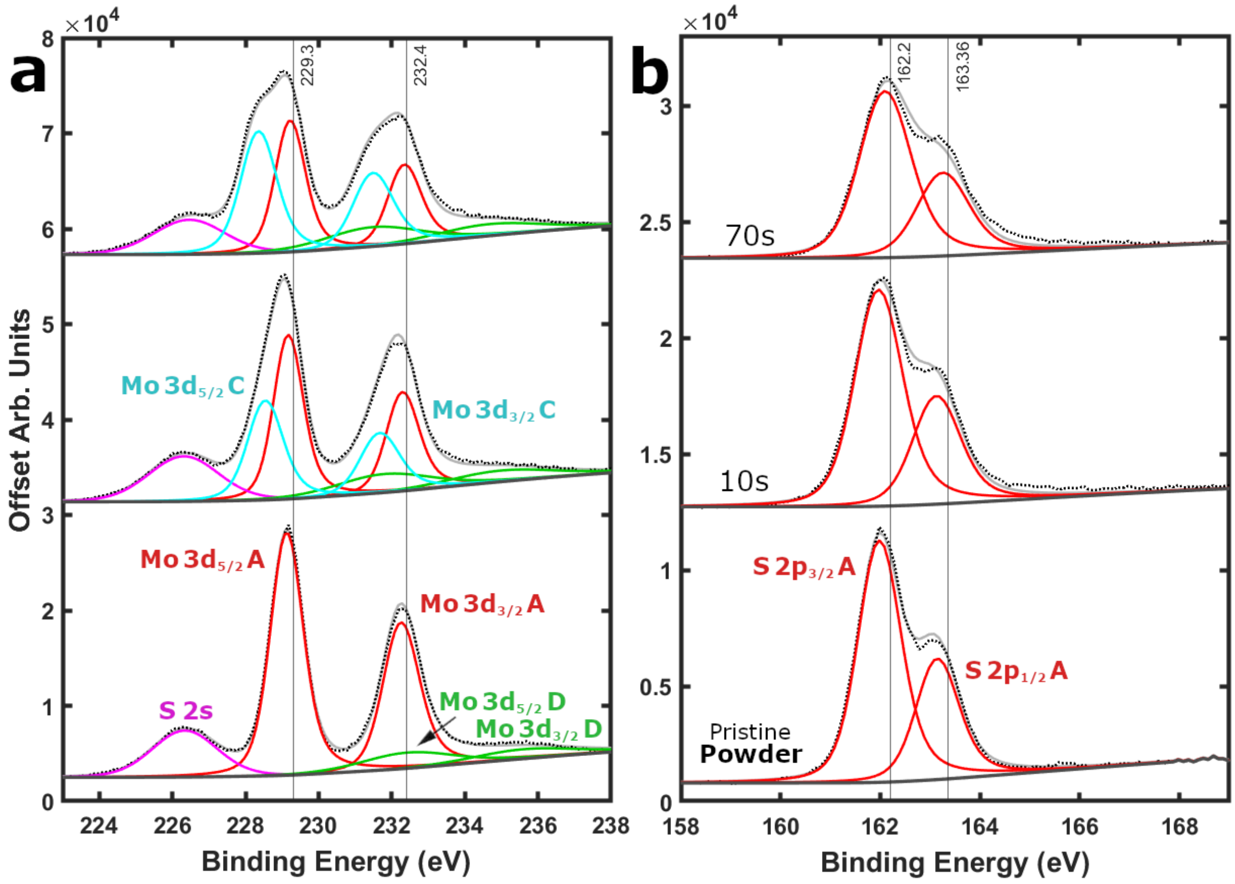

Figure S12. MoS_{2-x} fitting model for XPS data from top-down precursor MoS_2 powder depth-profiled by Ar^+ ions. Cumulative etch time displayed. Black dotted lines represent the raw XPS data, light grey solid lines depict the fitting envelope, and dark grey solid lines display the fitting background. Solid colored lines signify peak fitting, such as the Mo 3d split orbit peaks (POS-A red, POS-C cyan, and POS-D green) and the S 2p split orbit peaks (POS-A red). (a) Mo 3d and (b) S 2p scan regions.

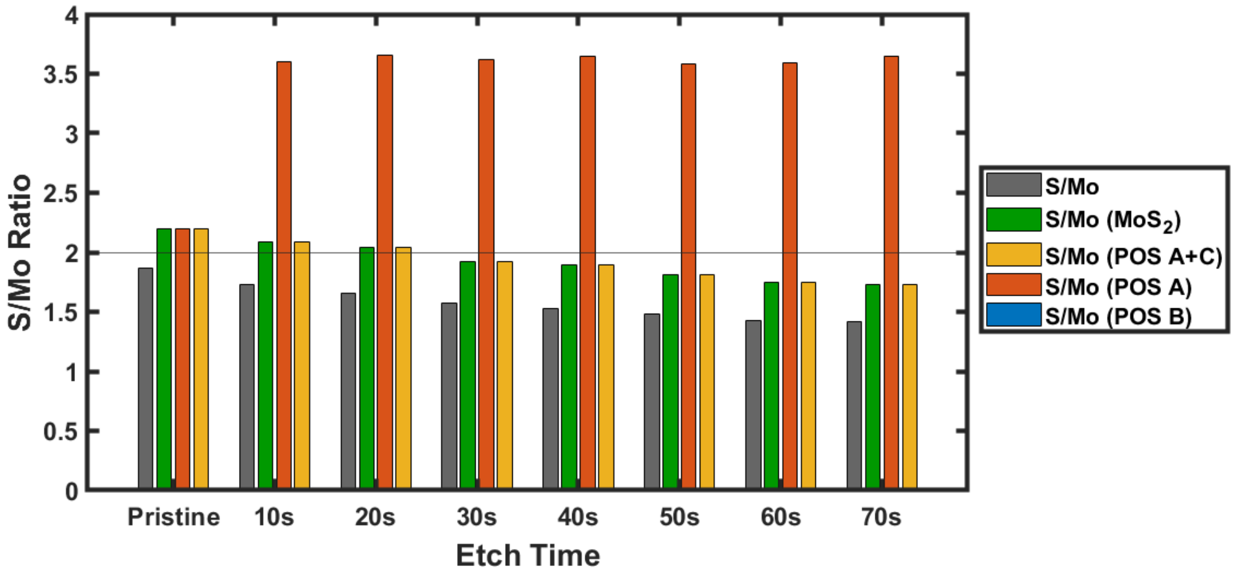

Figure S13. S/Mo XPS atomic ratios from fitting pristine MoS_2 powder sample XPS (Figure S12) with a MoS_{2-x} model (POS-A and POS-C peaks). S/Mo (grey) denotes the ratio of all Mo and S present in the sample including the Mo 3d MoO_3 specie, MoS_2 (green) represents all Mo and S collectively across all MoS_2 phases, POS-A (red) denotes Mo and S only from 2H MoS_2, and POS-A+C (yellow) represents Mo and S detected in the 2H MoS_2 and MoS_{2-x} phases.

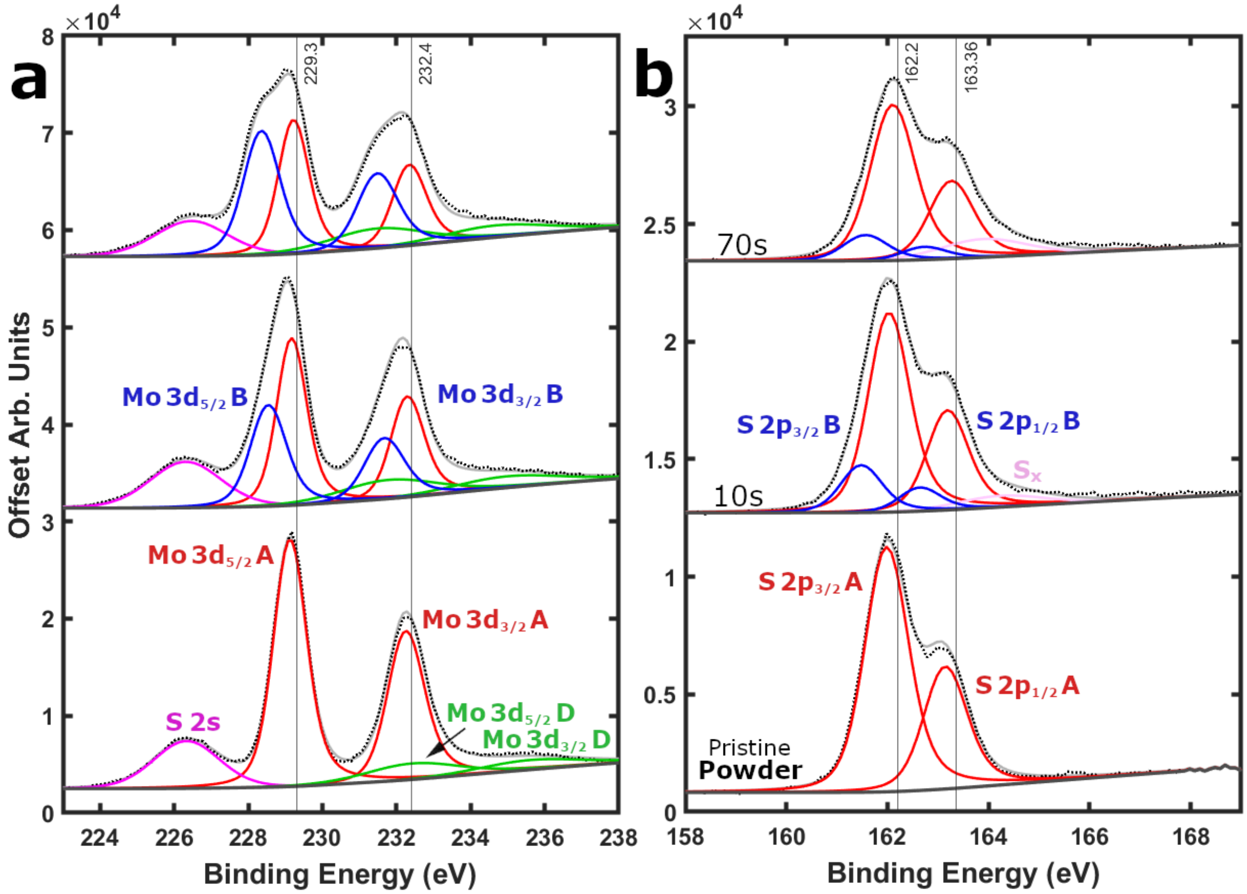

Figure S14. 1T MoS_2 fitting model for XPS data from top-down precursor MoS_2 powder depth-profiled by Ar^+ ions. Cumulative etch time displayed. Black dotted lines represent the raw XPS data, light grey solid lines depict the fitting envelope, and dark grey solid lines display the fitting background. Solid colored lines signify peak fitting, such as the Mo 3d split orbit peaks (POS-A red, POS-B dark blue, and POS-D green) and the S 2p split orbit peaks (POS-A red and POS-B dark blue). (a) Mo 3d and (b) S 2p scan regions.

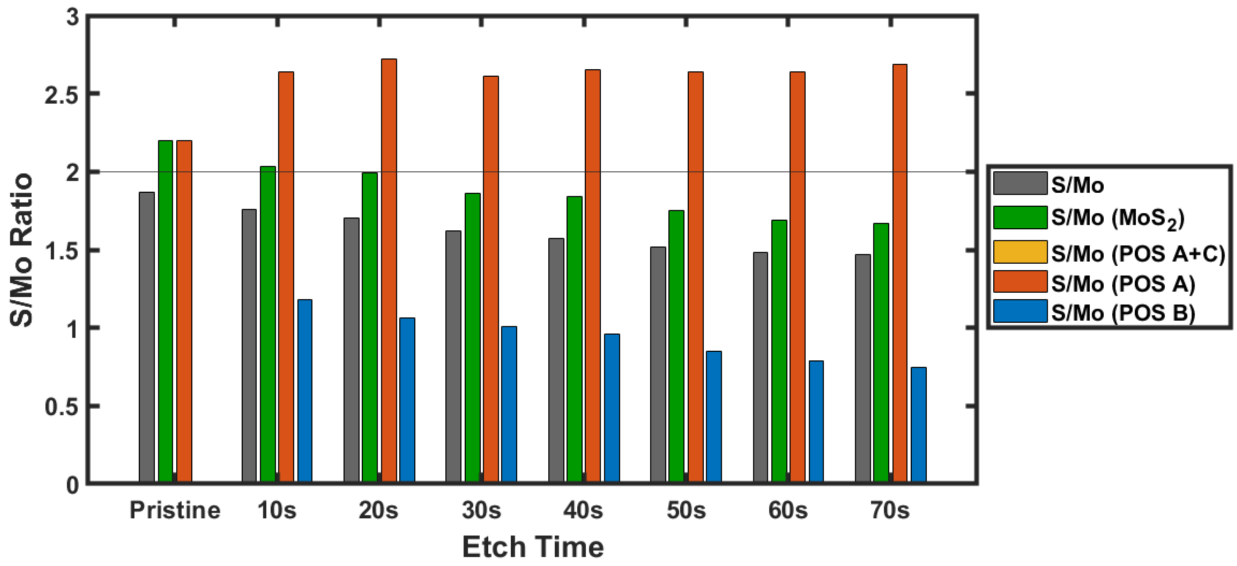

Figure S15. S/Mo XPS atomic ratios from fitting pristine MoS_2 powder sample XPS (Figure S14) with a 1T MoS_2 model (POS-A and POS-B peaks). S/Mo (grey) denotes the ratio of all Mo and S present in the sample including the Mo 3d MoO_3 specie, MoS_2 (green) represents all Mo and S collectively across all MoS_2 phases, whereas POS-A (red) and POS-B (blue – without S 2s) represent Mo and S exclusively detected in the 2H and 1T MoS_2 phases, respectively.

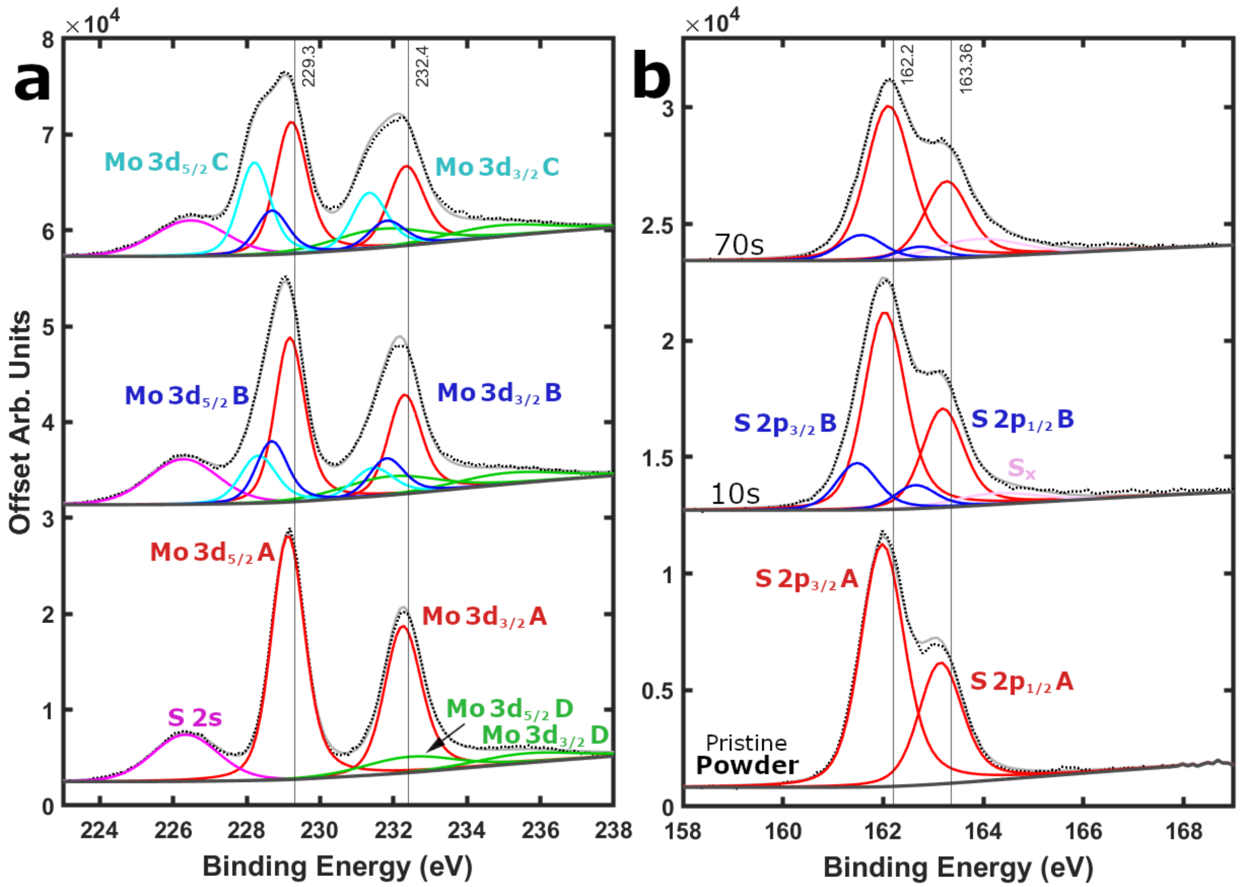

Figure S16. Four split orbit peak model fitting for XPS data from top-down precursor MoS_2 powder depth-profiled by Ar^+ ions. Cumulative etch time displayed. Black dotted lines represent the raw XPS data, light grey solid lines depict the fitting envelope, and dark grey solid lines display the fitting background. Solid colored lines signify peak fitting, such as the Mo 3d split orbit peaks (POS-A red, POS-B dark blue, POS-C cyan, and POS-D green) and the S 2p split orbit peaks (POS-A red and POS-B dark blue). (a) Mo 3d and (b) S 2p scan regions.

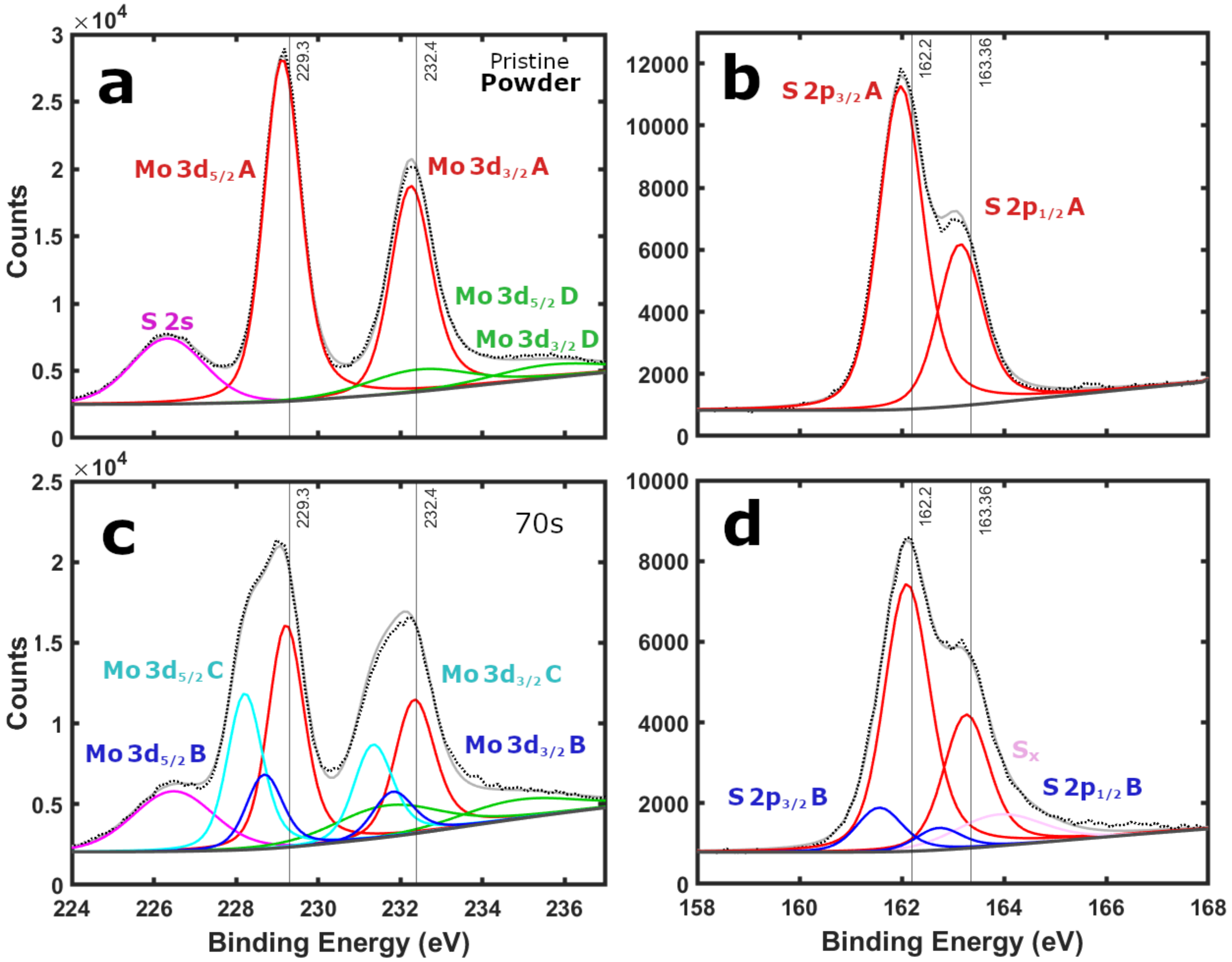

Figure S17. Residual plots for the four split orbit peak model applied to the top-down precursor MoS_2 powder depth-profiled by Ar^+ ions. Cumulative etch time displayed. Black dotted lines represent the raw XPS data, light grey solid lines depict the fitting envelope, and dark grey solid lines display the fitting background. (a – b) Pristine MoS_2 powder and (c – d) after 70s of etching.

Note V: Atomic Ratio S(POS-A)/Mo(POS-A + POS-C)

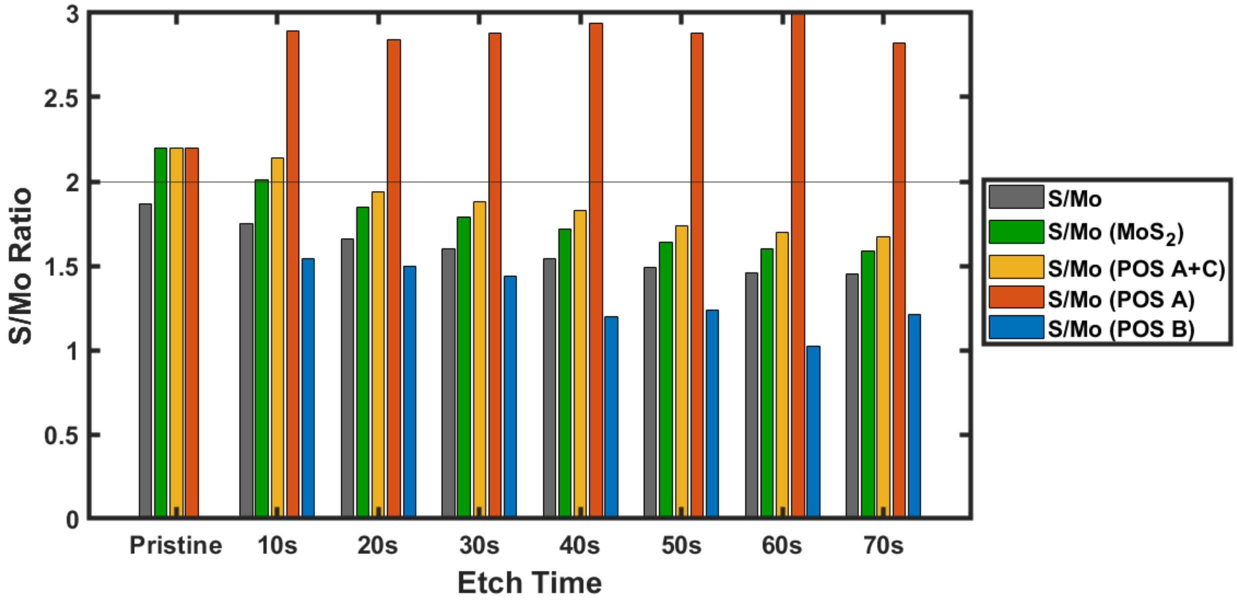

Figure S18. S/Mo XPS atomic ratios from fitting Ar^+ etched top-down precursor MoS_2 powder (Figure 4) with a four split orbit peak model. S/Mo (grey) denotes the ratio of all Mo and S present in the sample including Mo 3d MoO_3, MoS_2 (green) represents all Mo and S species collectively across all MoS_2 phases, whereas POS-A (red), POS-B (blue – without S 2s), and POS A + C (yellow) represent Mo and S species detected in the 2H MoS_2, 1T MoS_2, and MoS_{2-x} phases, respectively.

Applying the XPS four split orbit peak model (Figures 4 – 5 & S16), the overall atomic ratio in the sample decreases from 1.87 at the surface to 1.45 after 70s of depth-profiling (Figure S18). However, this atomic ratio considers all the molybdenum and sulfur present within the sample, including molybdenum found in MoO_3. Instead, using only the molybdenum and sulfur species in 1T MoS_2, 2H MoS_2, and MoS_{2-x} the S/Mo atomic ratio decreases from 2.2 in the pristine surface to 1.6 after 70s (Figure S18 – green). Both ratios agree that the overall S/Mo ratio in the sample decreases [1,27–29] with etching as sulfur is preferentially removed [1,28,30–32].

If one considers only the POS-A positions (Figure S18 – red), the S/Mo atomic ratio can be severely misleading as it surpasses 2.0 at every depth (2.8 – 3.0). Thus, giving the false impression that etching results in the preferential removal of Mo atoms. Similarly, by pairing the POS-C Mo 3d peaks with the POS-A S 2p peaks, the S/Mo atomic ratio would fall between 3.5 – 7.3 due to the lack of a POS-C sulfur signal, exceeding any reasonable stoichiometry.

However, by combining the Mo 3d POS-A and POS-C peak intensities against the POS-A S 2p peaks, a sulfur deficiency is observed in the S/Mo atomic ratio (Figure S18 – yellow). This unorthodox S/Mo pairing is plausible due to the 2H MoS_2/MoS_{2-x} sulfur environment being unchanged after preferential sulfur removal[1], alongside a significant change in the environment of the Mo atoms when missing a sulfur bond (Figure 4a).

Powder MoS_2 Depth-Profiling (10s)

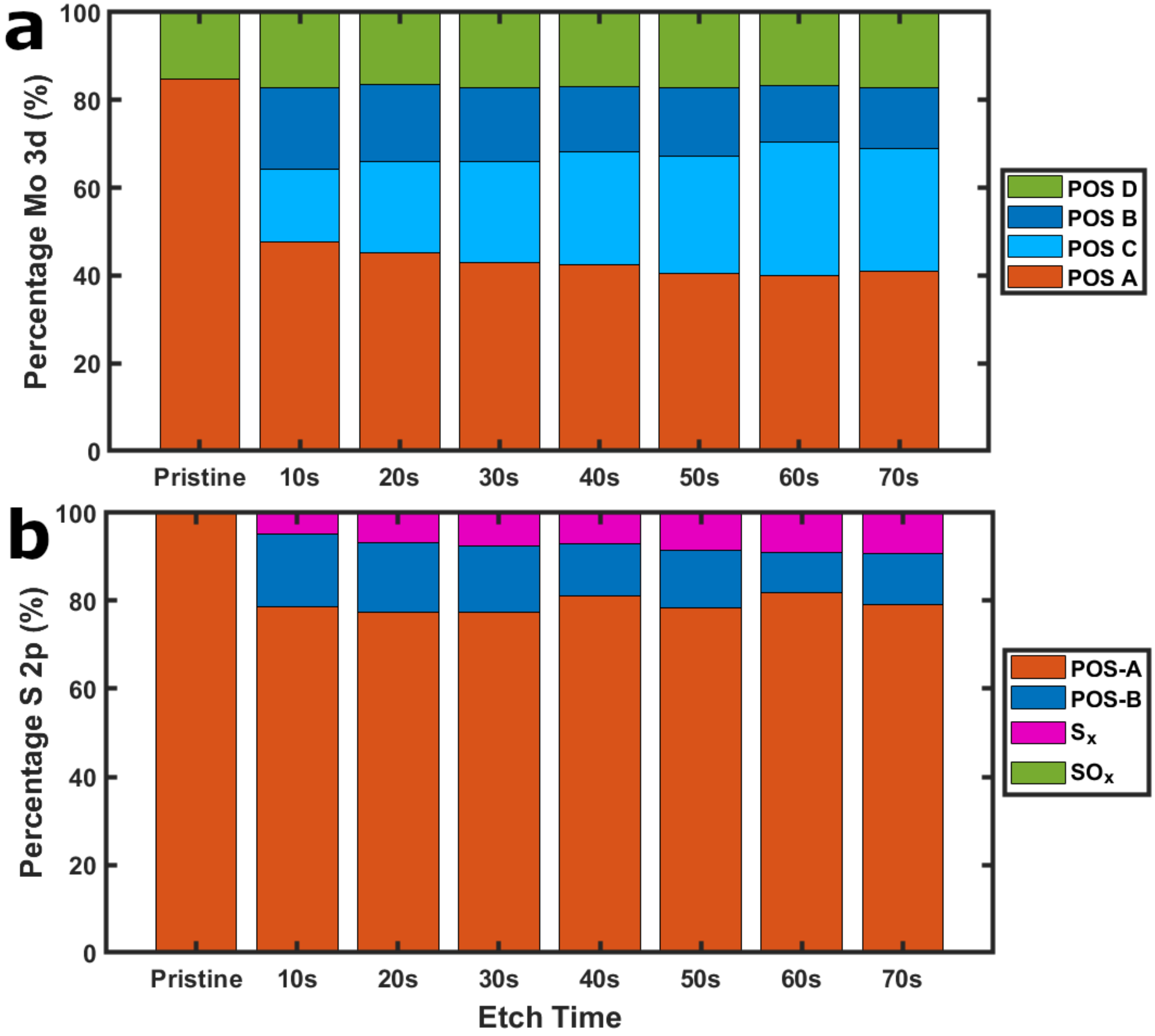

Figure S19. XPS composition by (a) Mo 3d percentage and (b) S 2p percentage for a pristine MoS_2 powder sample etched by Ar^+.

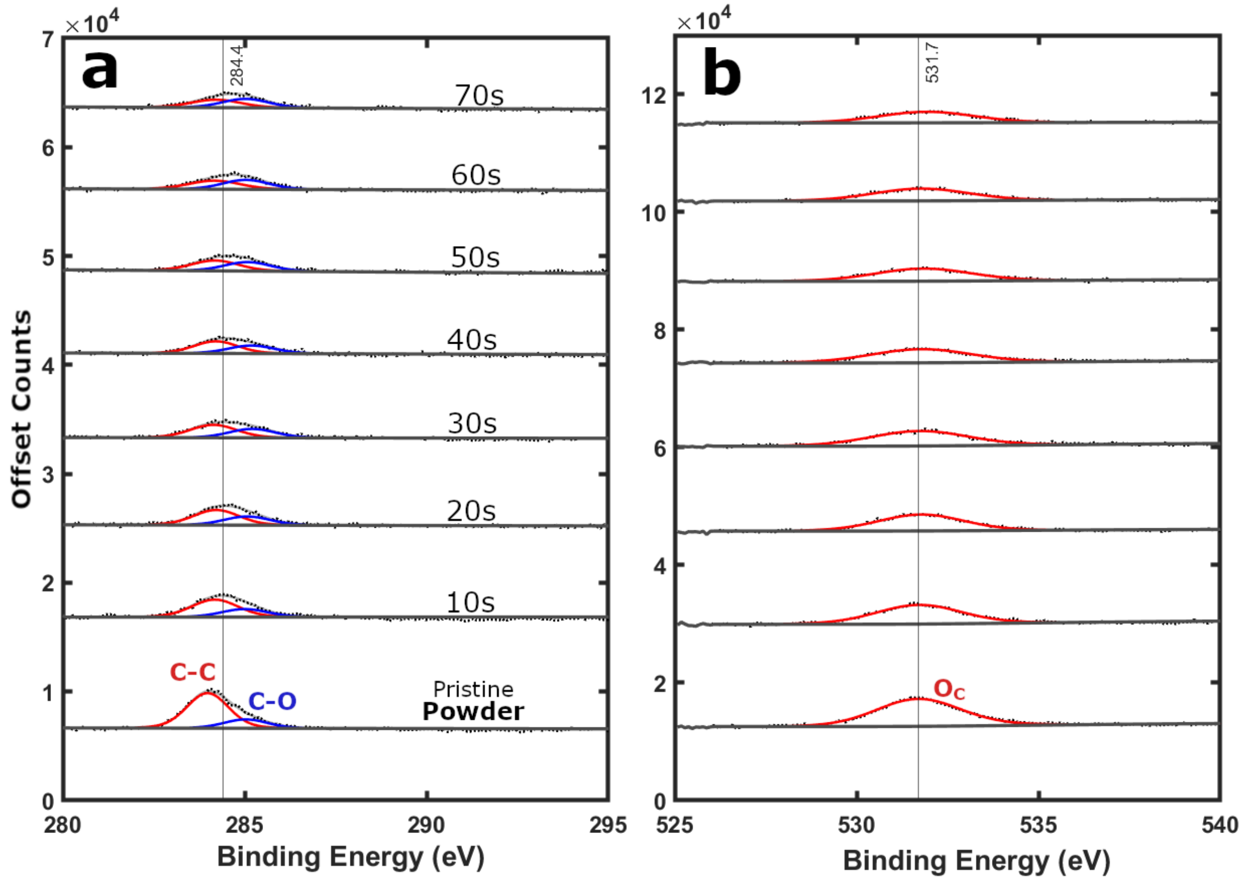

Figure S20. XPS of top-down precursor MoS_2 powder with Ar^+ ion 10s etch levels. Black dotted lines represent the raw XPS data, light grey solid lines depict the fitting envelope, and dark grey solid lines display the fitting background. (a) C 1s scan and (b) O 1s scan.

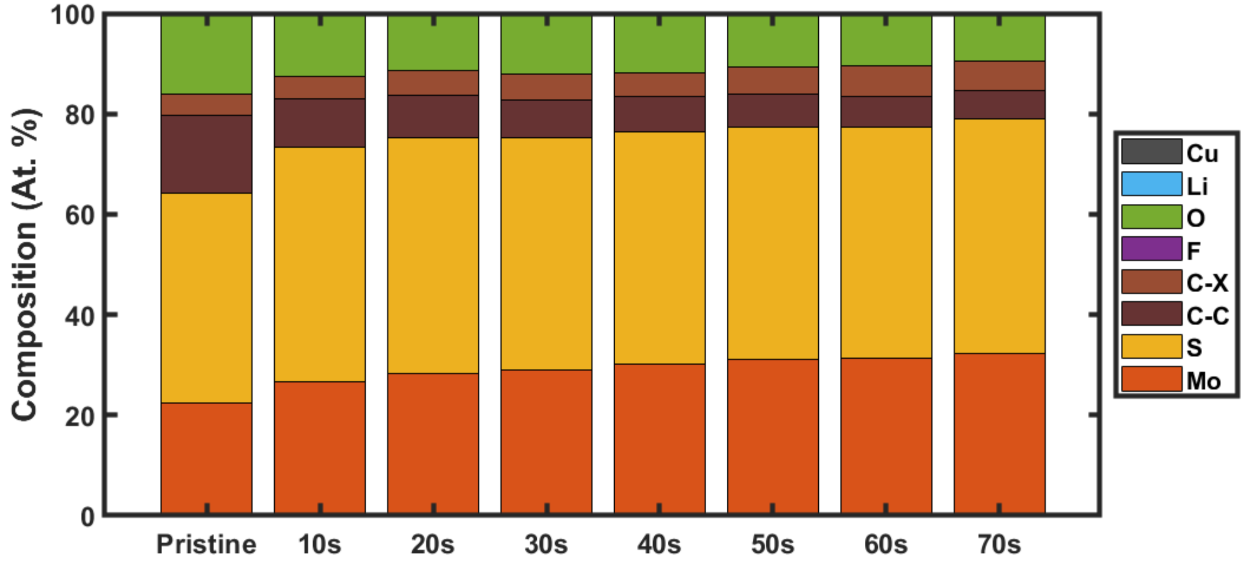

Figure S21. XPS elemental composition of top-down precursor MoS_2 powder with Ar^+ ion 10s etch levels.

Note VI: Surface Fluorine, Oxygen, & Carbon XPS Fitting

The following fitting is applied to the F 1s, O 1s, and C 1s (FOC) regions throughout the study where present:

The C 1s region is fit with up to five different peaks including C-C/C-H (284.0 eV – 285.0 eV), C-O/C-F (285.0 eV – 286.0 eV), C-O-C (286.0 eV – 287.0 eV), C=O (287.0 eV – 288.9 eV), and C_F (288.5 eV – 291.0 eV). Each peak is limited with an upper FWHM limit of 1.5, 2.0, or 3.0.

F 1s is fit by a single peak C-F (F_D ~ 687.3 eV) without any FWHM restrictions in pristine samples. For *ex situ* sample an additional Li-F peak (~ 685.5 eV) is considered.

The O 1s region is fit by a single peak C-O (O_C), whereby the range and FWHM of the O 1s peak are not constricted.

Furthermore, after LIB operation the *ex situ* electrodes are fit with Li 1s Li_A (~ 55.9 eV) and Li_B (~ 57.3 eV).

Powder MoS_2 Depth-Profiling (100s)

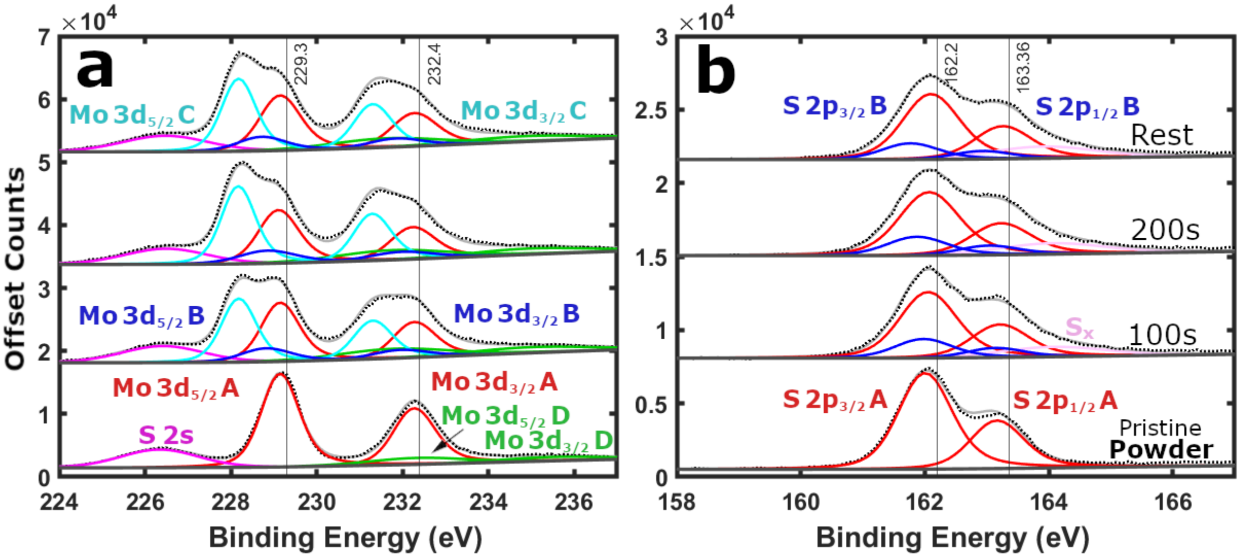

Figure S22. XPS of top-down precursor MoS_2 powder with rapid Ar^+ ion depth-profiling. Second sample for consistency. Black dotted lines represent the raw XPS data, light grey solid lines depict the fitting envelope, and dark grey solid lines display the fitting background. (a) Mo 3d and (b) S 2p scan regions.

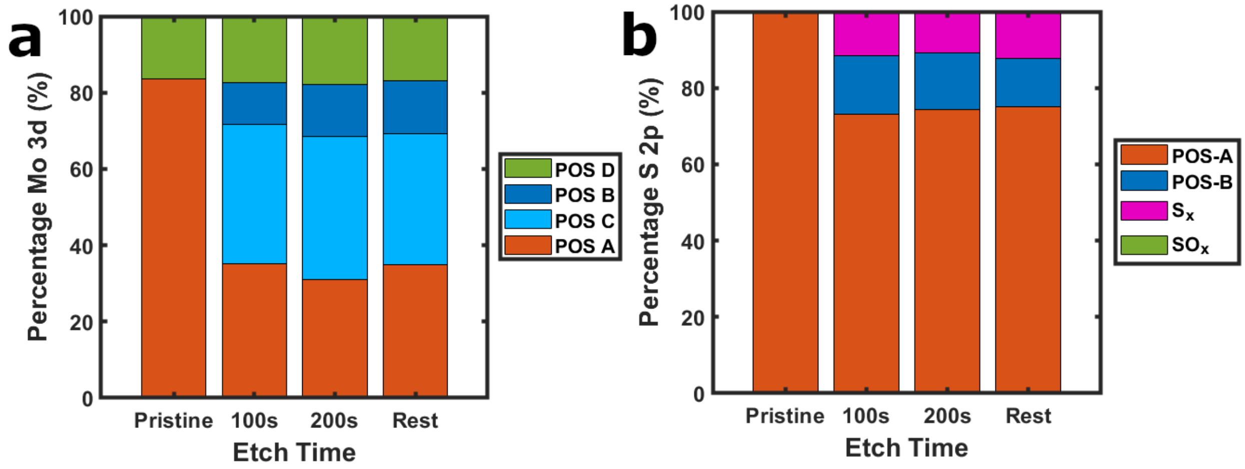

Figure S23. XPS composition by (a) Mo 3d percentage and (b) S 2p percentage for pristine MoS_2 powder sample etched by Ar^+.

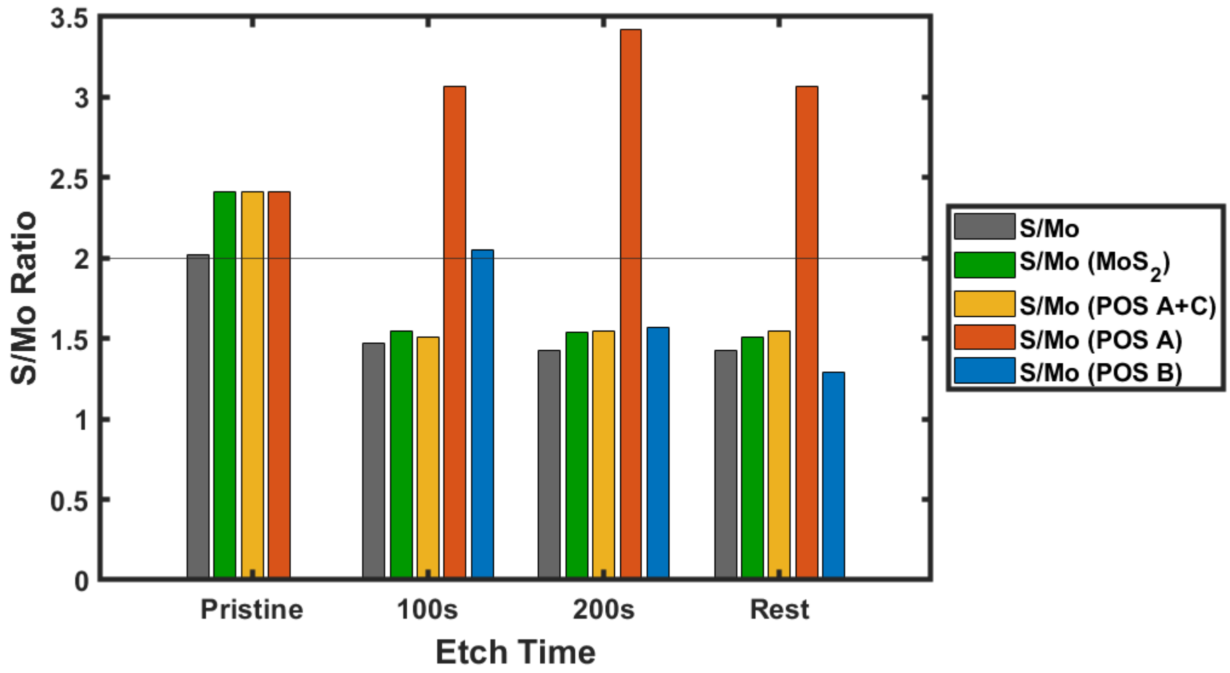

Figure S24. XPS S/Mo atomic ratio of top-down precursor MoS_2 powder with rapid Ar^+ ion depth-profiling (100s). S/Mo (grey) denotes the ratio of all Mo and S present in the sample including the Mo 3d MoO_3 specie, MoS_2 (green) represents all Mo and S collectively across all MoS_2 phases, whereas POS-A (red), POS-B (blue – without S 2s), and POS A + C (yellow) represent Mo and S detected in the 2H MoS_2, 1T MoS_2, and MoS_{2-x} phases, respectively.

MoS_2 Battery Electrode Depth-Profiling (100s)

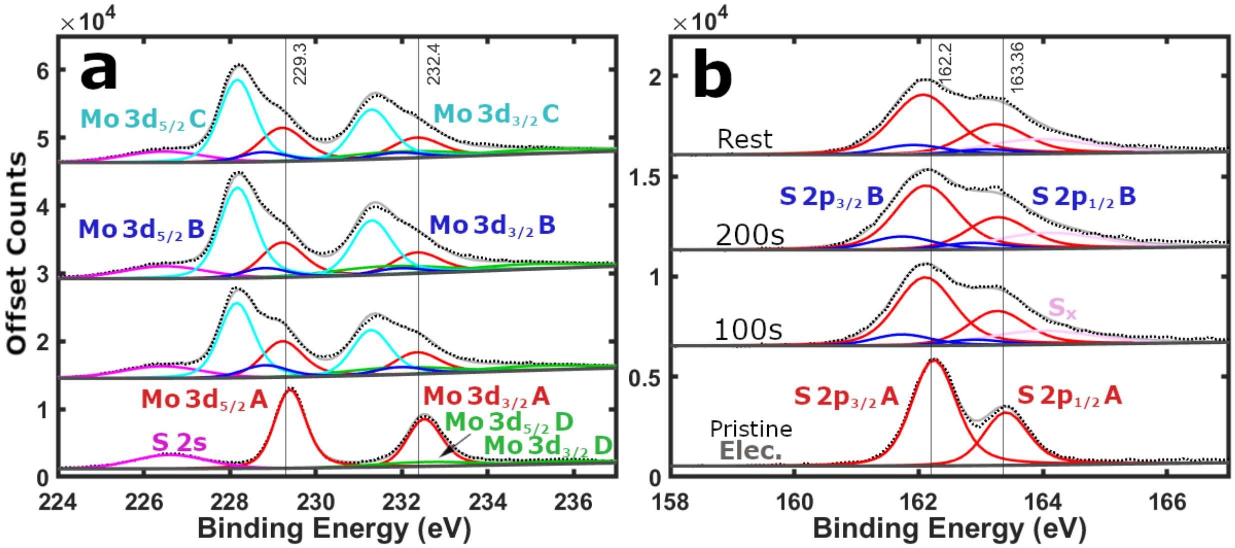

Figure S25. XPS of as-cast MoS_2 battery electrode with rapid Ar^+ ion etching. Second sample for consistency. Black dotted lines represent the raw XPS data, light grey solid lines depict the fitting envelope, and dark grey solid lines display the fitting background. (a) Mo 3d and (b) S 2p scan regions.

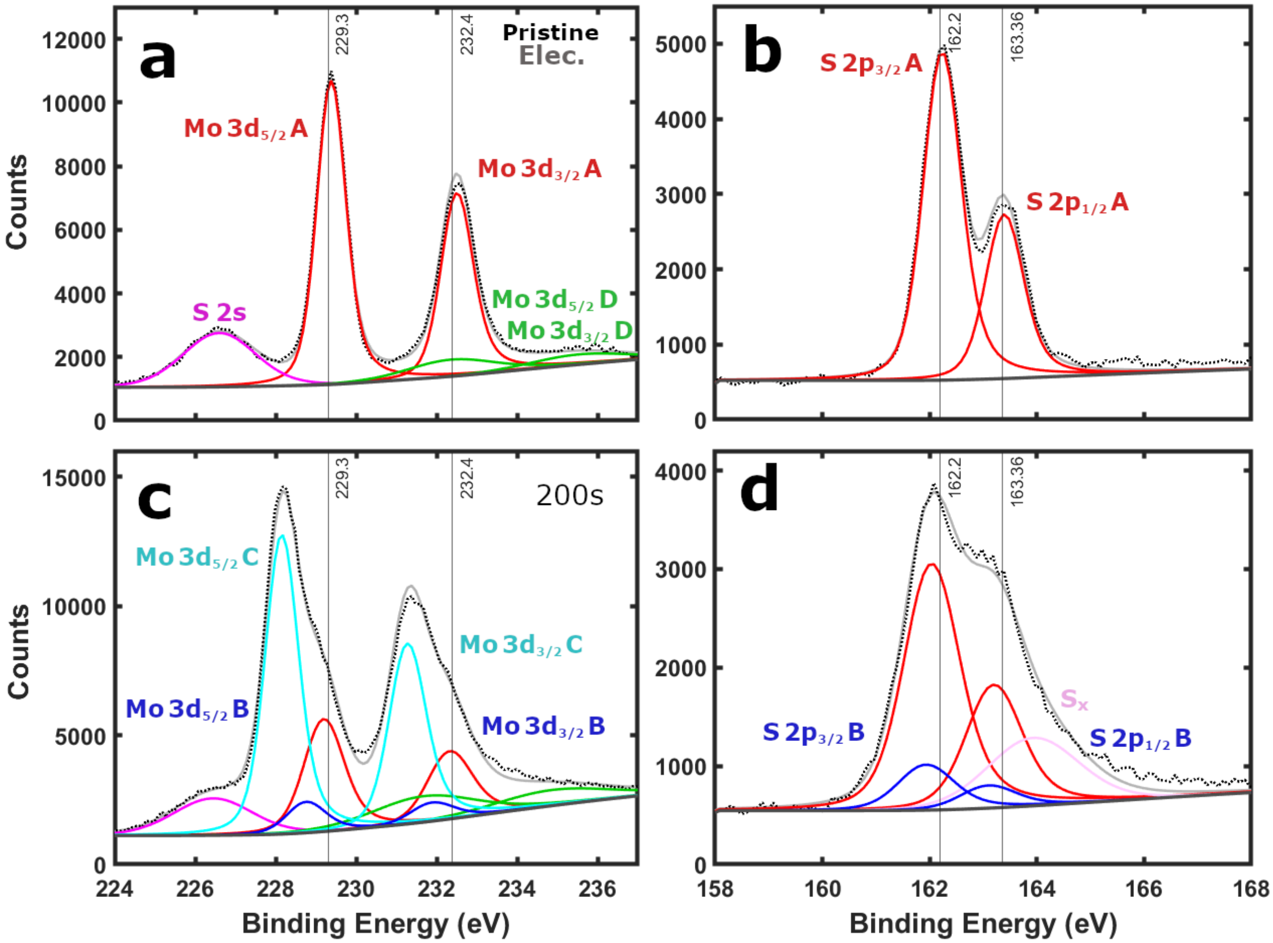

Figure S26. Residual plots for the four split orbit peak model applied to the as-cast MoS_2 electrode depth-profiled by Ar^+ ions. Cumulative etch time displayed. Black dotted lines represent the raw XPS data, light grey solid lines depict the fitting envelope, and dark grey solid lines display the fitting background. (a – b) as-cast MoS_2 electrode and (c – d) after 200s of etching.

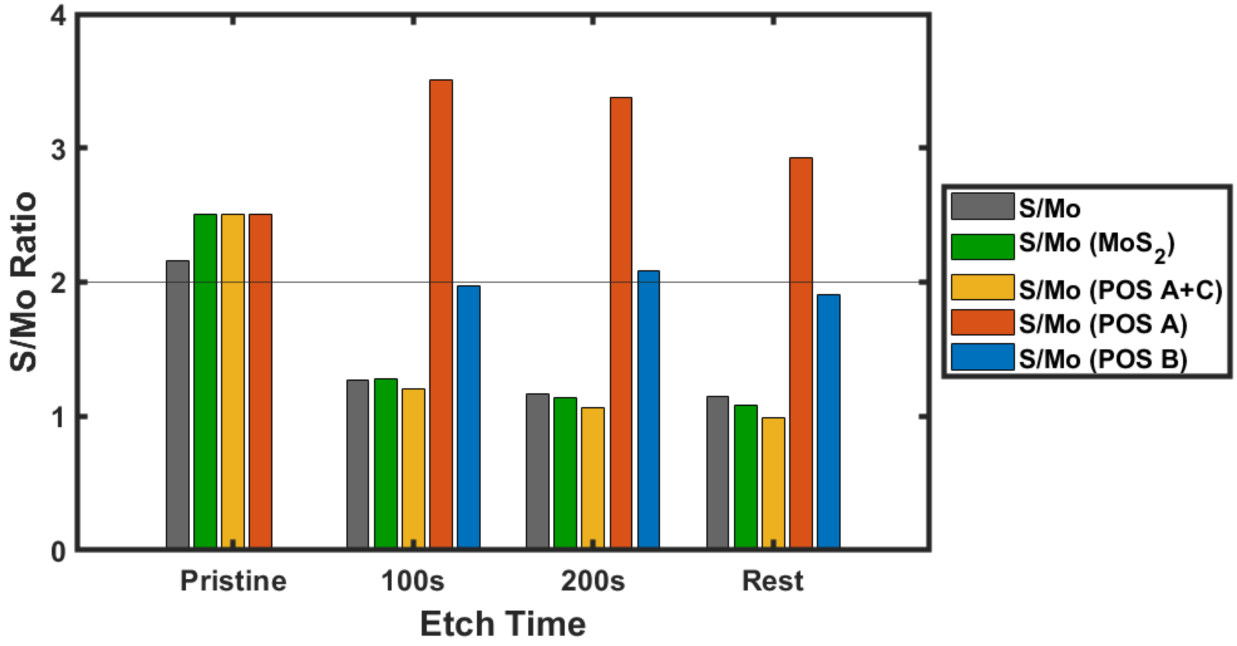

Figure S27. XPS S/Mo atomic ratio of as-cast MoS_2 battery electrode (Figure 7) with rapid Ar^+ ion depth-profiling (100s). S/Mo (grey) denotes the ratio of all Mo and S present in the sample including the Mo 3d MoO_3 specie, MoS_2 (green) represents all Mo and S collectively across all MoS_2 phases, whereas POS-A (red), POS-B (blue – without S 2s), and POS A + C (yellow) represent Mo and S detected in the 2H MoS_2, 1T MoS_2, and MoS_{2-x} phases, respectively.

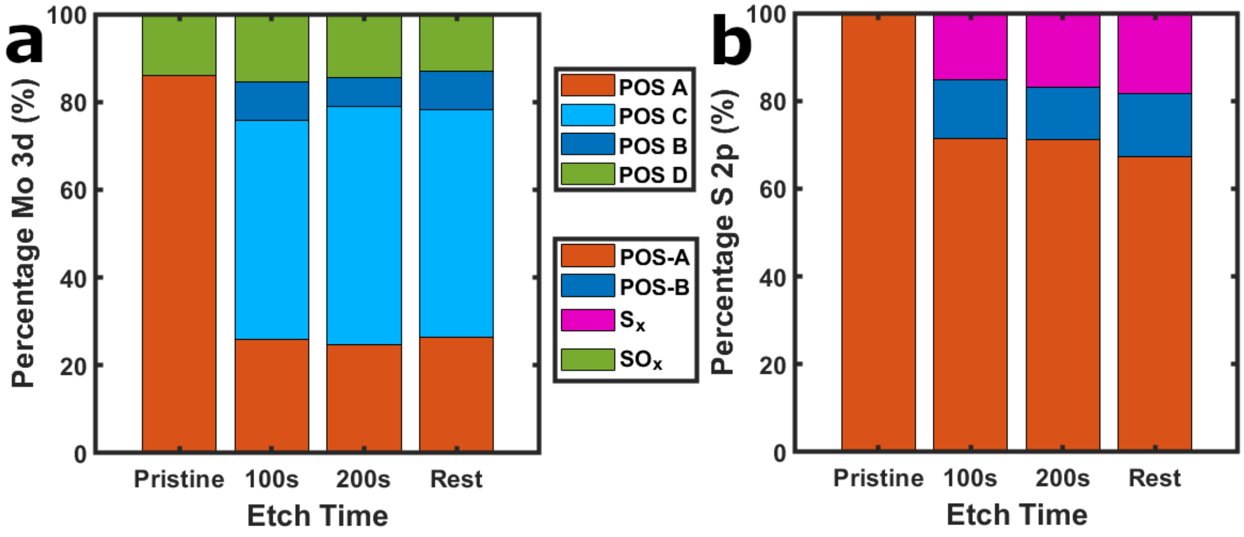

Figure S28. XPS composition for an as-cast MoS_2 battery electrode sample with rapid Ar^+ ion depth-profiling. (a) Mo 3d region and (b) S 2p region phase percentages.

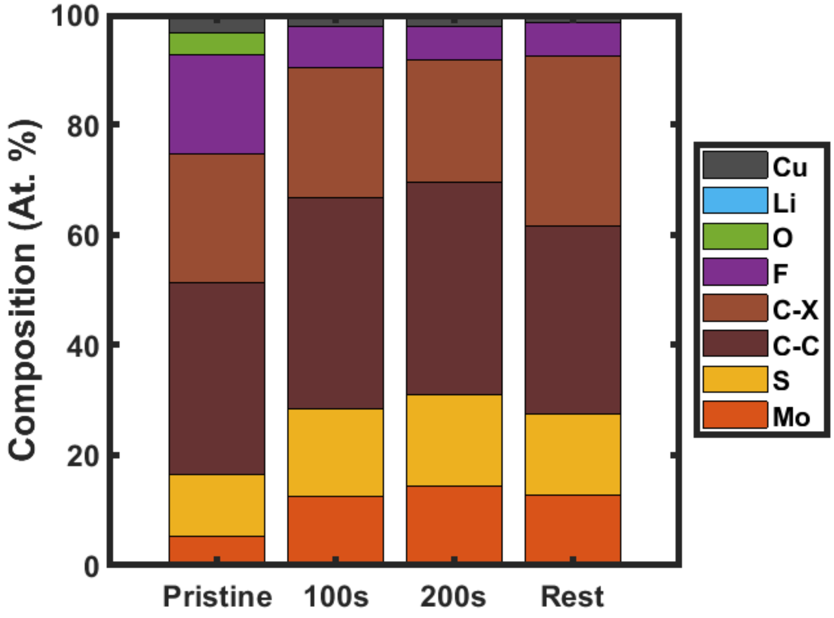

Figure S29. XPS elemental composition of as-cast MoS_2 battery electrode etched by Ar^+ ions.

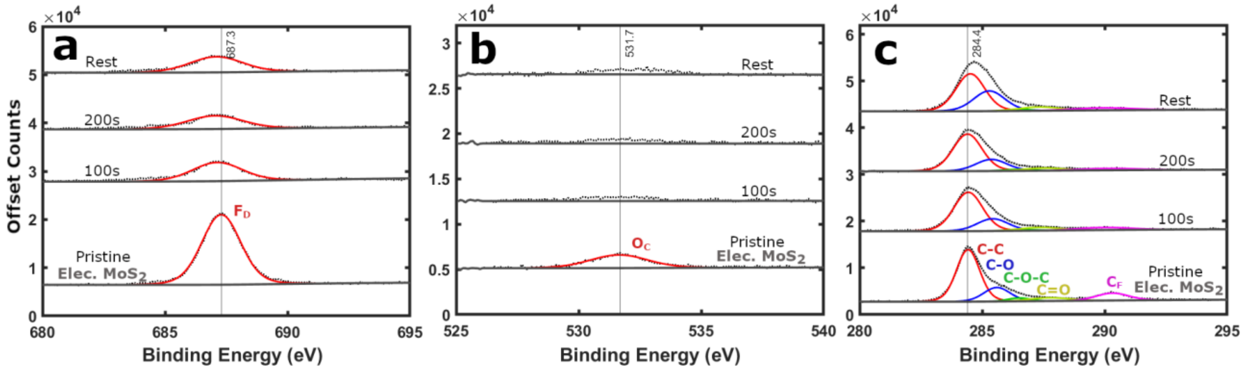

Figure S30. XPS of as-cast MoS_2 battery electrode (~ 36 μm) with rapid Ar^+ ion etching. Black dotted lines represent the raw XPS data, light grey solid lines depict the fitting envelope, and dark grey solid lines display the fitting background. (a) F 1s scan, (b) O 1s scan, and (c) C 1s scan regions.

The C 1s surface peaks are C-C/C-H (284.4 eV) from adventitious carbon and the conductive additive Super P, C-O/C-F (285.6 eV) from adventitious carbon and the PVDF binder, and the adventitious C-O-C (286.5 eV), C=O (287.7 eV), and C_F (O_2-C=O) (290.3 eV)[33–36] peaks.

MoS_2 LIB *Ex situ* Colored Rings' XPS

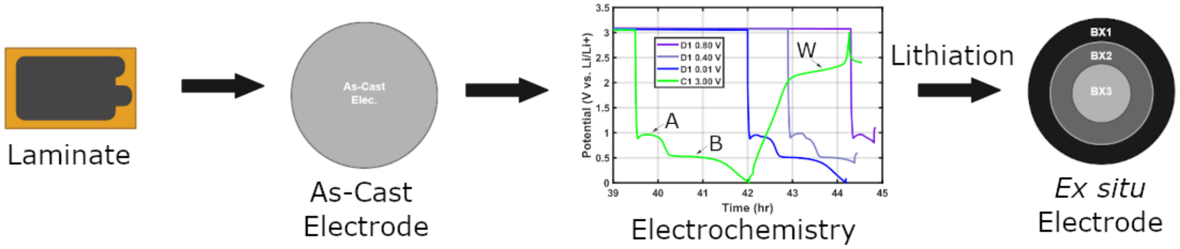

Figure S31. Schematic of a MoS_2 laminate being cut into 15 mm electrode discs, electrochemically tested (D1 0.01 V), and experiencing a tri-color ring pattern following lithiation in lithium-ion coin cell with a lithium metal counter electrode. Illustrations adapted with permission from source[23].

Note VII: *Ex situ* Lithiated MoS_2 Battery Electrode Sample Handling & XPS Preparation

The electrode discs (diameter 15 mm) used for the *ex situ* study all originate from the same electrode laminate (Figure S3) and are cut from the laminate center to avoid mass loading variability edge effects. The *ex situ* electrodes thus have similar active mass loadings of 3.69 mg/cm^2, 3.39 mg/cm^2, and 3.48 mg/cm^2, for D1 0.80 V, D1 0.01 V, and C1 3.00 V, respectively[23]. The overall electrode coating thickness is ~ 36 µm (± 4.6 µm)[23].

All three coin cells were assembled in the glovebox (O_2, H_2O < 0.5 ppm) at the same time and cycled over the same period at room temperature (Figure 8). The cells were programmed to finish their electrochemical testing at approximately the same time, by modulating their initial rest time accordingly (Figure 8). All three cells finished cycling within a 1 hour time period, and each cell was disassembled within the glovebox within 20 minutes of terminating their electrochemical protocol. All three *ex situ* electrodes were washed in fresh anhydrous DME within the glovebox and left to dry in an inert environment.

XPS samples were prepared by cutting the *ex situ* electrode discs with ceramic scissors in the glovebox and loading them onto an air-free XPS transfer stage. Note, the only deviation in sample handling occurs when conducting air-free XPS measurements. Due to the long-time frame required for sample etching, the *ex situ* electrodes were scanned in batches of 3 electrode locations per XPS session (e.g. AX1, AX2, and AX3).

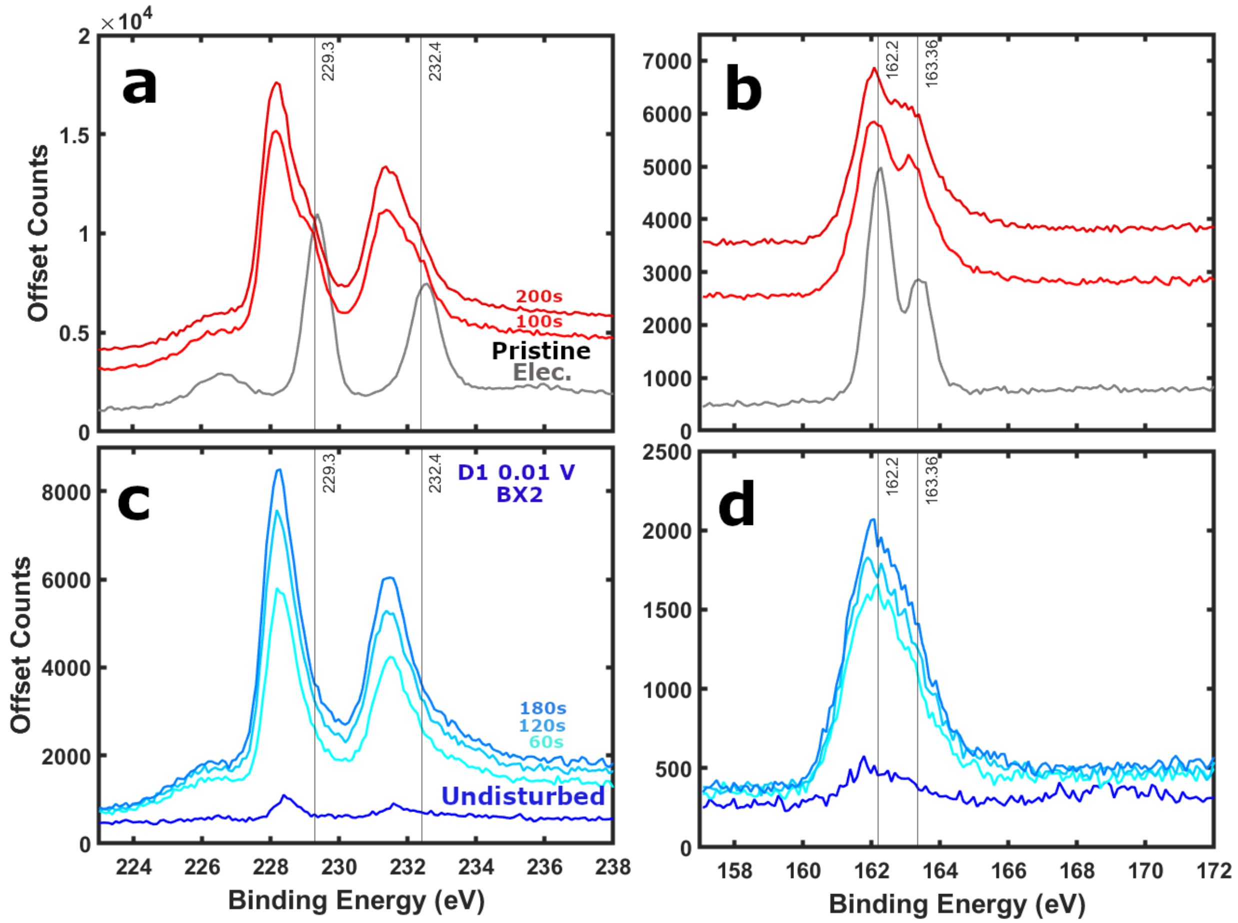

Figure S32. XPS Ar^+ depth-profiling of (a – b) as-cast MoS_2 electrode (c – d) and *ex situ* air-free lithiated middle colored ring BX2 after discharge to **D1 0.01V**.

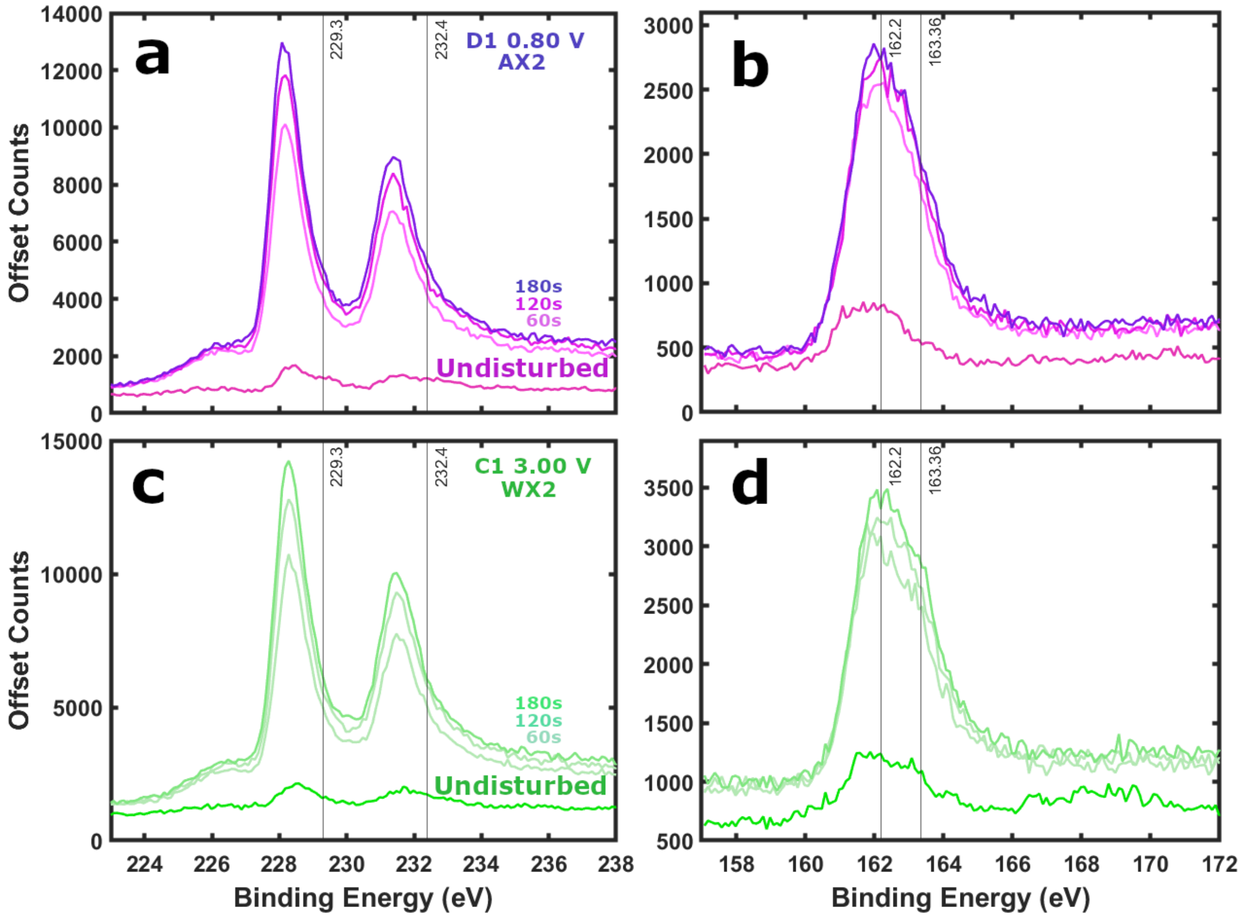

Figure S33. XPS Ar^+ depth-profiling of *ex situ* air-free middle colored rings (a – b) AX2 lithiated to **D1 0.80 V** (c – d) and WX2 delithiated to **C1 3.00 V**.

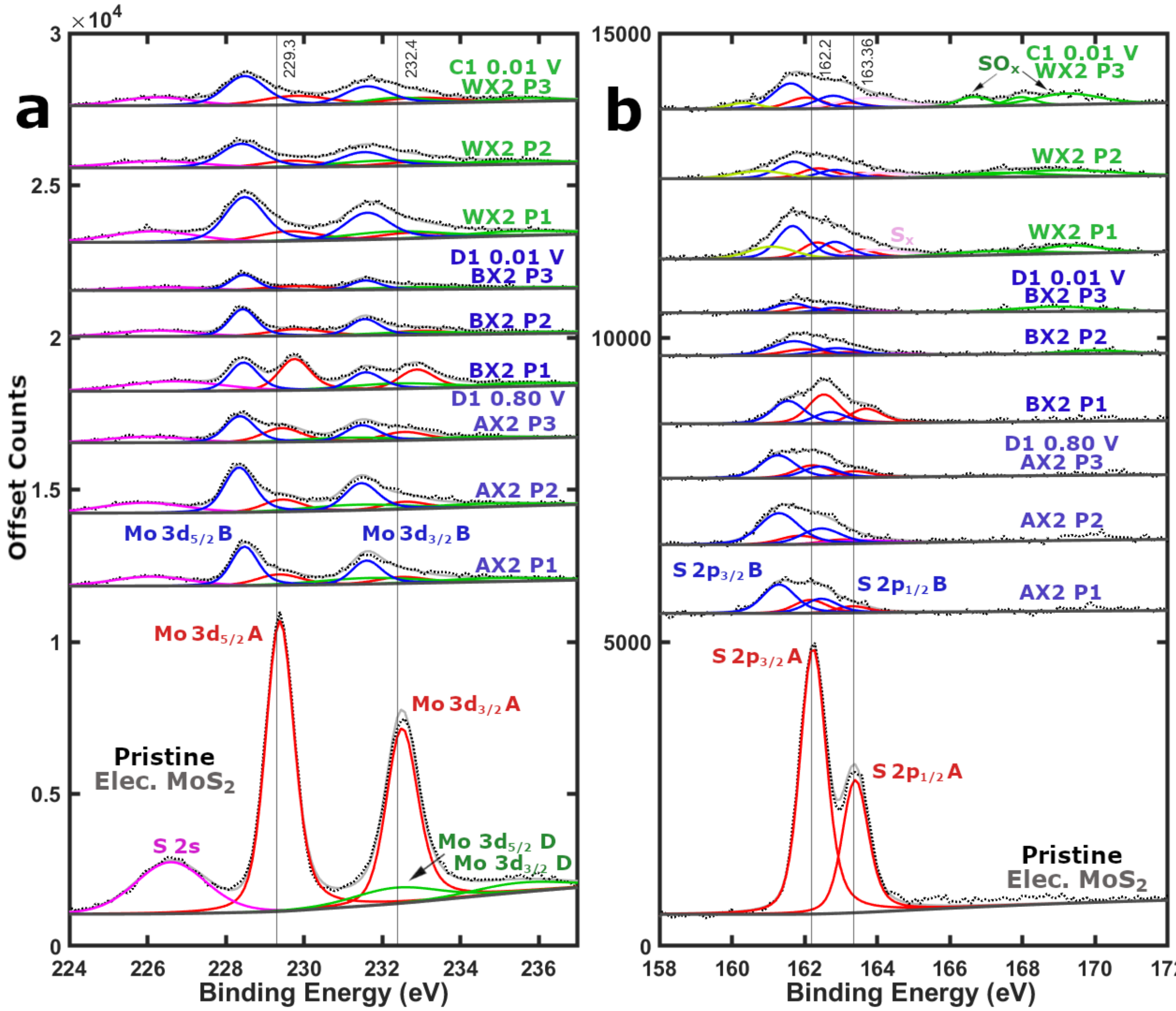

Figure S34. Undisturbed *ex situ* air-free surface XPS scans of the **middle colored rings** from ~ 36 µm thick MoS_2 electrodes lithiated (discharge – D1) to **D1 0.80 V** (AX2) and **D1 0.01 V** (BX2), or lithiated (D1 0.01 V) and delithiated (charge – C1) to **C1 3.00 V** (WX2) in a lithium-metal half-cell at a current density of 200 mA/g. Multiple positions scanned for consistency. Black dotted lines represent the raw XPS data, light grey solid lines depict the fitting envelope, and dark grey solid lines display the fitting background. Solid colored lines signify peak fitting. (a) Mo 3d and (b) S 2p scan regions. Data adapted with permission from source[23].

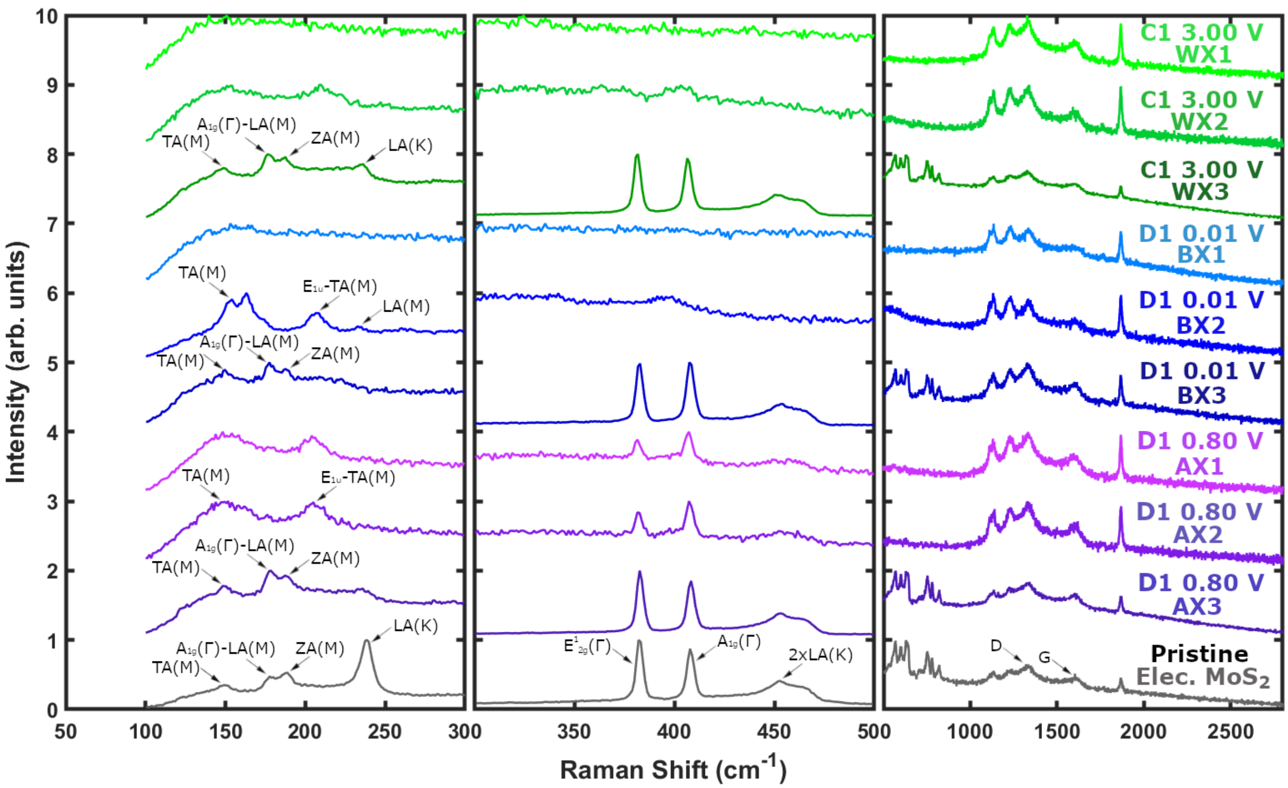

Figure S35. Undisturbed *ex situ* air-free surface Raman spectroscopy of the colored rings from ~ 36 µm thick MoS_2 electrodes lithiated (discharge – D1) to **D1 0.80 V** and **D1 0.01 V**, or lithiated (D1 0.01 V) and delithiated (charge – C1) to **C1 3.00 V** in a lithium-metal half-cell at a current density of 200 mA/g. Figure reprinted with permission from source[23].

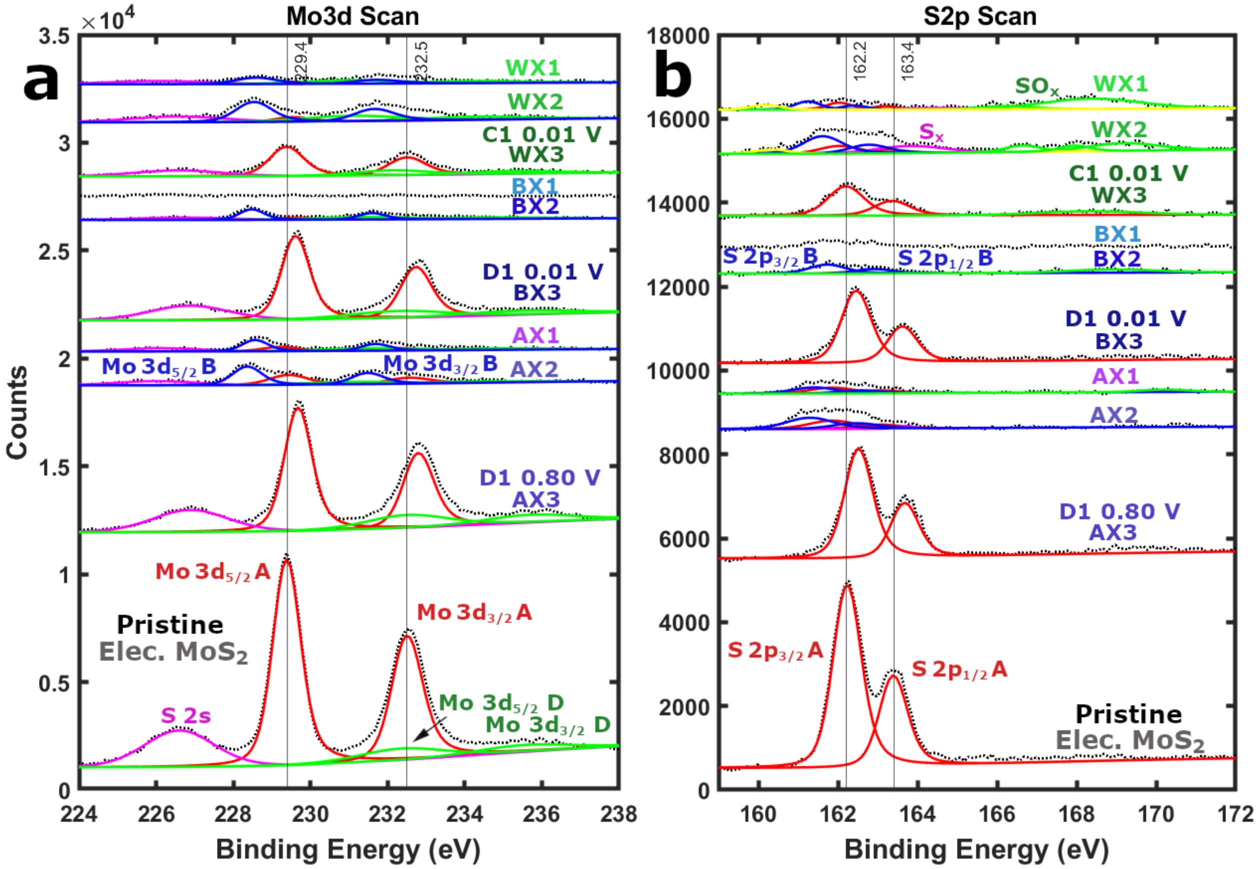

Figure S36. Undisturbed *ex situ* air-free surface XPS scans of the **colored rings** from ~ 36 µm thick MoS_2 electrodes lithiated (discharge – D1) to **D1 0.80 V** (AX1, AX2, & AX3) and **D1 0.01 V** (BX1, BX2, & BX3), or lithiated (D1 0.01 V) and delithiated (charge – C1) to **C1 3.00 V** (WX1, WX2, & WX3) in a lithium-metal half-cell at a current density of 200 mA/g. Black dotted lines represent the raw XPS data, light grey solid lines depict the fitting envelope, and dark grey solid lines display the fitting background. Solid colored lines signify peak fitting. (a) Mo 3d and (b) S 2p scan regions. Data reprinted with permission from source[23].

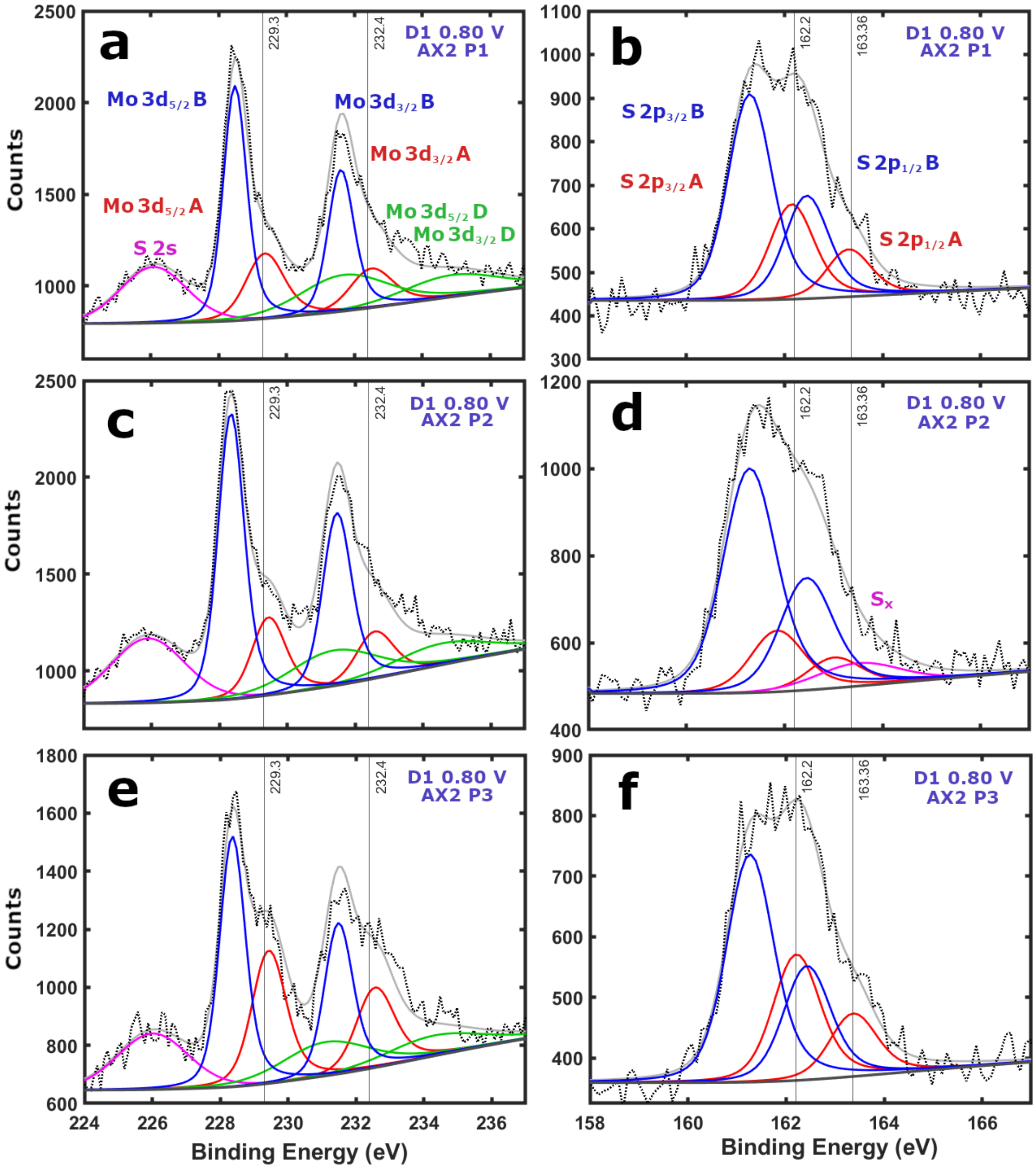

Figure S37. Undisturbed *ex situ* air-free surface XPS fitting of **middle colored ring** from ~ 36 µm thick MoS_2 electrodes lithiated (discharge – D1) to **D1 0.80 V** (AX2) in a lithium-metal half-cell at a current density of 200 mA/g. Black dotted lines represent the raw XPS data, light grey solid lines depict the fitting envelope, and dark grey solid lines display the fitting background. Three points analyzed for consistency. (a, c, & e) Mo 3d and (b, d, & f) S 2p scan regions. Data adapted with permission from source[23].

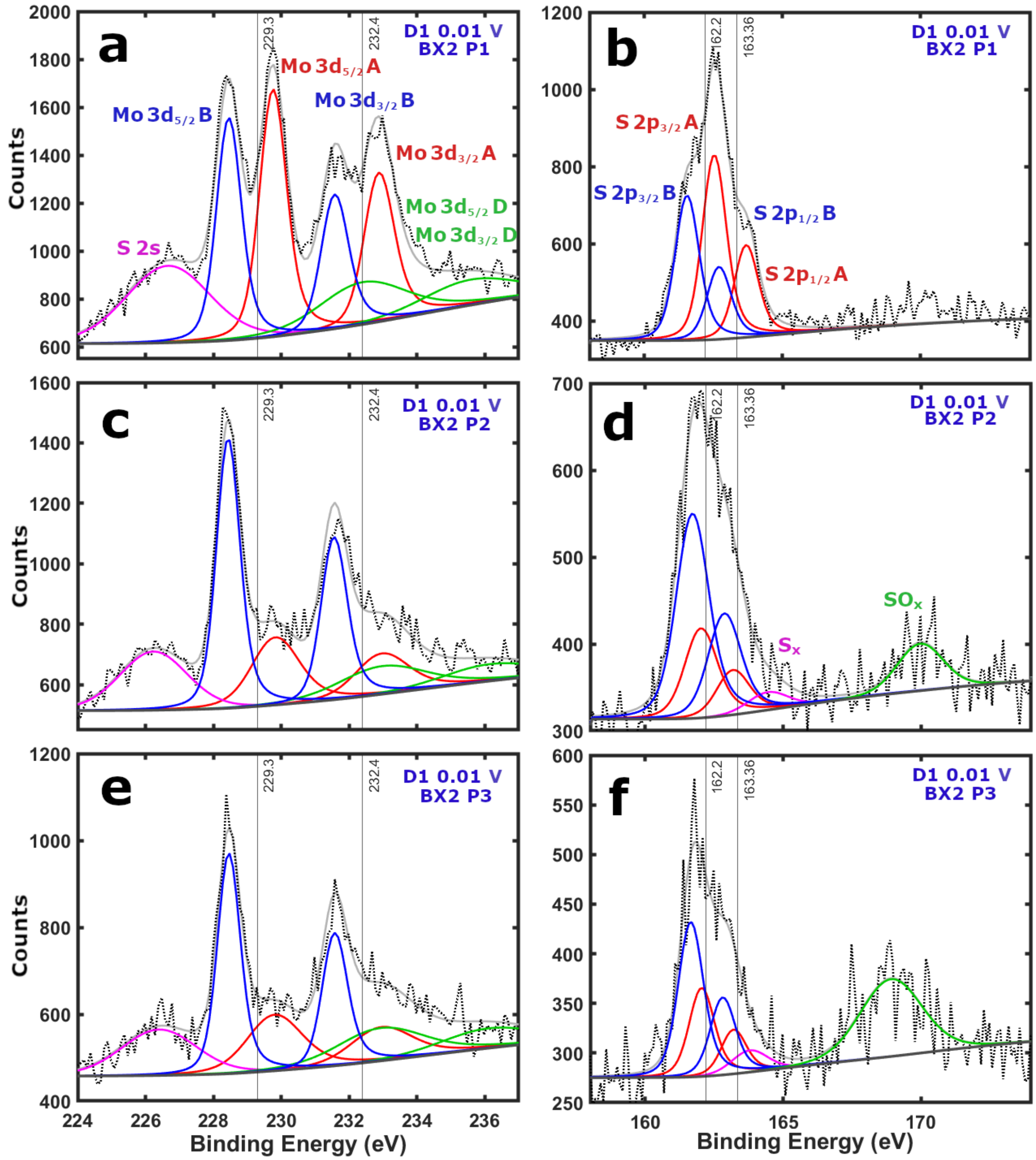

Figure S38. Undisturbed *ex situ* air-free surface XPS fitting of **middle colored ring** from ~ 36 μm thick MoS_2 electrodes lithiated (discharge – D1) to **D1 0.01 V** (BX2) in a lithium-metal half-cell at a current density of 200 mA/g. Black dotted lines represent the raw XPS data, light grey solid lines depict the fitting envelope, and dark grey solid lines display the fitting background. Three points analyzed for consistency. (a, c, & e) Mo 3d and (b, d, & f) S 2p scan regions. Data adapted with permission from source[23].

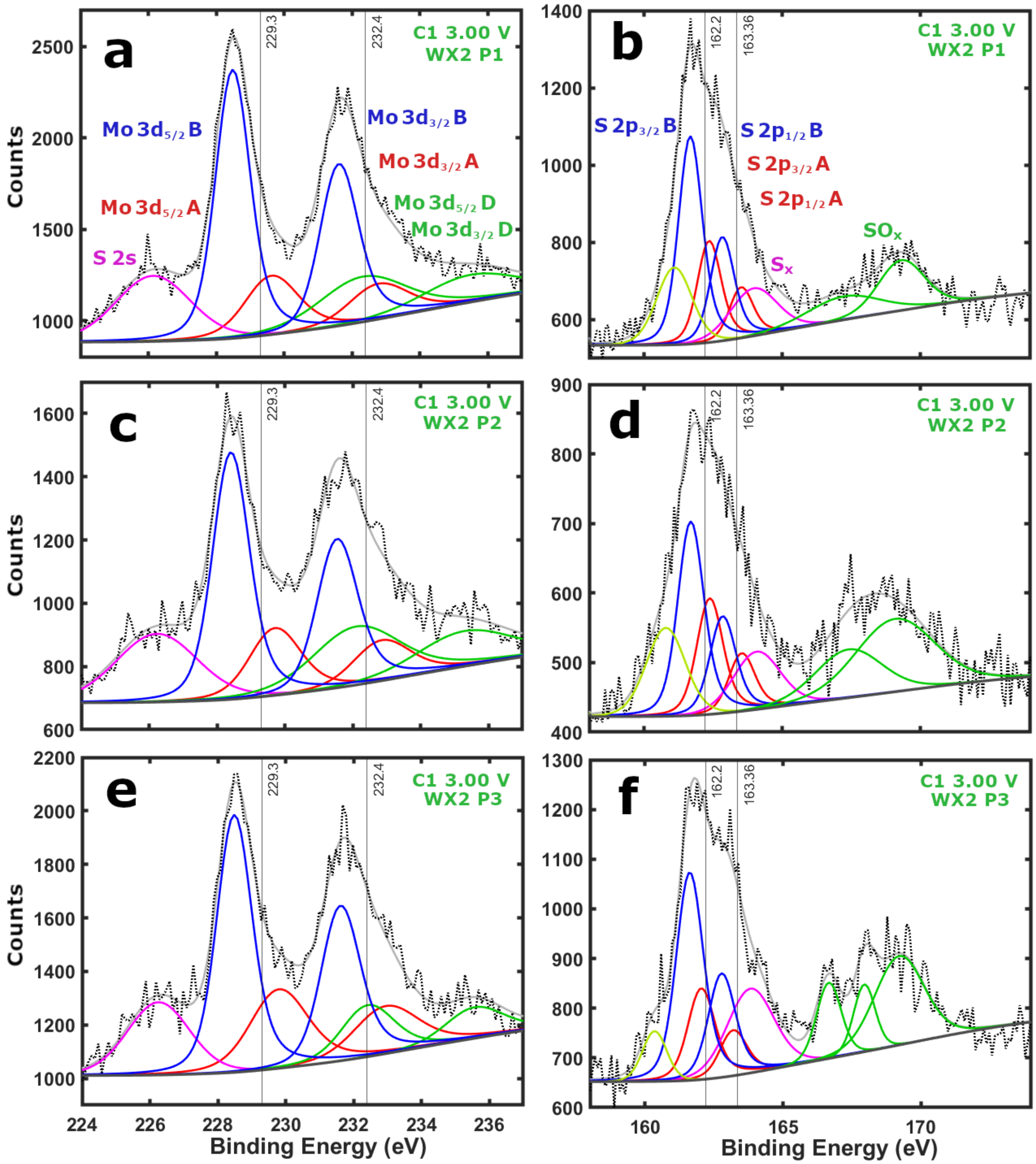

Figure S39. Undisturbed *ex situ* air-free surface XPS fitting of **middle colored ring** from ~ 36 μm thick MoS_2 electrodes lithiated (discharge – D1) to **D1 0.01 V** and then delithiated (charge – C1) to **C1 3.00 V** (WX2) in a lithium-metal half-cell at a current density of 200 mA/g. Black dotted lines represent the raw XPS data, light grey solid lines depict the fitting envelope, and dark grey solid lines display the fitting background. Three points analyzed for consistency. (a, c, & e) Mo 3d and (b, d, & f) S 2p scan regions. Data adapted with permission from source[23].

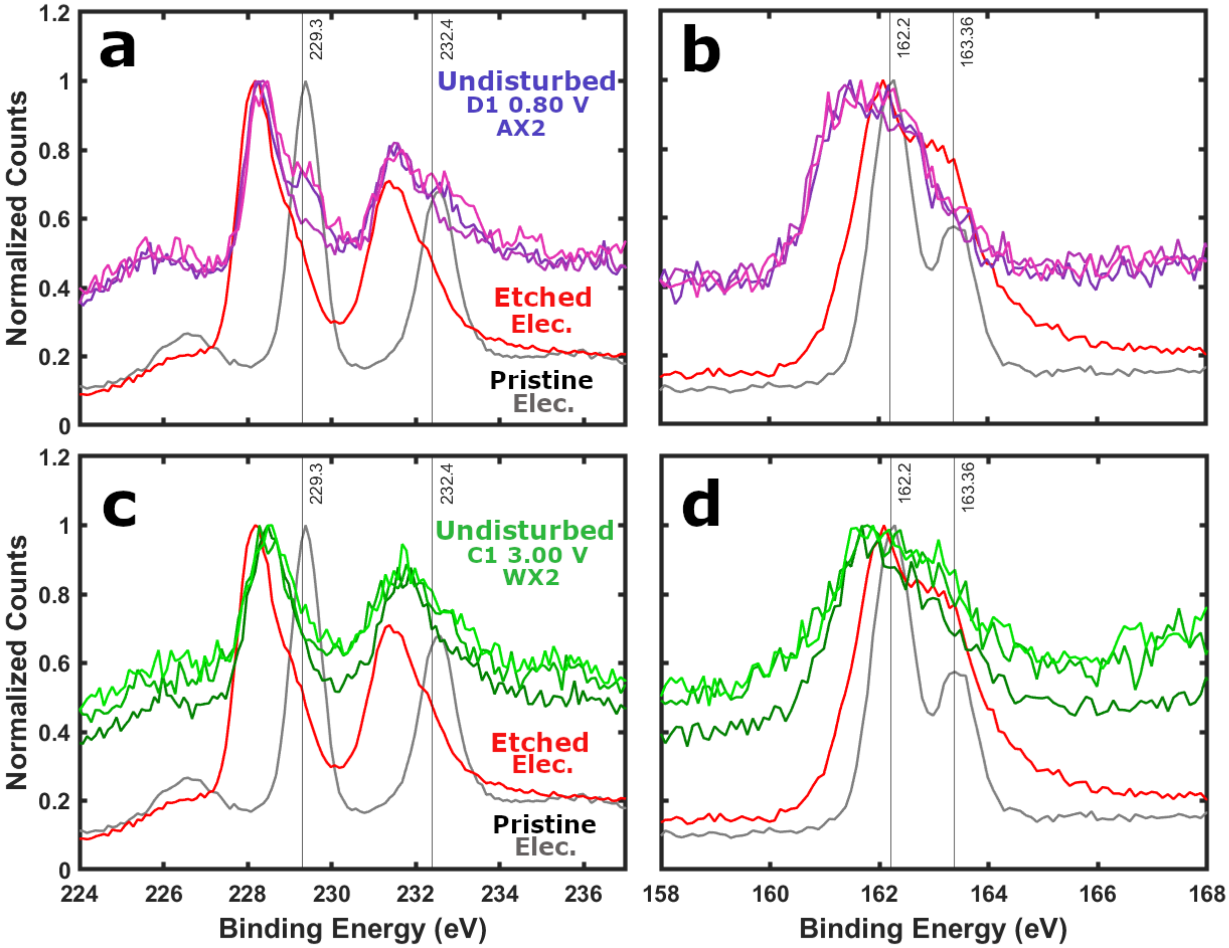

Figure S40. XPS comparison of undisturbed and ion bombarded (200s) as-cast MoS_2 electrode versus undisturbed air-free *ex situ* middle rings from (a – b) lithiated **D1 0.80 V** (**AX2**) and (c – d) delithiated **C1 3.00 V** (**WX2**) electrodes.

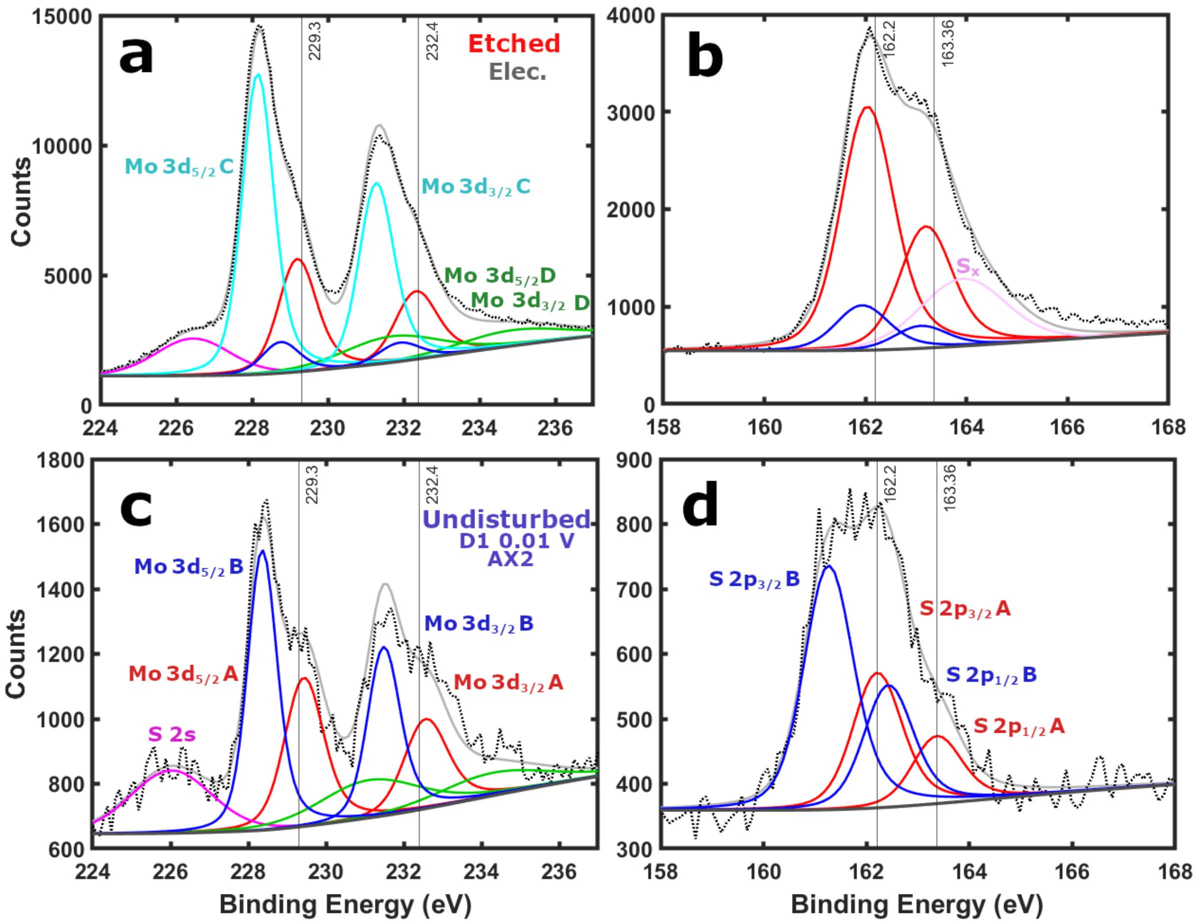

Figure S41. XPS of (a – b) ion bombarded (200s) as-cast MoS_2 electrode and (c – d) undisturbed air-free *ex situ* middle ring from lithiated **D1 0.80 V** (**AX2**) electrode.

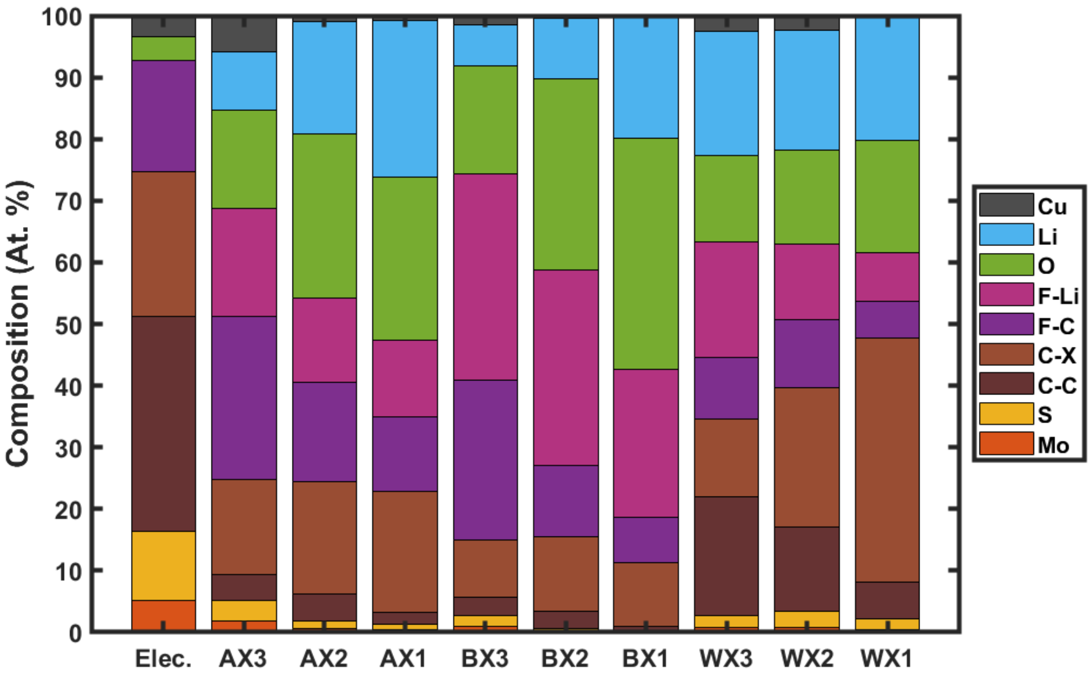

Figure S42. XPS atomic composition percentages for the undisturbed surface of the air-free *ex situ* LIB MoS_2 electrodes.

MoS_2 LIB *Ex situ* Colored Ring XPS Depth-Profiling

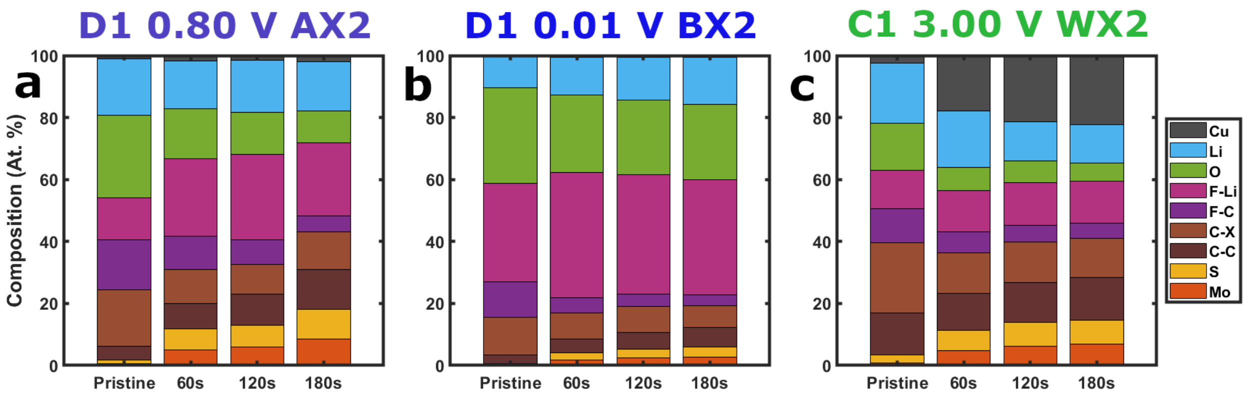

Figure S43. Air-free XPS atomic composition with depth-profiling of the **middle colored rings** of the *ex situ* LIB MoS_2 electrodes. (a) AX2, (b) BX2, and (c) WX2.

Note VIII: Electrode Copper Contamination in XPS

As highlighted by Figure S42, copper Cu 2p peaks can be detected on the undisturbed as-cast electrode surface. From the survey spectra (Figure S9) it is evident that the copper is not initially present in the powder form and only occurs after electrode slurry-casting. As the penetration depth of XPS is only 10 nm[26] in contrast to the much thicker as-cast electrode material coating (~ 36 μm) and since etching reduces the copper composition in the as-cast electrode (Figures S11b & S29), the copper detected must be present on the electrode surface. The possible sources of copper contamination include the slurry-casting coating process, electrode cutting with scissors during XPS sample preparation, electrode handling with gloves or tweezers, or XPS sample loading onto the stage.

When the copper contamination is only surface related, depth-profiling severely reduced the copper atomic percentage as occurs for the as-cast electrode (Figure S29) and *ex situ* electrodes AX2 and BX2 (Figures S43a – b). *Ex situ* electrode WX2 is the only exception, whereby the copper atomic percentage (Figure S43c) starkly increases with etching. As the copper substrate cannot be detected through the material coating and direct coating delamination cannot be the cause for the increase in copper as all the other species (Mo, S, F, O, and C) are still detected (Figure S33c – d), another reason for the increase in copper contamination must be present.

The surface of the *ex situ* electrode WX2 contains similar amounts of copper contamination from sample handling or preparation (Figure S44) as observed in the other samples. However, etching of WX2 results in a drastic increase in the copper presence (Figure S45). On closer inspection (Figure S46), the WX2 sample contained a small amount of exposed copper due to electrode coating delamination. In this case, the depth-profiling does not only include the sputtering of the material coating but also copper atoms from the exposed current collector. In this manner, the sample is cross-contaminated with copper and expresses a significantly larger atomic percentage than for any other electrode sample.

However, the additional copper contamination is not detrimental to the Mo 3d and S 2p region analysis, as the S/Mo ratios, Mo 3d percentages, and S 2p percentages are all normalized according to those regions. Therefore, the distinction between 2H MoS_2, 1T MoS_2, and MoS_{2-x} is still applicable. The only analysis that is hindered is the overall atomic composition with depth (Figure S43c).

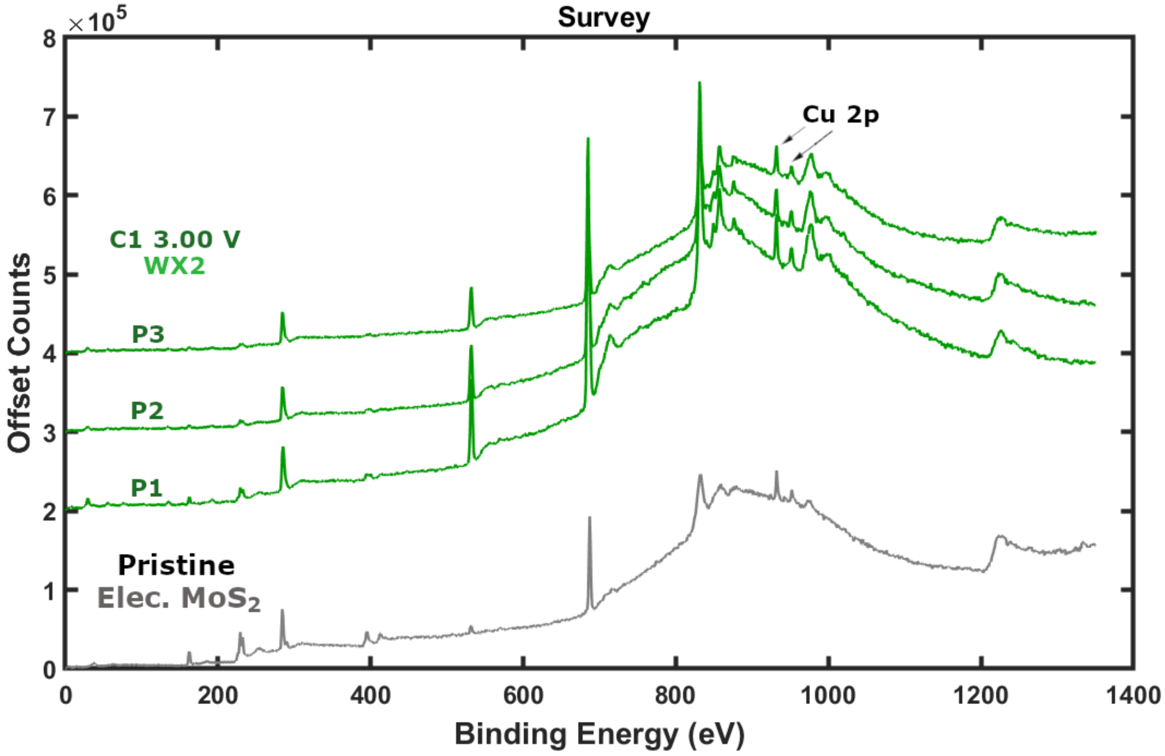

Figure S44. Offset survey spectra of as-cast electrode (pristine – grey) and WX2 undisturbed surface locations (undisturbed – green).

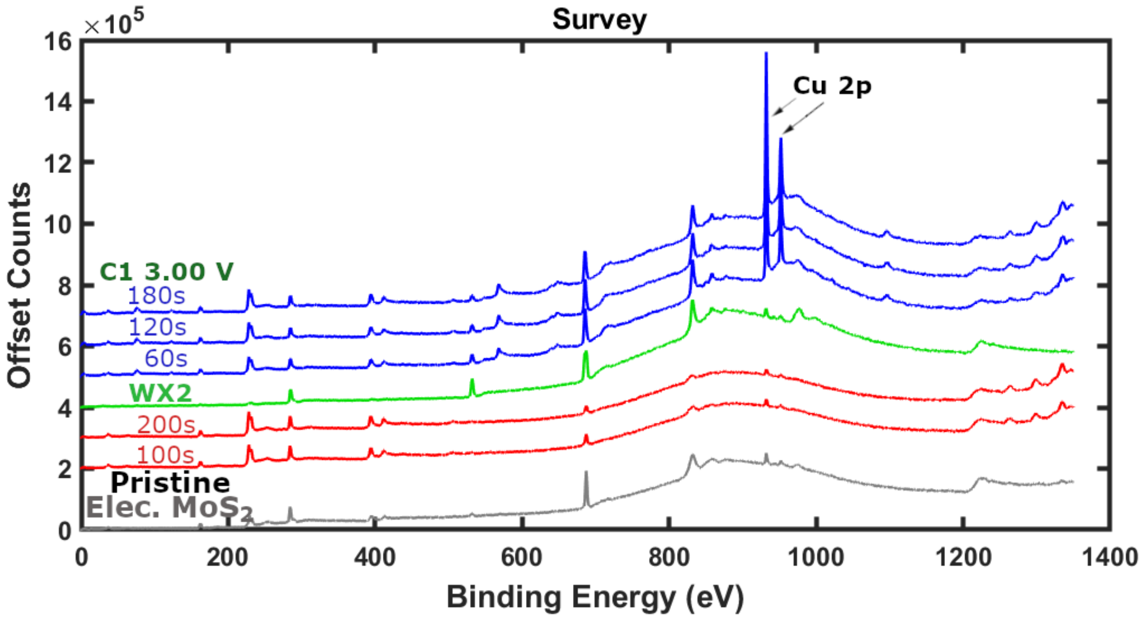

Figure S45. Offset survey spectra of as-cast electrode (pristine – grey, etched – red) and WX2 (undisturbed – green, etched – blue) before and after etching. The as-cast electrode is etched in increments of 100s and WX2 in increments of 60s.

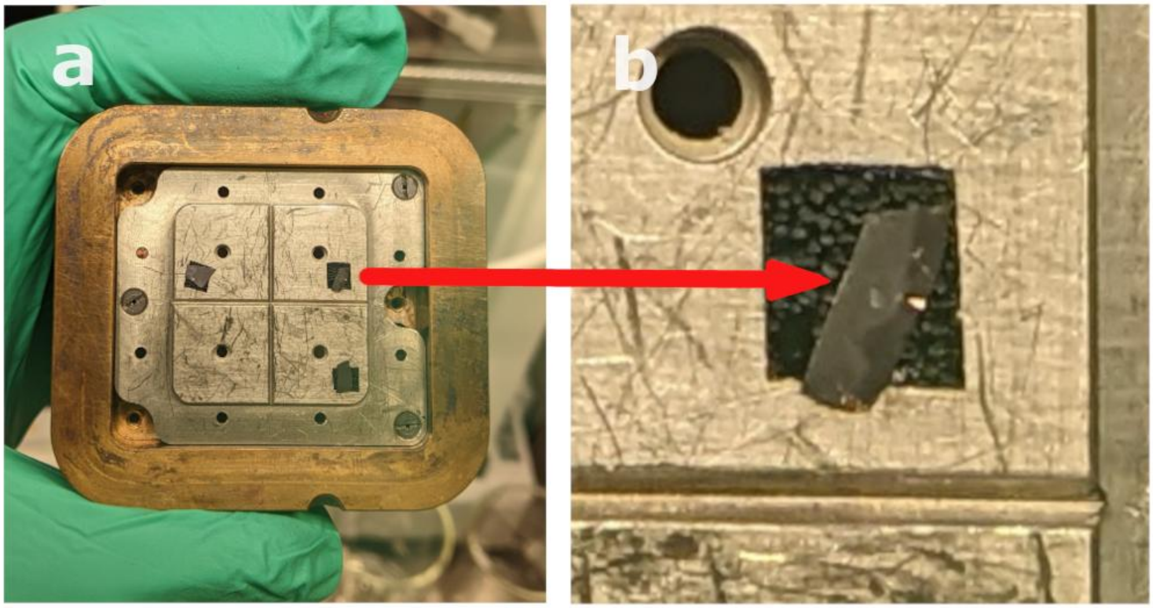

Figure S46. Digital images of samples on air-free XPS transfer stage. (a) WX3 (top-left), WX2 (top-right), and WX1 (bottom-right). (b) Zoomed in section of WX2 from (a).

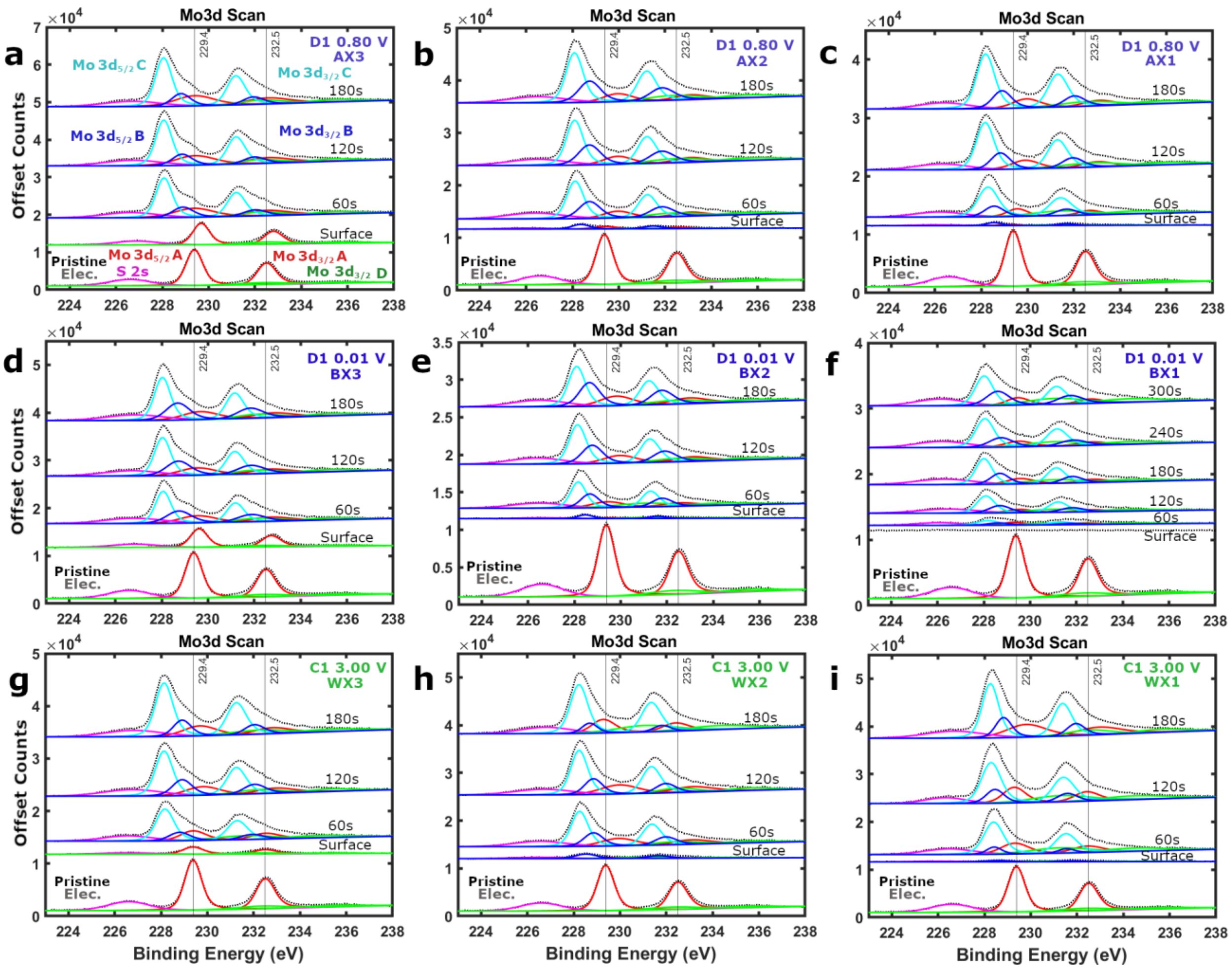

Figure S47. *Ex situ* air-free XPS of the Mo 3d region in the **colored rings** of ~ 36 µm thick MoS_2 electrodes cycled to **D1 0.80 V** (a – c), **D1 0.01 V** (d – f), and **C1 3.00 V** (g – i) in a lithium-metal half-cell at a current density of 200 mA/g. Ar^+ depth-profiling shown as total time. Black dotted lines represent the raw XPS data. Solid colored lines signify peak fitting. Figure taken with permission from source[23].

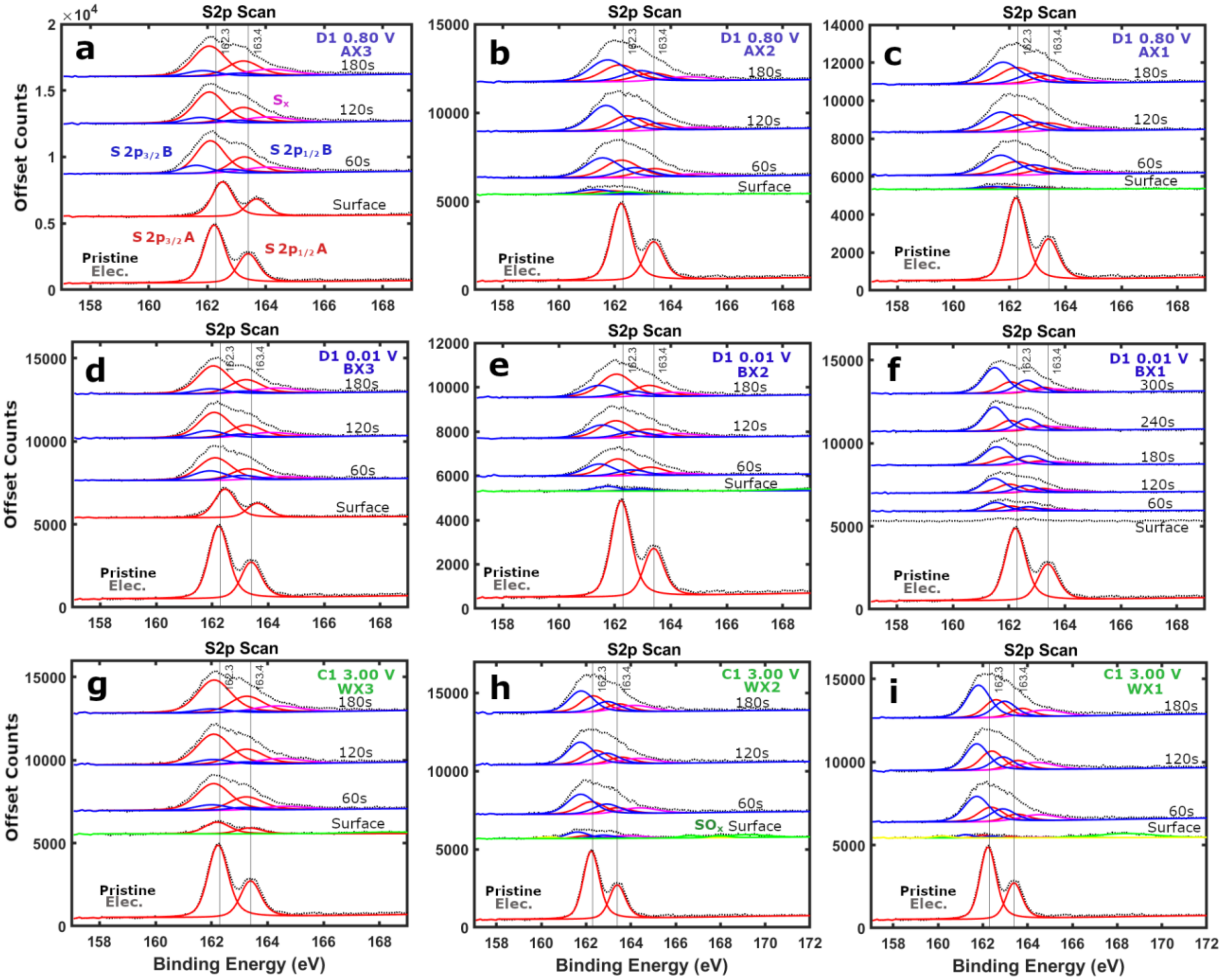

Figure S48. *Ex situ* air-free XPS of the S 2p region from the **colored rings** of ~ 36 µm thick MoS_2 electrodes lithiated/delithiated to **D1 0.80 V** (a – c), **D1 0.01 V** (d – f), and **C1 3.00 V** (g – i) in a lithium-metal half-cell at a current density of 200 mA/g. Ar^+ depth-profiling shown as total time. Black dotted lines represent the raw XPS data. Solid colored lines signify peak fitting. Figure reprinted with permission from source[23].

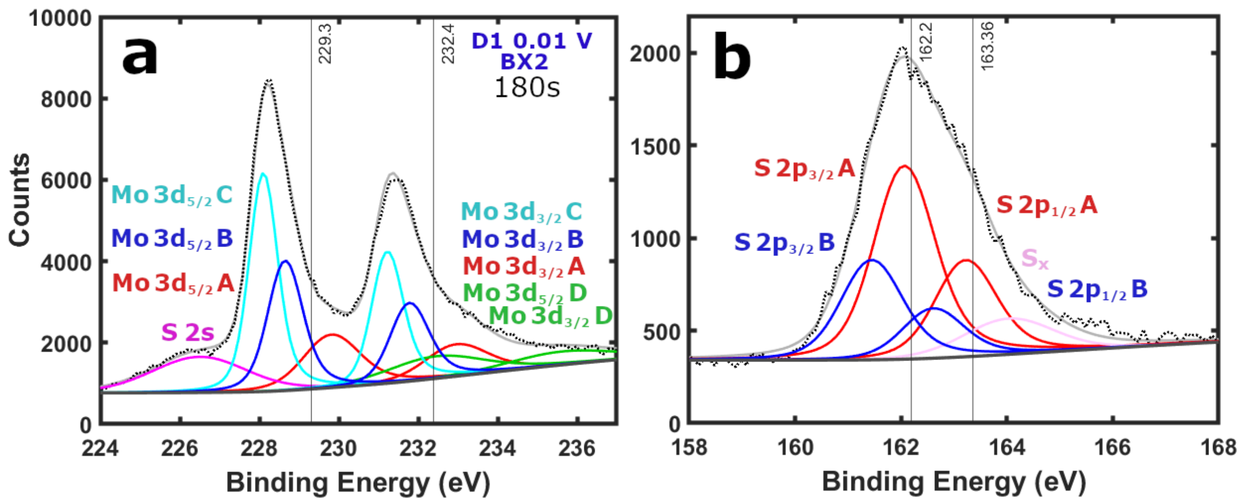

Figure S49. Residual plots for the four split orbit peak model applied to the BX2 *ex situ* air-free electrode depth-profiled by Ar^{+} ions. Cumulative etch time displayed (180s). Black dotted lines represent the raw XPS data, light grey solid lines depict the fitting envelope, and dark grey solid lines display the fitting background. (a) Mo 3d and (b) S 2p scan regions.

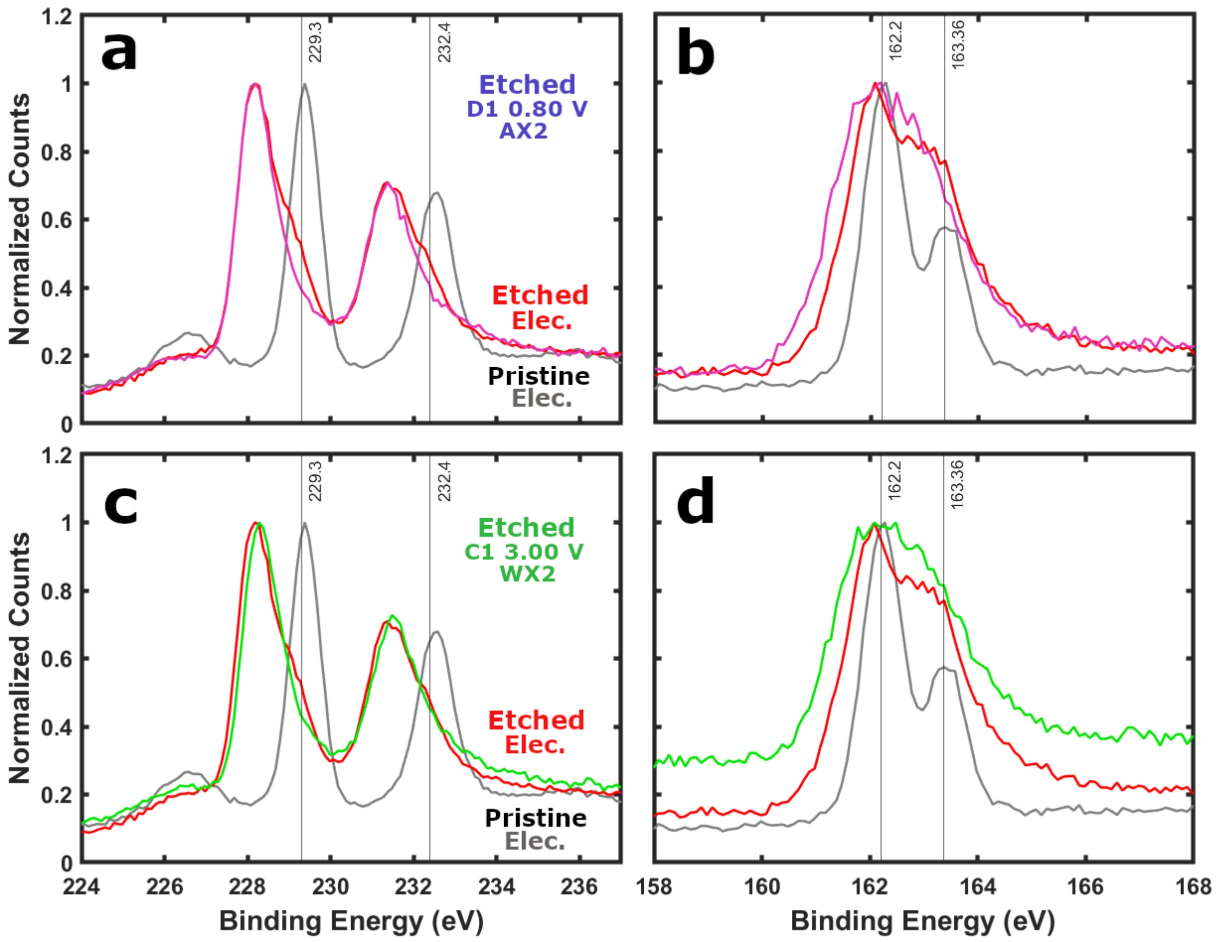

Figure S50. XPS comparison of undisturbed and ion bombarded (200s) as-cast MoS_2 electrode versus depth-profiled air-free *ex situ* middle rings from (a – b) lithiated **D1 0.80 V** (**AX2**) and (c – d) delithiated **C1 3.00 V** (**WX2**) electrodes.

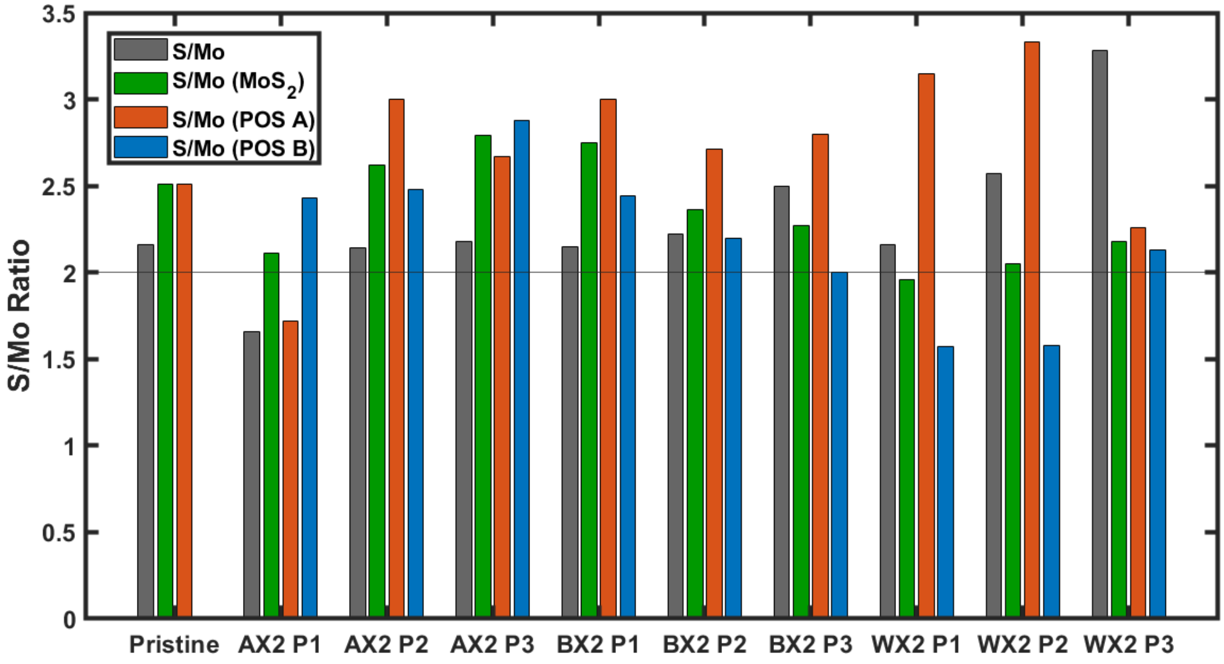

Figure S51. Air-free XPS S/Mo atomic ratios from the undisturbed surface of the *ex situ* LIB MoS_2 electrodes. S/Mo (grey) denotes the ratio of all Mo and S present in the sample including the Mo 3d MoO_3 specie, MoS_2 (green) represents all Mo and S collectively across all MoS_2 phases, whereas POS-A (red) and POS-B (blue – without S 2s) represent Mo and S exclusively detected in the 2H and 1T MoS_2 phases, respectively.

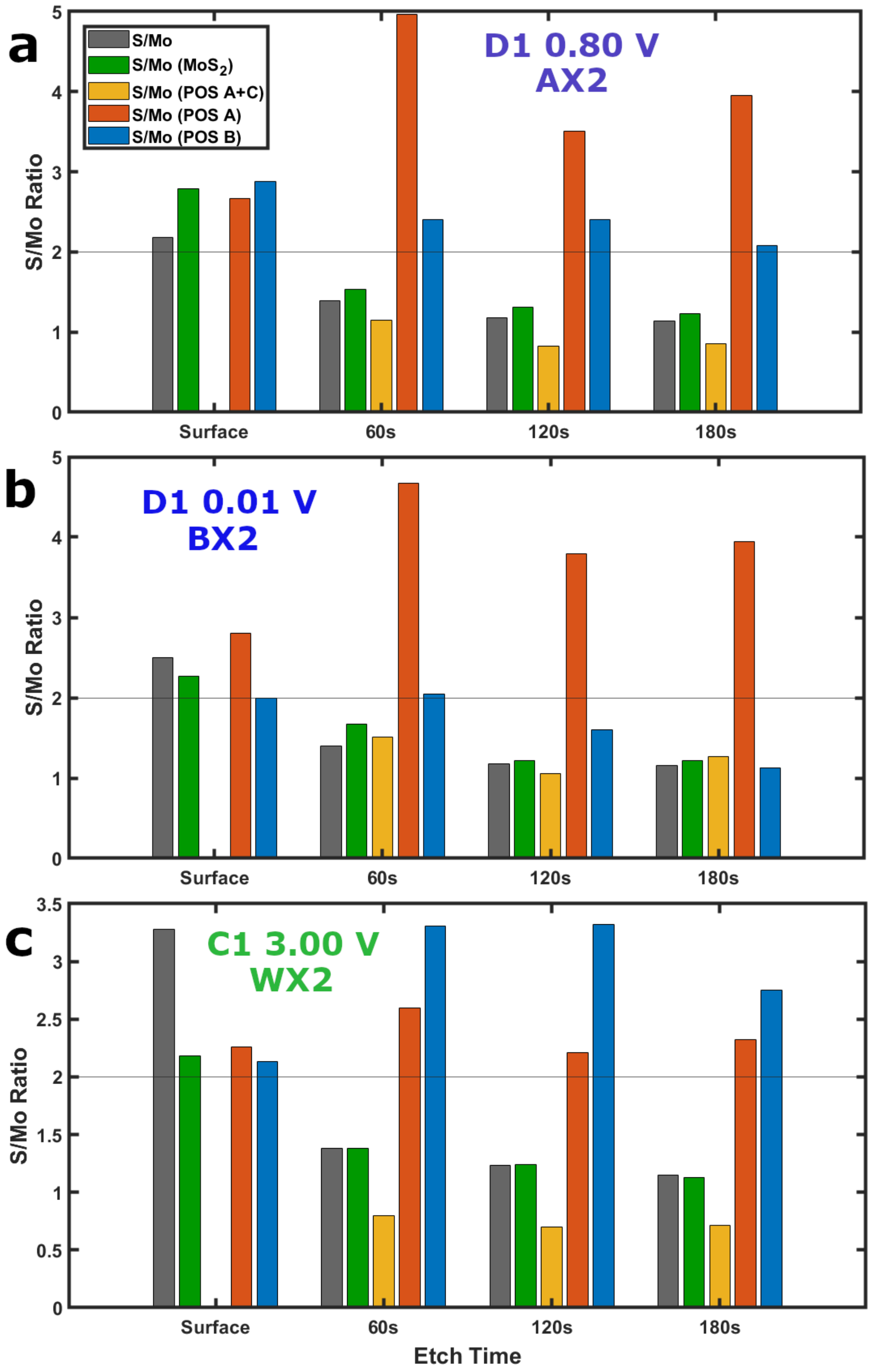

Figure S52. S/Mo air-free XPS atomic ratios with depth-profiling of the *ex situ* **colored rings** of ~ 36 µm thick MoS_2 electrodes cycled to (a) **D1 0.80 V**, (b) **D1 0.01 V**, and (c) **C1 3.00 V** in lithium-metal half-cells at a current density of 200 mA/g. S/Mo (grey) denotes the ratio of all Mo and S present in the sample including the Mo 3d MoO_3 specie, MoS_2 (green) represents all Mo and S collectively across all MoS_2 phases, whereas POS-A (red), POS-B (blue – without S 2s), and POS A + C (yellow) represent Mo and S detected in the 2H MoS_2, 1T MoS_2, and MoS_{2-x} phases, respectively.

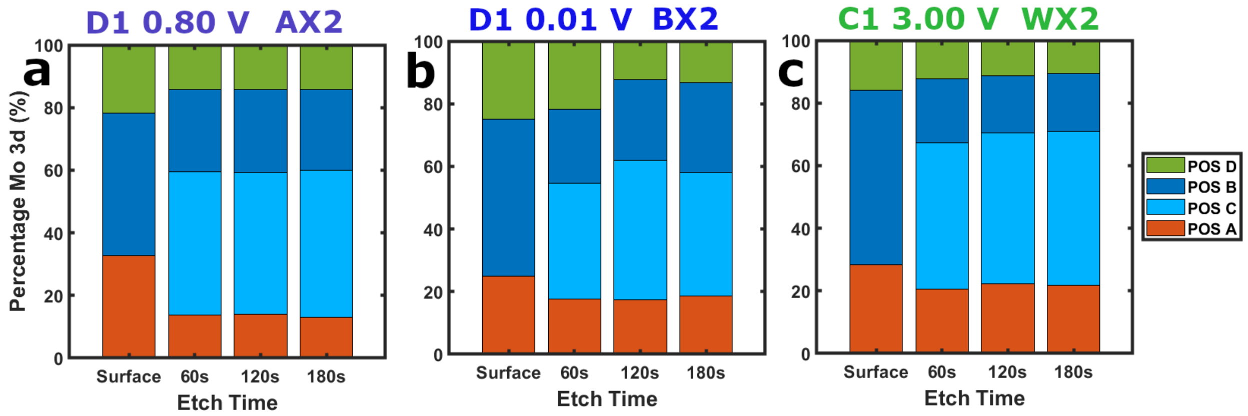

Figure S53. *Ex situ* air-free XPS of the Mo 3d **percentage** in the **middle rings** of ~ 36 µm thick MoS_2 electrodes cycled to (a) **D1 0.80 V**, (b) **D1 0.01 V**, and (c) **C1 3.00 V** in lithium-metal half-cells at a current density of 200 mA/g.

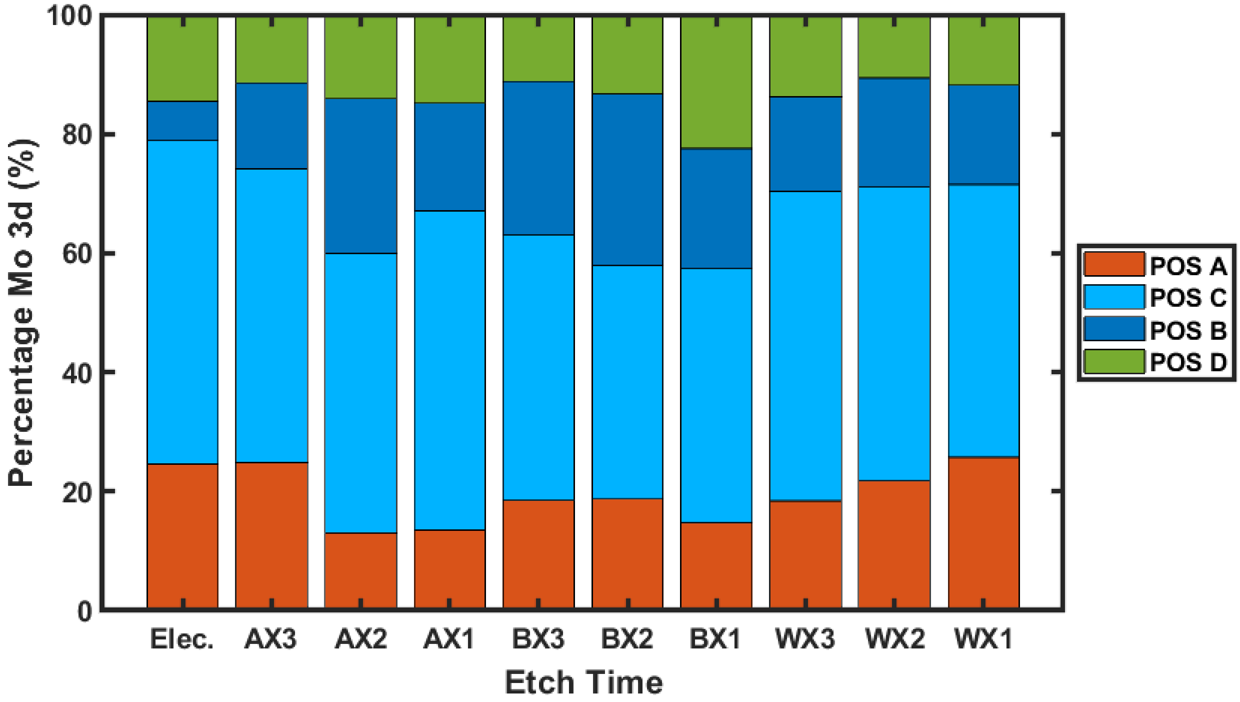

Figure S54. XPS atomic composition after 200s of Ar^+ ion etching for the as-cast electrode (Elec.) and 180s of depth-profiling for all *ex situ* LIB MoS_2 electrode colored rings.

Note IX: *Ex situ* Lithiated MoS_2 Battery Electrode XPS Fitting

Surface

The Mo 3d middle ring surface regions of all three lithiation states (**D1 0.80 V**, **D1 0.01 V**, and **C1 3.00 V**) are significantly altered (Figures 9a, S34, S38 – S40) relative to the as-cast electrode (Figure 7a), with the presence of major POS-B (228.4 eV) and reduced POS-A (229.6 eV) peaks.

Similarly, the sulfur S 2p region in the middle rings changes after lithiation (Figures 9b, S36, S40 – S42) in contrast to the as-cast electrode (Figure 7b), showing a clear second set of double-peaks (POS-B) at lower binding energies (161.5 eV). Therefore, with the presence of strong POS-B Mo 3d and S 2p peaks the middle rings possess a heterogenous 1T/2H MoS_2 phase surface.

The *ex situ* electrode surface S/Mo atomic ratios are elevated (~ 2.3) and vary widely (1.7 – 3.3 in Figure S51). Even within the same color ring, adjacent scan points can give drastically different atomic ratios due to the low presence (Figure S42) of detected molybdenum (< 1.05 %). Similarly, the POS-B S/Mo atomic ratio is elevated after discharge (~ 2.4) and low after charge (~1.8). However, relying solely on the POS-B S/Mo atomic ratio can be misleading due to the small amount of 1T MoS_2 available on the surface.

Depth-profiling

With depth-profiling of any electrode ring (Figure S47), in the Mo 3d region major POS-C peaks form, smaller POS-B peaks are observed, and minor POS-A peaks are retained. Simultaneously, the S 2p region broadens with the inclusion of POS-A and POS-B double-peaks. At a glance, this trend is similar to the sample alteration observed in the as-cast MoS_2 electrode (Figure 7) due to MoS_{2-x} generation. However, there is a need to accurately differentiate between solely MoS_{2-x} generation from argon bombardment of 2H MoS_2 and the possibility of preexisting 1T MoS_2 in the sample.

Initially, the S/Mo depth-profiling atomic ratios (Figure S52) might seem like a possible solution, as the 1T phase in the as-cast electrode retained a S/Mo atomic ratio of 2.1, whereas the 2H phase's atomic ratio reduced (Figure S27). However, the *ex situ* samples begin with highly variable surface S/Mo atomic ratios (Figure S51) due to the small amount of molybdenum and sulfur on the surface and despite following a similar trend to the as-cast electrode, no further insight can be gained between the three states of charge (Figure S52).

As a clear distinction of phase peaks appears in the Mo 3d region (POS-A, POS-B, and POS-C), an alternative approach is to use the molybdenum phase percentages with depth-profiling (Figure S53). Relying on the molybdenum percentage baseline identified from etching the as-cast electrode for 200s (MoS_{2-x}; 54.2%, 1T MoS_2; 6.7%, 2H MoS_2; 24.6%, and MoO_3; 14.5% in Figure S28a), the different colored rings of each discharge state can be evaluated (Figure S54 & Table S3). All middle rings (18.4 – 28.8%) exceed the 1T % threshold of the as-cast electrode (Figure S54).

However, from our previous XRD bulk measurements it is known that the central rings are unaffected in depth after lithiation, as they remain crystalline[23]. Therefore, to test this methodology the central rings can be used as internal references for the respective middle and outer rings of the same state of lithiation/delithiation. Especially since for each state of lithiation/delithiation the

three rings were stored, prepared, and measured at the same time. Hence, accounting for differences incurred from deviations between XPS measurements, as could take place in the as-cast electrode.

Nevertheless, after etching the lithiated middle and outer rings do not always exceed their own internal reference in 1T Mo 3d % (Table S3). Additionally, the outermost ring almost always (BX1 and WX1) has a lower 1T %, despite being shown to be most electrochemically active on the surface[23]. Therefore, the 1T Mo % method contradicts with our previous XPS surface scans (Figure S36), Raman spectroscopy surface spectra (Figure S35), and bulk XRD measurements[23]. Therefore, neither the S/Mo method nor the Mo 3d 1T % analysis can yield consistent results that can be validated against an internal sample reference.

Table S3. XPS Mo 3d percentage of the different phases within each sample after Ar^+ depth-profiling.

	**Depth-Profiling**	**POS-A**	**POS-C**	**POS-B**	**POS-D**
**Elec.**	As-cast	86.0	0.0	0.0	14.0
	100s	25.9	49.9	**8.7**	15.5
	200s	24.6	54.2	**6.7**	14.5
**AX3**	180s	24.9	49.3	**14.5**	11.4
**BX3**	180s	18.5	44.6	**25.8**	11.1
**WX3**	180s	18.4	51.9	**16.0**	13.7
**AX2**	180s	13.1	46.9	**25.9**	14.1
**BX2**	180s	18.7	39.2	**28.8**	13.3
**WX2**	180s	21.8	49.3	**18.4**	10.6
**AX1**	180s	13.5	53.5	**18.1**	14.9
**BX1**	180s	14.7	42.6	**20.2**	22.5
**WX1**	180s	25.8	45.7	**16.7**	11.9

Table S4. XPS S 2p percentage of the different phases within each sample after Ar^+ depth-profiling.

	**Depth-Profiling**	**2H MoS_2**	**1T MoS_2**	**S_x**	**SO_x**
**Elec.**	As-cast	100.0	0.0	0.0	0.0
	100s	71.3	**13.4**	15.3	0.0
	200s	71.2	**11.8**	16.9	0.0
**AX3**	180s	74.6	**12.5**	12.9	0.0
**BX3**	180s	76.9	**12.1**	11.0	0.0
**WX3**	180s	79.6	**8.8**	11.5	0.0
**AX2**	180s	45.3	**47.4**	7.2	0.0
**BX2**	180s	63.5	**27.9**	8.7	0.0
**WX2**	180s	43.9	**44.0**	12.1	0.0
**AX1**	180s	43.7	**47.4**	8.9	0.0
**BX1**	180s	35.7	**54.1**	10.2	0.0
**WX1**	180s	38.1	**51.5**	10.4	0.0

Table S5. XPS S 2p percentage of the different phases within *ex situ* MoS_2 LIB electrodes.

	Depth-Profiling	2H MoS_2	1T MoS_2	S_x	SO_x
**Elec.**	0s	100.0	0.0	0.0	0.0
**AX3**	0s	100.0	0.0	0.0	0.0
**BX3**	0s	100.0	0.0	0.0	0.0
**WX3**	0s	83.2	0.0	0.0	16.8
**AX2**	0s	45.1	52.5	2.5	0.0
**BX2**	0s	17.6	52.9	2.0	27.5
**WX2**	0s	21.3	35.2	21.7	21.7
**AX1**	0s	46.7	**47.4**	3.3	10.0
**BX1**	0s	-	-	-	-
**WX1**	0s	19.7	**17.3**	5.2	57.8

References

(1) Santoni, A.; Rondino, F.; Malerba, C.; Valentini, M.; Mittiga, A. Electronic Structure of Ar^+ Ion-Sputtered Thin-Film MoS_2: A XPS and IPES Study. *Appl Surf Sci* 2017, *392*, 795–800. https://doi.org/10.1016/j.apsusc.2016.09.007.

(2) Fang, X.; Guo, X.; Mao, Y.; Hua, C.; Shen, L.; Hu, Y.; Wang, Z.; Wu, F.; Chen, L. Mechanism of Lithium Storage in MoS_2 and the Feasibility of Using Li2S/Mo Nanocomposites as Cathode Materials for Lithium-Sulfur Batteries. *Chem Asian J* 2012, *7* (5), 1013–1017. https://doi.org/10.1002/asia.201100796.

(3) Fang, X.; Hua, C.; Guo, X.; Hu, Y.; Wang, Z.; Gao, X.; Wu, F.; Wang, J.; Chen, L. Lithium Storage in Commercial MoS_2 in Different Potential Ranges. *Electrochim Acta* 2012, *81*, 155–160. https://doi.org/10.1016/j.electacta.2012.07.020.

(4) Wang, L.; Xu, Z.; Wang, W.; Bai, X. Atomic Mechanism of Dynamic Electrochemical Lithiation Processes of MoS_2 Nanosheets. *J Am Chem Soc* 2014, *136* (18), 6693–6697. https://doi.org/10.1021/ja501686w.

(5) Zhu, Z.; Tang, Y.; Leow, W. R.; Xia, H.; Lv, Z.; Wei, J.; Ge, X.; Cao, S.; Zhang, Y.; Zhang, W.; Zhang, H.; Xi, S.; Du, Y.; Chen, X. Approaching the Lithiation Limit of MoS_2 While Maintaining Its Layered Crystalline Structure to Improve Lithium Storage. *Angewandte Chemie* 2019, *131* (11), 3559–3564. https://doi.org/10.1002/ange.201813698.

(6) Choi, W.; Choi, Y. S.; Kim, H.; Yoon, J.; Kwon, Y.; Kim, T.; Ryu, J. H.; Lee, J. H.; Lee, W.; Huh, J.; Kim, J. M.; Yoon, W. S. Evidence for the Coexistence of Polysulfide and Conversion Reactions in the Lithium Storage Mechanism of MoS_2 Anode Material. *Chem Mat* 2021, *33* (6), 1935–1945. https://doi.org/10.1021/acs.chemmater.0c02992.

(7) Wang, X.; Zhang, Z.; Chen, Y.; Qu, Y.; Lai, Y.; Li, J. Morphology-Controlled Synthesis of MoS_2 Nanostructures with Different Lithium Storage Properties. *J Alloys Compd* 2014, *600*, 84–90. https://doi.org/10.1016/J.JALLCOM.2014.02.127.

(8) Liu, T.; Melinte, G.; Dolotko, O.; Knapp, M.; Mendoza-Sánchez, B. Activation of 2D MoS_2 Electrodes Induced by High-Rate Lithiation Processes. *J Energy Chem* 2022, *78*, 56–70. https://doi.org/10.1016/j.jechem.2022.11.007.

(9) Zhou, J.; Qin, J.; Zhang, X.; Shi, C.; Liu, E.; Li, J.; Zhao, N.; He, C. 2D Space-Confined Synthesis of Few-Layer MoS_2 Anchored on Carbon Nanosheet for Lithium-Ion Battery Anode. *ACS Nano* 2015, *9* (4), 3837–3848. https://doi.org/10.1021/nn506850e.

(10) Halankar, K. K.; Mandal, B. P.; Jangid, M. K.; Mukhopadhyay, A.; Abharana, N.; Nayak, C.; Dasgupta, K.; Tyagi, A. K. Improved Electrochemical Performance of Interface Modified MoS_2/CNT Nano-Hybrid and Understanding of Its Lithiation/Delithiation Mechanism. *J Alloys Compd* 2020, *844*, 156076. https://doi.org/10.1016/j.jallcom.2020.156076.

(11) Li, J.; Hou, Y.; Gao, X.; Guan, D.; Xie, Y.; Chen, J.; Yuan, C. A Three-Dimensionally Interconnected Carbon Nanotube/Layered MoS_2 Nanohybrid Network for Lithium Ion Battery Anode with Superior Rate Capacity and Long-Cycle-Life. *Nano Energy* 2015, *16*, 10–18. https://doi.org/10.1016/j.nanoen.2015.05.025.

(12) Pan, F.; Wang, J.; Yang, Z.; Gu, L.; Yu, Y. MoS_2-Graphene Nanosheet-CNT Hybrids with Excellent Electrochemical Performances for Lithium-Ion Batteries. *RSC Adv* 2015, *5* (95), 77518–77526. https://doi.org/10.1039/c5ra13262b.

(13) Yoo, H.; Tiwari, A. P.; Lee, J.; Kim, D.; Park, J. H.; Lee, H. Cylindrical Nanostructured MoS_2 Directly Grown on CNT Composites for Lithium-Ion Batteries. *Nanoscale* 2015, *7* (8), 3404–3409. https://doi.org/10.1039/c4nr06348a.

(14) Zhang, Z.; Zhao, H.; Teng, Y.; Chang, X.; Xia, Q.; Li, Z.; Fang, J.; Du, Z.; Świerczek, K. Carbon-Sheathed MoS_2 Nanothorns Epitaxially Grown on CNTs: Electrochemical Application for Highly Stable and Ultrafast Lithium Storage. *Adv Energy Mater* 2018, *8* (7). https://doi.org/10.1002/aenm.201700174.

(15) Chang, K.; Chen, W. In Situ Synthesis of MoS_2/Graphene Nanosheet Composites with Extraordinarily High Electrochemical Performance for Lithium Ion Batteries. *Chem Com* 2011, *47* (14), 4252. https://doi.org/10.1039/c1cc10631g.

(16) Yang, L.; Wang, S.; Mao, J.; Deng, J.; Gao, Q.; Tang, Y.; Schmidt, O. G. Hierarchical MoS_2/Polyaniline Nanowires with Excellent Electrochemical Performance for Lithium-Ion Batteries. *Adv Mater* 2013, *25* (8), 1180–1184. https://doi.org/10.1002/adma.201203999.

(17) Hu, L.; Ren, Y.; Yang, H.; Xu, Q. Fabrication of 3D Hierarchical MoS_2/Polyaniline and MoS_2/C Architectures for Lithium-Ion Battery Applications. *ACS Appl Mater Interfaces* 2014, *6* (16), 14644–14652. https://doi.org/10.1021/am503995s.

(18) Guo, B.; Feng, Y.; Chen, X.; Li, B.; Yu, K. Preparation of Yolk-Shell MoS_2 Nanospheres Covered with Carbon Shell for Excellent Lithium-Ion Battery Anodes. *Appl Surf Sci* 2018, *434*, 1021–1029. https://doi.org/10.1016/j.apsusc.2017.11.018.

(19) Zhang, Z.; Wu, S.; Cheng, J.; Zhang, W. MoS_2 Nanobelts with (002) Plane Edges-Enriched Flat Surfaces for High-Rate Sodium and Lithium Storage. *Energy Storage Mater* 2018, *15*, 65–74. https://doi.org/10.1016/j.ensm.2018.03.013.

(20) Tian, Y.; Liu, X.; Cao, X.; Zhang, D.; Xiao, S.; Li, X.; Le, Z.; Li, X.; Li, H. Microwave-Assisted Synthesis of 1T MoS_2/Cu Nanowires with Enhanced Capacity and Stability as Anode for LIBs. *Chem Eng J* 2019, *374* (374), 429–436. https://doi.org/10.1016/j.cej.2019.05.174.

(21) Bai, J.; Zhao, B.; Zhou, J.; Si, J.; Fang, Z.; Li, K.; Ma, H.; Dai, J.; Zhu, X.; Sun, Y. Glucose-Induced Synthesis of 1T-MoS_2 /C Hybrid for High-Rate Lithium-Ion Batteries. *Small* 2019, *15* (14), 1–11. https://doi.org/10.1002/smll.201805420.

(22) Wang, L.; Zhang, Q.; Zhu, J.; Duan, X.; Xu, Z.; Liu, Y.; Yang, H.; Lu, B. Nature of Extra Capacity in MoS_2 Electrodes: Molybdenum Atoms Accommodate with Lithium. *Energy Storage Mater* 2019, *16* (March 2018), 37–45. https://doi.org/10.1016/j.ensm.2018.04.025.

(23) Marinov, A. D.; Shah, A. R.; Howard, C. A.; Cullen, P. L. Visible Lithiation Gradients of Bulk MoS_2 in Lithium-Ion Coin Cells. *J Mater Chem A Mater* 2025. https://doi.org/10.1039/D5TA02290H.

(24) Holder, C. F.; Schaak, R. E. Tutorial on Powder X-Ray Diffraction for Characterizing Nanoscale Materials. *ACS Nano* 2019, *13* (7), 7359–7365. https://doi.org/10.1021/acsnano.9b05157.

(25) Placidi, M.; Dimitrievska, M.; Izquierdo-Roca, V.; Fontané, X.; Castellanos-Gomez, A.; Pérez-Tomás, A.; Mestres, N.; Espindola-Rodriguez, M.; López-Marino, S.; Neuschitzer, M.; Bermudez, V.; Yaremko, A.; Pérez-Rodríguez, A. Multiwavelength Excitation Raman Scattering Analysis of Bulk and 2 Dimensional MoS_2: Vibrational Properties of Atomically Thin MoS2 Layers. *2d Mater* 2015. https://doi.org/10.1088/2053-1583/2/3/035006.

(26) van der Heide, P. *X-Ray Photoelectron Spectroscopy: An Introduction to Principles and Practices*; Wiley, 2012. https://doi.org/10.1002/9781118162897.

(27) McIntyre, N. S.; Spevack, P. A.; Beamson, G.; Briggs, D. Effects of Argon Ion Bombardment on Basal Plane and Polycrystalline MoS_2. *Surf Sci* 1990, *237* (1–3). https://doi.org/10.1016/0039-6028(90)90508-6.

(28) Addou, R.; McDonnell, S.; Barrera, D.; Guo, Z.; Azcatl, A.; Wang, J.; Zhu, H.; Hinkle, C. L.; Quevedo-Lopez, M.; Alshareef, H. N.; Colombo, L.; Hsu, J. W. P.; Wallace, R. M. Impurities and Electronic Property Variations of Natural MoS_2 Crystal Surfaces. *ACS Nano* 2015, *9* (9), 9124–9133. https://doi.org/10.1021/acsnano.5b03309.

(29) Isherwood, L. H.; Hennighausen, Z.; Son, S. K.; Spencer, B. F.; Wady, P. T.; Shubeita, S. M.; Kar, S.; Casiraghi, C.; Baidak, A. The Influence of Crystal Thickness and Interlayer Interactions on the Properties of Heavy Ion Irradiated MoS_2. *2d Mater* 2020, *7* (3). https://doi.org/10.1088/2053-1583/ab817b.

(30) Baker, M. A.; Gilmore, R.; Lenardi, C.; Gissler, W. XPS Investigation of Preferential Sputtering of S from MoS_2 and Determination of MoS_x Stoichiometry from Mo and S Peak Positions. *Appl Surf Sci* 1999, *150* (1), 255–262. https://doi.org/10.1016/S0169-4332(99)00253-6.

(31) Nicholson, E.; Serles, P.; Wang, G.; Filleter, T.; Davis, J. W.; Singh, C. V. Low Energy Proton Irradiation Tolerance of Molybdenum Disulfide Lubricants. *Appl Surf Sci* 2021, *567* (July), 150677. https://doi.org/10.1016/j.apsusc.2021.150677.

(32) Ghorbani-Asl, M.; Kretschmer, S.; Spearot, D. E.; Krasheninnikov, A. V. Two-Dimensional MoS_2 under Ion Irradiation: From Controlled Defect Production to Electronic Structure Engineering. *2d Mater* 2017, *4* (2). https://doi.org/10.1088/2053-1583/aa6b17.

(33) Chan, Y. J.; Vedhanarayanan, B.; Ji, X.; Lin, T. W. Doubling the Cyclic Stability of 3D Hierarchically Structured Composites of 1T-MoS_2/Polyaniline/Graphene through the Formation of LiF-Rich Solid Electrolyte Interphase. *Appl Surf Sci* 2021, *565* (July), 150582. https://doi.org/10.1016/j.apsusc.2021.150582.

(34) Lin, Y. H.; Wu, L. T.; Zhan, Y. T.; Jiang, J. C.; Lee, Y. L.; Jan, J. S.; Teng, H. Self-Assembly Formation of Solid-Electrolyte Interphase in Gel Polymer Electrolytes for High Performance Lithium Metal Batteries. *Energy Storage Mater* 2023, *61* (June), 102868. https://doi.org/10.1016/j.ensm.2023.102868.

(35) Wang, Z.; Dong, Y.; Li, H.; Zhao, Z.; Bin Wu, H.; Hao, C.; Liu, S.; Qiu, J.; Lou, X. W. D. Enhancing Lithium-Sulphur Battery Performance by Strongly Binding the Discharge Products on Amino-Functionalized Reduced Graphene Oxide. *Nat Commun* 2014, *5* (May). https://doi.org/10.1038/ncomms6002.

(36) Zhiqiang Zhu, Yuxin Tang, Zhisheng Lv, Jiaqi Wei, Yanyan Zhang, Renheng Wang, Wei Zhang, Huarong Xia, Mingzheng Ge, and X. C. Fluoroethylene Carbonate Enabling a Robust LiF-rich Solid Electrolyte Interphase to Enhance the Stability of the MoS_2 Anode for Lithium-Ion Storage. *Angewandte Chemie* 2018, *130* (14), 3718–3722. https://doi.org/https://doi.org/10.1002/anie.201712907.
